# The gene expression of CALD1, CDH2, and POSTN in fibroblast are related to idiopathic pulmonary fibrosis

**DOI:** 10.3389/fimmu.2024.1275064

**Published:** 2024-02-02

**Authors:** Shufei Wu, Mengying Liu, Mingrui Zhang, Xu Ye, Huimin Gu, Cheng Jiang, Huihui Zhu, Xiaoling Ye, Qi Li, Xinmei Huang, Mengshu Cao

**Affiliations:** ^1^ Department of Respiratory and Critical Care Medicine, Nanjing Drum Tower Hospital Clinical College of Traditional Chinese and Western Medicine, Nanjing University of Chinese Medicine, Nanjing, China; ^2^ Department of Respiratory and Critical Care Medicine, Nanjing Drum Tower Hospital, The Affiliated Hospital of Nanjing University Medical School, Nanjing, Jiangsu, China; ^3^ Department of Respiratory and Critical Care Medicine, Nanjing Drum Tower Hospital Clinical College of Nanjing Medical University, Nanjing, China; ^4^ Nanjing Institute of Respiratory Diseases, Nanjing, China

**Keywords:** idiopathic pulmonary fibrosis, RNA sequencing, caldesmon, N-cadherin, periostin

## Abstract

**Introduction:**

Idiopathic pulmonary fibrosis (IPF) is characterized by progressive lung dysfunction due to excessive collagen production and tissue scarring. Despite recent advancements, the molecular mechanisms remain unclear.

**Methods:**

RNA sequencing identified 475 differentially expressed genes (DEGs) in the TGF-β1-induced primary lung fibrosis model. Gene expression chips GSE101286 and GSE110147 from NCBI gene expression omnibus (GEO) database were analyzed using GEO2R, revealing 94 DEGs in IPF lung tissue samples. The gene ontology (GO) and pathway enrichment, Protein-protein interaction (PPI) network construction, and Maximal Clique Centrality (MCC) scoring were performed. Experimental validation included RT-qPCR, Immunohistochemistry (IHC), and Western Blot, with siRNA used for gene knockdown. A co-expression network was constructed by GeneMANIA.

**Results:**

GO enrichment highlighted significant enrichment of DEGs in TGF-β cellular response, connective tissue development, extracellular matrix components, and signaling pathways such as the AGE-RAGE signaling pathway and ECM-receptor interaction. PPI network analysis identified hub genes, including FN1, COL1A1, POSTN, KIF11, and ECT2. CALD1 (Caldesmon 1), CDH2 (Cadherin 2), and POSTN (Periostin) were identified as dysregulated hub genes in both the RNA sequencing and GEO datasets. Validation experiments confirmed the upregulation of CALD1, CDH2, and POSTN in TGF-β1-treated fibroblasts and IPF lung tissue samples. IHC experiments probed tissue-level expression patterns of these three molecules. Knockdown of CALD1, CDH2, and POSTN attenuated the expression of fibrotic markers (collagen I and α-SMA) in response to TGF-β1 stimulation in primary fibroblasts. Co-expression analysis revealed interactions between hub genes and predicted genes involved in actin cytoskeleton regulation and cell-cell junction organization.

**Conclusions:**

CALD1, CDH2, and POSTN, identified as potential contributors to pulmonary fibrosis, present promising therapeutic targets for IPF patients.

## 1 Introduction

Idiopathic pulmonary fibrosis (IPF) is the most common idiopathic interstitial pneumonia characterized by poor prognosis, with a median survival of only 3-5 years after definite diagnosis. Notably, this disease usually occurs in older people, presenting with unexplained chronic exertional dyspnea, cough, bibasilar inspiratory crackles, and digital clubbing (1, 2). The pathological features of IPF include the presence of fibroblastic foci, alveolar honeycombing and excessive collagen deposition (3), while the exact molecular triggers for those pathological changes remain rudimentary. Due to the unclear etiology and molecular mechanisms, there are currently few and limited effective therapies targeting the pathogenesis (1, 4). Currently, pirfenidone (5) and nintedanib (6) were approved for treatment of IPF by Food and Drug Administration (FDA). However, these medications can only slow down the progression of the disease and are unable to reverse established pulmonary fibrosis. Therefore, understanding the pathogenesis and developing potential therapies for IPF is an urgent priority.

Currently, the pathogenesis of idiopathic pulmonary fibrosis remains unknown, but it is well acknowledged that the transforming growth factor-β (TGF-β) pathway plays a crucial role in the development of IPF (7, 8). TGF-β1 has been identified as the principal activator responsible for inducing abnormal fibroblast proliferation and myofibroblast differentiation, which drives the remodeling of extracellular matrix (ECM) and induces epithelial-mesenchymal transition (EMT) and fibroblast-myofibroblast transition (FMT) of lung interstitial tissue, ultimately leading to progressive collagen deposition and severe lung malfunction (9, 10). The high presence of collagen and α-smooth muscle actin (α-SMA) stress fibers produced by myofibroblasts is involved in this process (11).

**Figure 1:**
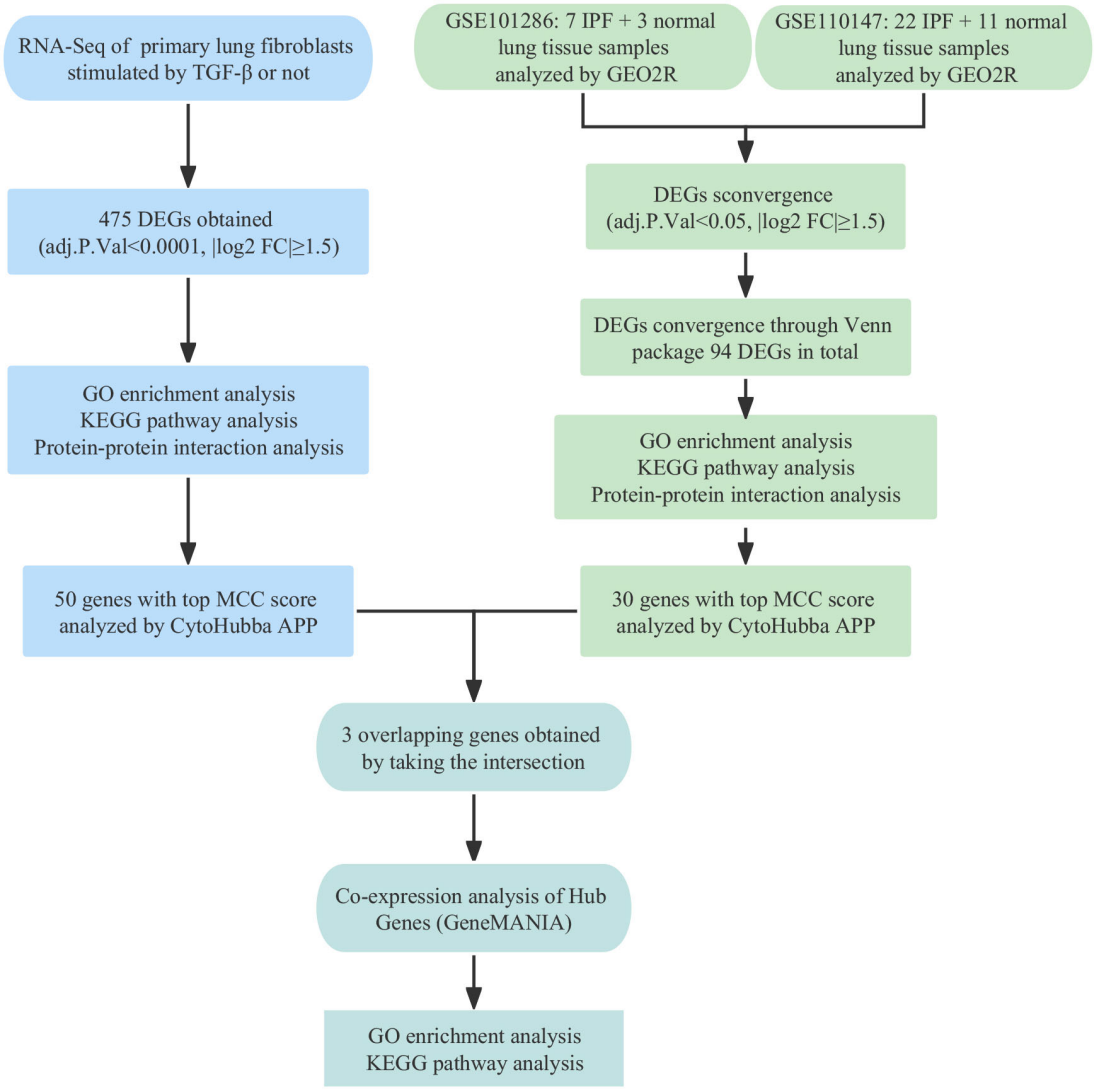
Schematic overview of study design.

The development and application of high-throughput sequencing technology and bioinformatics analysis have facilitated the identification of potential clinical pathogenic markers, which significantly improve the early diagnosis and prognosis of IPF (12). This study aimed to investigate the molecular mechanisms of IPF by analyzing differentially expressed genes (DEGs) from the TGF-β1 fibrosis model sequencing results and two mRNA expression profiling datasets from the GEO database. Gene Ontology, pathway enrichment, and protein-protein interaction network analyses were performed to explore the molecular function and mechanisms of IPF. CALD1, CDH2, and POSTN were identified as potential candidates through intersection analysis. Validation experiments confirmed their upregulation in TGF-β1-treated primary fibroblast and IPF tissue. Knockdown of CALD1, CDH2, and POSTN resulted in reduced expression of fibrosis markers in fibroblasts. Additionally, co-expression analysis revealed interactions between hub genes and predicted genes. The Schematic overview of this study was shown in **Figure 1**.

## 2 Materials and methods

### 2.1 Human primary fibroblast isolation and culture

The primary human lung fibroblasts (pHLF) were isolated from normal lung tissues with reference to literature (13). The normal lung tissues were obtained from patients with lung cancer resected and at least 1 centimeter far from the tumors. The pHLFs were cultured in DMEM/F12 with 10% fetal bovine serum (FBS) and penicillin-streptomycin mixture in standard culture conditions (5% CO2, 37°C). The growth medium was changed every 48 h. The cells of passages 3 were stimulated with recombinant human TGF-β1 (R&D Systems, USA, 5ng/ml) for 24 h.

### 2.2 Implementation and analysis of RNA sequencing

Three normal pHLF samples in six-well plates, as well as three pHLF samples treatment with recombinant TGF-β1 for 24 h, were collected in TRIzol reagent (15596026 and 15596018; Invitrogen, USA). The collected samples were sent to BGI-Shenzhen for RNA sequencing. The resulting data has been deposited in the NCBI Sequence Read Archive (SRA) database with the SRA accession number PRJNA842213. DEGs were obtained with the threshold criterion of adj.P.Val<0.0001, |log2 FC|≥1.5.

### 2.3 Microarray datasets collection and processing

GEO database (14) (https://www.ncbi.nlm.nih.gov/geo/) from the National Center for Biotechnology Information (NCBI) was implemented to search for homo sapiens and normal-control series with the keyword “idiopathic pulmonary fibrosis”. Two gene chips (GSE101286, GSE110147) were selected and gene expression profiling datasets were downloaded. apply GEO2R online tool (https://www.ncbi.nlm.nih.gov/geo/geo2r/) to conduct differential analyses of IPF compared to normal lung tissues. Volcano graphs were drawn with the threshold criterion of adj.P.Val<0.05, |log2 FC|≥1.5. The intersection genes were defined as differentially expressed genes displayed in the form of a Venn Diagram.

### 2.4 Functional and pathway enrichment analysis

In this study, genes were annotated according to the org.Hs.eg.db package (v.3.10.0) from R (v.3.6.3), and package cluster Profiler (v.3.14.3) was subsequently used to perform GO (Gene Ontology) and KEGG pathway analysis of differentially expressed genes in IPF patients, showing the adjusted P value (Benjamini-Hochberg correction). The core biological functions and pathways of the DEGs were visualized by with p.adj<0.05 & qvalue<0.2 as the cut-off criteria and Homo sapiens as the background. Z-scores were calculated and the chord plot was generated with the R package GOplot (v.3.1.0).

### 2.5 Protein-protein interaction analysis and co-expression network construction

The STRING 11.5 online database (15) (https://string-db.org/) was used to establish a protein-protein interaction network (PPI) of DEGs, and the results were imported into Cytoscape 3.9.1 software for visualization. Maximal Clique Centrality (MCC) method in the CytoHubba plugin of Cytoscape was utilized to further filter and generate predictions of hub genes consequently (16, 17).

GeneMANIA (18) (https://genemania.org) is an online analysis tool, which was used to construct the co-expression and pathway networks of key genes in IPF, and to predict the potential functions of hub genes in this study.

### 2.6 Lung tissue samples

Four normal lung tissue samples adjacent to cancer and 8 lung transplantation tissues from IPF patients were collected as described previously (11). Human lung tissue samples were perfused with 4% paraformaldehyde and subjected to paraffin embedding. Paraffin-embedded IPF lung tissue sections were exposed to Haematoxylin and eosin (H&E) staining to verify pulmonary fibrosis respectively. The current study was approved by the Ethics Committee of Nanjing Drum Tower Hospital in accordance with the Declaration of Helsinki (1989) (NO.2016-160-01). All subjects signed the informed consent paperwork.

### 2.7 Reverse transcription−quantitative PCR

Cellular and tissue mRNA was extracted by TRIzol Reagent, and cDNA synthesis was carried out with HiScript III RT SuperMix for qPCR (Vazyme, Nanjing, China) according to the manufacturer’s instructions. The RT-qPCR was conducted using 2×ChamQ Universal SYBR qPCR Master Mix (Vazyme, Nanjing, China). The primes were synthesized by Tsingke (Nanjing, China) listed in **Supplementary Table S1** in the **Supporting Information**. Glyceraldehyde-3-phosphate dehydrogenase (GAPDH) was used as an internal reference.

### 2.8 siRNA transfection

CALD1, CDH2, and POSTN were targeted for knockdown using small interfering RNA (siRNA) duplexes (2 duplexes per gene) along with a siRNA negative control (siNC). The siRNAs were synthesized by Shanghai GenePharma Co., Ltd. siRNAs are listed in **Supplementary Table S2**. Transfection of the siRNAs was carried out using GP-transfect-Mate Transfection reagent (GenePharma) according to the manufacturer’s instructions. The knockdown efficiency was assessed through RT-qPCR assay, and the siRNA demonstrating the most effective knockdown was chosen for further experiments.

### 2.9 Western blot

For western blot analysis, proteins were extracted using the RIPA buffer (Keygen), which was supplemented with protease and phosphatase inhibitors (Keygen). The protein concentration was determined using a BCA Protein Assay Kit (Biosharp). Equal amounts of lysate were loaded onto SDS-PAGE for separation and subsequently transferred to a PVDF membrane. Following a 1-hour block with 5% milk, the membranes were incubated with primary antibodies overnight, followed by a 1-hour incubation with peroxidase-coupled secondary antibodies at room temperature. The antibodies utilized in the western blot are detailed in **Supplementary Table S3**.

### 2.10 Immunohistochemistry

Immunohistochemistry staining of lung tissue sections was performed using an SP0041 kit (Beijing Solarbio Science Technology Co., Ltd). Antigen retrieval was achieved through citrate–EDTA buffer, followed by microwave treatment for 15 minutes. To mitigate non-specific staining, goat serum was utilized as a blocking agent. Subsequently, each appropriately diluted primary antibody was applied to each section and allowed to incubate overnight at 4°C. Following incubation with a secondary antibody, sections were visualized through a 2-minute exposure to diaminobenzidine (DAB). Counter-staining was achieved using Myer’s hematoxylin for 2 minutes, followed by dehydration and mounting. The Image-Pro Plus 6.0 software (IPP6) was employed for the analysis of the immunohistochemistry (IHC) results. Mean density was assessed, quantified as Integrated Optical Density (IOD) per unit area.

## 3 Results

### 3.1 Construction and analysis of TGF-β1 induced pulmonary fibroblast fibrosis model RNA sequencing data

**Figure 2:**
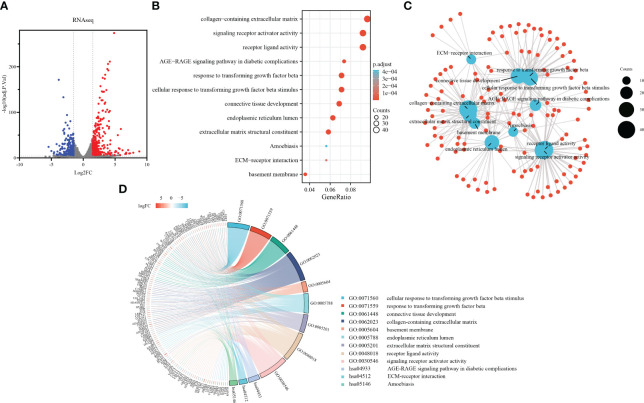
Development of a TGF-β1 induced pulmonary fibroblast fibrosis model: DEGs identification and functional enrichment analysis. **(A)** Volcano plot displaying DEGs identified from RNA sequence data using the criteria adj.P.Val<0.0001, |log2FC|≥1.5, in which red points represent up-regulated genes, and blue points represent down-regulated. **(B)** Bubble chart presenting Gene Ontology (GO) and pathway enrichment analysis of RNAseq-DEGs. Bubble size indicates counts of respective GO category, and bubble color represents the adjusted p-value. **(C)** Functional network of enrichment analysis constructed by clusterProfiler, in which red points represent genes involved in similar functional GO terms or pathways. **(D)** Chord diagram illustrating functional relationships among RNAseq-DEGs and GO terms.

Sirius red staining revealed increased collagen production in primary fibroblasts upon TGF-β1 stimulation (**Supplementary Figure S1A**). The protein expression of collagen I and α-SMA was confirmed by western blot analysis, with three biological replicates for each group (**Supplementary Figure S1B**). By analyzing the sequencing results, the 475 DEGs in the range of adj.P.Val<0.0001, |log2 FC|≥1.5 were screened out, as shown in the volcano plot (**Figure 2A**).

To further explore the functional implications of these DEGs, we performed enrichment analysis using the clusterProfiler R package. Notably, the Gene Ontology analysis results (**Figures 2B, C**) revealed significant enrichment of the DEGs in various biological processes, cellular components, molecular functions, and KEGG pathways. In terms of biological processes, the DEGs were involved in cellular response to TGF-β1 stimulus, response to transforming growth factor beta, connective tissue development, cartilage development, and striated muscle tissue development. Regarding cellular components, the DEGs were associated with collagen-containing extracellular matrix, basement membrane, endoplasmic reticulum lumen, complex of collagen trimers, and contractile fiber. In relation to molecular functions, the DEGs were implicated in extracellular matrix structural constituent, receptor ligand activity, signaling receptor activator activity, heparin binding, and collagen binding. Additionally, the DEGs were found to participate in various KEGG pathways, including the AGE-RAGE signaling pathway in diabetic complications, ECM-receptor interaction, Amoebiasis, PI3K-Akt signaling pathway, and Malaria. The chord plot showed the distribution of DEGs in different GO-enriched functions (**Figure 2D**). DEGs were presented on the left side with fold change values mapped by colorful scale, and gene involvement in the GO terms was determined by colored connecting lines.

### 3.2 DEGs identification and functional enrichment analysis in pulmonary fibrosis tissue gene expression profiles

**Figure 3:**
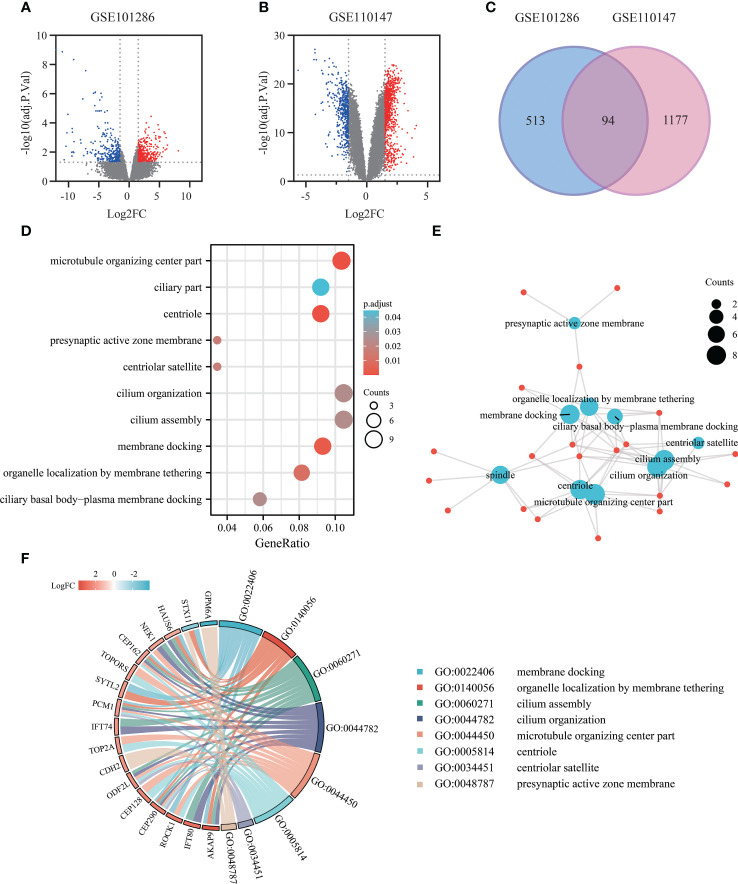
Exploring gene expression profiles in pulmonary fibrosis tissue: DEGs identification and functional enrichment analysis. **(A)** Volcano plot of DEGs (adj.P.Val<0.05, |log2FC|≥1.5) in mRNA expression profiling dataset GSE101286. **(B)** Volcano plot of DEGs (adj.P.Val<0.05, |log2FC|≥1.5) in dataset GSE110147. **(C)** Venn diagram depicting the overlapping DEGs, with 94 genes representing the intersection between the GSE101286 and GSE110147 datasets. **(D)** Bubble chart presenting the Gene Ontology (GO) and pathway enrichment analysis of DEGs in the Microarray analysis. **(E)** Functional network of enrichment analysis constructed using clusterProfiler. **(F)** Chord diagram visualizing the functional relationships among the DEGs and GO terms, highlighting the enrichment results from the GEO datasets.

A total of 7 IPF lung samples and 3 normal Lung tissue samples were measured in GSE101286 (Platform: GPL6947, Illumina HumanHT-12 V3.0 expression beadchip), and 22 IPF lung samples and 11 normal Lung tissue samples were measured in GSE110147 (Platform: GPL6244, Affymetrix Human Gene 1.0 ST Array). The genes were screened using GEO2R with the criteria of adj.P.Val<0.05, |log2 FC|≥1.5, resulting in two gene lists as shown in volcano plots (**Figures 3A, B**). After intersecting the 2 gene lists, 94 overlapping DEGs were acquired (**Figure 3C**).

To gain insights into the functional characteristics of these 94 DEGs, we processed the enrichment analysis of the 94 DEGs using clusterProfiler R package. Intriguingly, the Gene Ontology analysis results (**Figures 3D, E**) showed that the DEGs were mostly enriched in various cellular components, including microtubule organizing center part, centriole, spindle, presynaptic active zone membrane. As to the biological process groups, cilium organization, cilium assembly, membrane docking, organelle localization by membrane tethering, and ciliary basal body-plasma membrane docking were contained in the top 10 enrichment classes. The chord plot showed the distribution of DEGs in different GO-enriched functions, highlighting the importance of these processes in pulmonary fibrosis (**Figure 3F**).

### 3.3 Protein-protein interaction network analysis of DEGs

**Figure 4:**
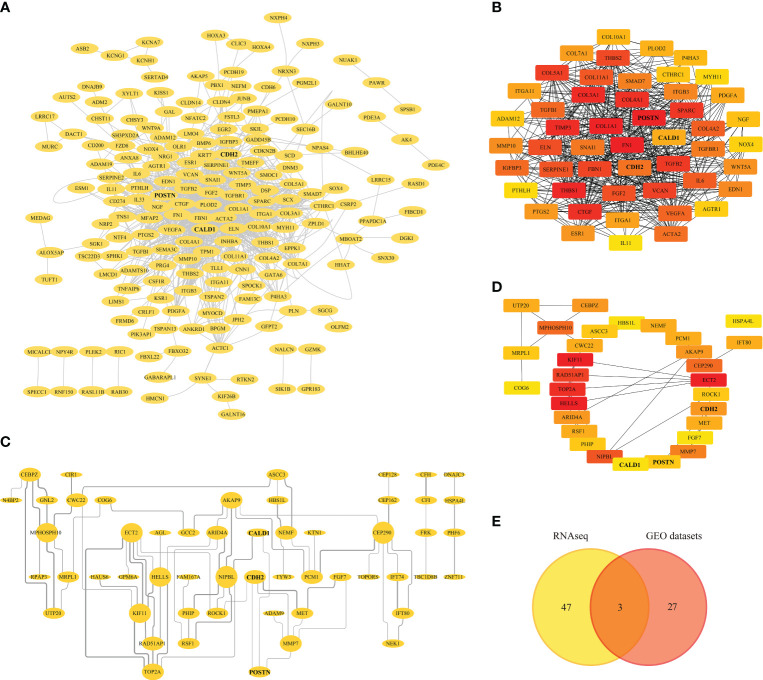
Protein-protein Interaction (PPI) Analysis. **(A)** PPI network of RNAseq-DEGs, nodes and edges represent proteins and protein interactions respectively. **(B)** Identification of genes with the top 50 MCC (Matthews correlation coefficient) scores in the RNAseq-PPI network. **(C)** PPI network of DEGs in GEO datasets. **(D)** Identification of genes with the top 30 MCC scores in the PPI network of GEO datasets. **(E)** Venn diagram depicting the overlapping DEGs, with 3 genes representing the intersection between the RNAseq and GEO datasets.

The 475 RNAseq-DEGs of were introduced into the STRING 11.5 online database, obtaining 473 nodes and 1810 edges. After hiding the disconnected nodes in the network, the network file was imported into Cytoscape for PPI network visualization (**Figure 4A**). By using the cytoHubba plug-in to identify the significant modules of the network, hub genes such as FN1, COL1A1, POSTN, THBS1, COL3A1, TIMP3 with the top 50 MCC (Matthews correlation coefficient) scores were screened (**Figure 4B**; **Supplementary Table S4**).

Similarly, the DEGs from the GEO datasets were subjected to PPI analysis, generating a PPI network with 90 nodes and 75 edges. Disconnected nodes were eliminated, and the network was visualized in Cytoscape (**Figure 4C**). Furthermore, the top 30 hub genes based on their MCC scores were identified, including KIF11, ECT2, HELLS, TOP2A, and RAD521P1 (**Figure 4D**; **Supplementary Table S5**). A Venn diagram (**Figure 4E**) was generated to illustrate the overlapping DEGs with high MCC scores between the RNAseq and GEO datasets. Within this intersection, three hub genes, CALD1, CDH2, and POSTN, were found to be dysregulated and potentially significant in the context of pulmonary fibrosis.

### 3.4 Validation of hub genes expression

**Figure 5:**
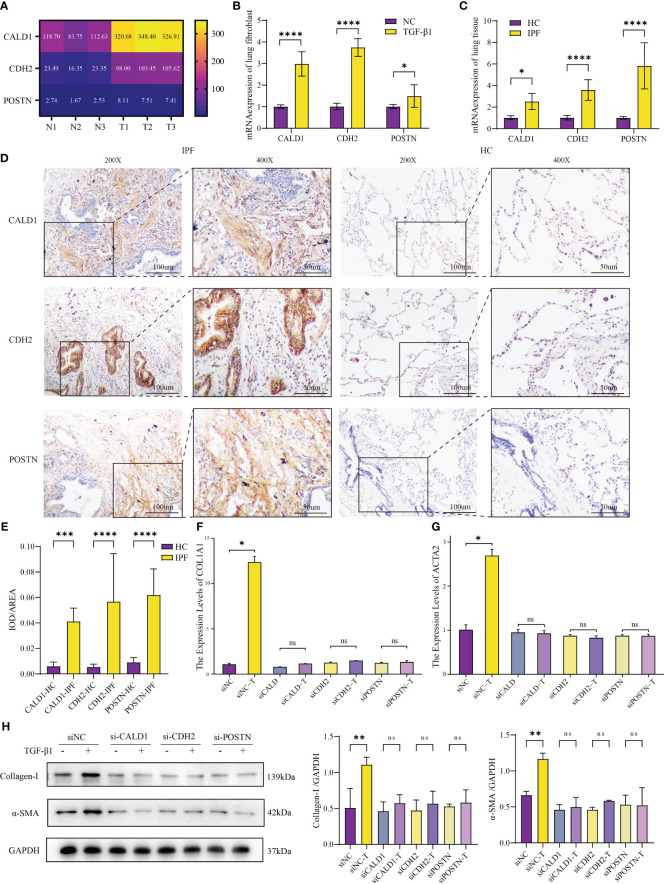
Validation of mRNA expression in IPF fibroblast model and human lung tissue. **(A)** Different mRNA levels of CALD1, CDH2 and POSTN in sequencing results. N1-N3: HLFs isolated from normal lung tissues adjacent to tumors; T1-T3: primary HLFs stimulated with recombinant human TGF-β1 (5 ng/ml) for 24 hours. **(B)** Comparison of CALD1, CDH2, POSTN mRNA expressions in HLFs between TGF-β1 group and normal control (NC) group. n=6 for each group. **(C)** mRNA expressions of CALD1, CDH2, POSTN in healthy control (HC) and IPF patients’ lung tissues measured by RT-qPCR. n=8 for each group. **(D)** Representative immunohistochemistry images and quantification **(E)** of CALD1, CDH2 and POSTN expression in lung fibrosis and normal lung tissues. n=8 for each group. Original magnification 200×, Scale bar 100 μm; Original magnification 400×, Scale bar 50μm. **(F)**. RT-qPCR analysis of COL1A1 gene expression in lung fibroblasts treated with respective siRNA and TGF-β1. **(G)** Quantitative PCR analysis of ACTA2 gene expression in lung fibroblasts treated with respective siRNA and TGF-β1. n=3 for each group. **(H)** Representative western blot images and quantification of Collagen I and α-SMA expression in lung fibroblasts treated with respective siRNA and TGF-β1. *p< 0.05, **p < 0.001, ***p < 0.0001, and ****p < 0.00001, ns, not significant by Welch one-way ANOVA test, Error bars denote SD.

The heatmap (**Figure 5A**) depicts the transcriptome expression values of CALD1, CDH2 and POSTN in the sequencing results. To confirm the bioinformatics results, the relative mRNA expressions of CALD1, CDH2, POSTN in HLFs treated with TGF-β1 (5ng/ml) were evaluated by RT-qPCR in 96 well plates in triplicates. In **Figure 5B**, the mRNA levels of these 3 genes were significantly higher in TGF-β1 group than control group (P<0.05).

To further interrogate the expressions of CALD1, CDH2, and POSTN in fibrotic lung tissue of IPF patients, both normal and fibrotic lung tissues were collected. H&E staining presented representative examples (**Supplementary Figure S1C**). RT-qPCR analysis demonstrated significantly elevated mRNA expression levels of CALD1, CDH2, and POSTN in the IPF lung tissue compared to the normal lung tissue (**Figure 5C**). Notably, the fold increase in POSTN expression observed in the lung tissue of IPF patients exceeds the level verified in the fibroblast model test. In further immunohistochemical analysis (**Figure 5D**), protein expression levels of CALD1, CDH2, and POSTN were significantly elevated at the histological level in IPF patients, a finding substantiated by quantitative results (**Figure 5E**, n=8 for each group) . Normal lung tissue exhibits intact alveolar structures, with only minimal expression of CALD1, CDH2, and POSTN observed in the alveolar septa. In contrast, IPF lung tissue displays loss of alveolar structure, proliferation of interstitial fibrous tissue, a significant increase in CALD1 expression within the cytoplasm of fibroblasts, elevated expression of CDH2 in bronchial epithelial cells and the cytoplasm of fibroblasts, and heightened, brownish-yellow expression of POSTN in proliferating fibrous tissue. The upregulation of these genes in TGF-β1-treated HLFs and IPF lung tissue indicates their potential involvement in fibrotic processes.

### 3.5 Knockdown of CALD1, CDH2, and POSTN attenuates the expression of TGF-β1-induced fibrotic markers in human primary fibroblasts

Upon discovering the elevated expression of CALD1, CDH2, and POSTN in pulmonary fibrosis, we designed two pairs of siRNA for each gene to effectively knockdown their expression in HLFs. Among the tested siRNA duplexes, siCALD1#1, siCDH2#1, and siPOSTN#1 demonstrated the highest knockdown efficiency, leading to less than 30% remaining mRNA levels (**Supplementary Figure S2A**), accompanied by a significant reduction in protein expression as well (**Supplementary Figure S2B**). Consequently, these three siRNA duplexes were selected for further investigations.

To evaluate the functional implications of CALD1, CDH2, and POSTN knockdown, we treated the siRNA-transfected HLFs with TGF-β1 induction. Compared to the control group, the HLFs with suppressed CALD1, CDH2, and POSTN expression did not exhibit an upregulation of collagen I and α-SMA expression, both at the mRNA and protein levels (**Figures 5F, G**). It is noteworthy that even in the absence of TGF-β1 stimulation, the expression of collagen I and α-SMA mRNA and protein levels showed a reduction (**Figure 5H**) when compared to siNC, although the mRNA level did not reach statistical significance. To access the potential impact of knockdown on cell viability, CCK-8 cell proliferation assay results (**Supplementary Figure S2C**) indicated no statistically significant differences in cell vitality among the knockdown groups compared to the control group. Western blot results (**Supplementary Figure S2D**) also suggested no noticeable differences in apoptosis among the knockdown groups. These findings suggest that knockdown of CALD1, CDH2, and POSTN could prevent the induction of collagen I and α-SMA, which are key markers of pulmonary fibrosis, in response to TGF-β1 stimulation.

### 3.6 Co-expression analysis of hub genes

**Figure 6:**
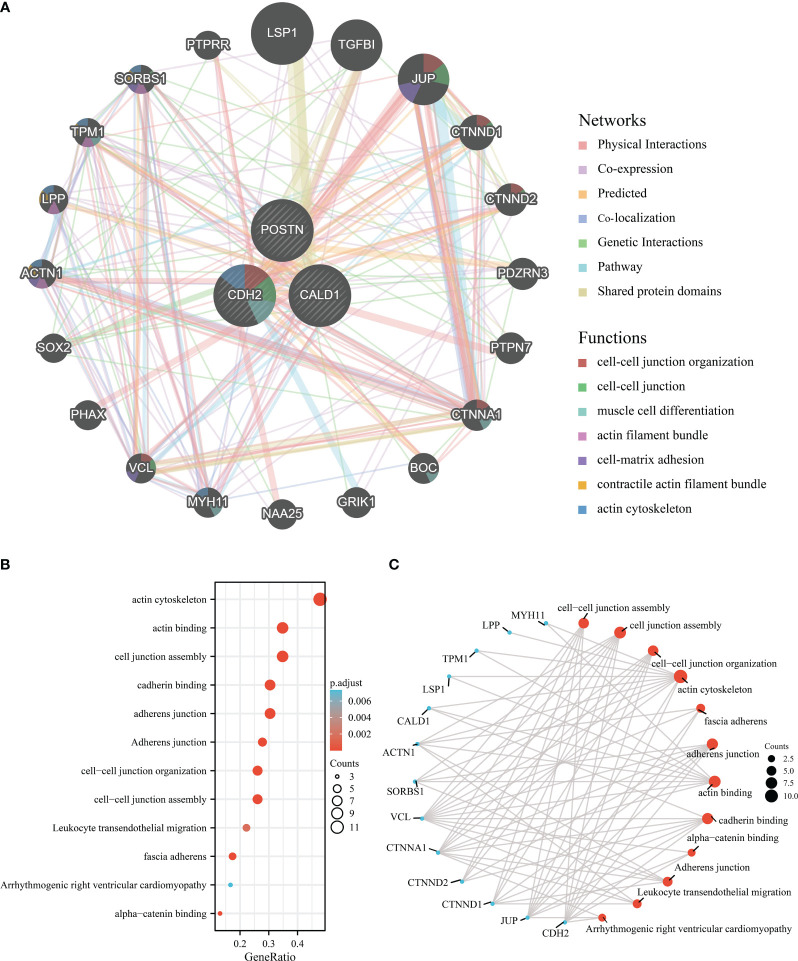
Interaction analysis of the feature genes. **(A)** Gene-gene interaction network for DEGs analyzed using the GeneMANIA database. The 20 most frequently altered neighboring genes are shown as nodes, with node color representing gene function. **(B)** Enrichment analysis of GO and KEGG pathways related to the 23 genes mentioned above. **(C)** Functional network of enrichment analysis constructed to visualize connections and associations between different functional categories.

The co-expression network of hub genes was obtained by GeneMANIA (**Figure 6A**) with automatically weighting method. The gene-gene interaction network consisted of 3 central genes and 20 peripheral predicted genes including LSP1, TGFBI, JUP, etc. The potential functions of the predicted genes and possible downstream pathways were also predicted. According to the prediction results, peripheral predicted genes of hub genes may be related to functions such as cell-cell junction organization, actin cytoskeleton.

Subsequently, we perform the 3 central genes and 20 peripheral predicted genes into enrichment analysis, and the Gene Ontology analysis results (**Figures 6B, C**) revealed significant enrichments in various functions, including actin cytoskeleton, actin binding, cell junction assembly, cadherin binding, adherens junction, cell-cell junction organization, cell-cell junction assembly, leukocyte transendothelial migration, fascia adherens, arrhythmogenic right ventricular cardiomyopathy, and alpha-catenin binding.

Overall, these findings highlight the potential functional roles of the hub genes and their associated predicted genes in actin cytoskeleton regulation, cell-cell junction organization, and related processes.

## 4 Discussion

IPF is a progressive fibrotic interstitial lung disease with poor prognosis and limited therapies, usually leading to irreversible scarring of lung tissues which perpetuates the disease. Though an increasing number of studies have been conducted in recent years (2), understanding of the underlying pathogenesis is urgently needed for guiding more effective therapies.

TGF-β1 is crucial in IPF, driving fibrotic processes by stimulating collagen production and promoting myofibroblast differentiation. It also induces inflammation and fibroblast activation, exacerbating the fibrotic response in IPF (19). In this study, we performed RNA sequencing on human primary lung fibroblasts treated with recombinant human TGF-β1 for 24 hours, along with a control group. Each group consisted of 3 biological replicates. Analysis of the sequencing data revealed 475 differentially expressed genes (DEGs). Functional analysis using GO annotation and KEGG enrichment showed that these DEGs are mainly associated with cellular response to transforming growth factor beta stimulus, tissue development (connective tissue, cartilage, and striated muscle), and components of the extracellular matrix (collagen-containing extracellular matrix, basement membrane, and endoplasmic reticulum lumen). These DEGs also exhibited activities such as collagen binding, receptor ligand activity, and signaling receptor activator activity. Pathway analysis identified their involvement in various pathways, including the AGE-RAGE signaling pathway in diabetic complications, ECM-receptor interaction, Amoebiasis, PI3K-Akt signaling pathway, and Malaria.

Furthermore, we screened a total of 29 IPF patients’ lung samples and 14 normal lung samples from GEO’s GSE101286 and GSE110147 datasets, and 94 DEGs were identified by GEO2R and Venn analysis. GO annotation and KEGG enrichment analysis of these genes through cluster Profiler revealed that DEGs were mainly involved in cellular components and function, such as membrane docking, organelle localization by membrane tethering, cilium assembly, cilium organization, microtubule organizing center part, centriole, centriole satellite, and presynaptic active zone membrane. Membrane docking represents the initial attachment of a tight membrane or protein to a target membrane, and organelle membrane interacts with another membrane via molecular tethers, which physically bridge and attach the two membranes (20). The pathologic features of IPF include stromal deposition, basement membrane disruption, and mesenchymal amplification, in which the membrane-to-membrane connection is inextricably linked to fibroblast proliferation (21). Membrane receptors at the cell surface transduce external signals into cellular responses and are also involved in controlling cellular signaling (22). The primary cilium assembles from the basal body derived from the mother centriole, the transition zone, and the axoneme consisting of nine doublet microtubules (23). The recent study (24) proposed the primary cilium for the initiation of the transition and sustained activation of myofibroblasts, which simultaneously plays a critical role in driving IPF (3). Thus, exploring the role of cilium in IPF may be a new line of inquiry.

In addition, we constructed the PPI network of DEGs by STRING and Cytoscape, and obtained KIF11, ECT2, HELLS, TOP2A, RAD521P1 with MCC scores above 20, reflecting their centrality in the network. Except for HELLS which encodes a lymphoid-specific helix, the remaining four genes are involved in physical interactions, such as chromosome aggregation, ATPase activity concerning cell proliferation (25–27). Since CALD1, CDH2 and POSTN were screened by taking intersection with sequencing data, RT-qPCR experiments were performed to validate the high-level mRNA expressions in the TGF-β1-induced primary fibroblast model, as well as lung tissue of IPF patients. Additionally, immunohistochemistry experiments were performed to assess their protein expressions in lung tissue. Furthermore, to assess the functional impact of CALD1, CDH2, and POSTN, knockdown experiments were performed individually for each gene by siRNA. These findings demonstrated that, under TGF-β1 stimulation, the knockdown of these genes led to a significant decrease in the expression of fibrotic markers, highlighting the potential therapeutic relevance of targeting CALD1, CDH2, and POSTN for attenuating fibrotic processes in pulmonary fibrosis.

CALD1 (Caldesmon 1) encodes a cytoskeleton-associated protein CaD (caldesmon) that regulates smooth muscle and nonmuscle contraction (28). CaD is involved in the epithelial-mesenchymal transition (EMT) in cell migration, invasion and proliferation by interacting with actin and myosin and stabilizing actin filaments (29). The critical mechanism in the pathogenesis of IPF is that the TGF-β signaling activates the differentiation and proliferation of fibroblasts into myofibroblasts which expresses α-smooth muscle actin (α-SMA) and promotes EMT (3, 4). Some researchers (30) have found that overexpression of caldesmon potentiated TGF-β1 induced expression of α-SMA during EMT in NMuMG cells, which indicated that CALD plays an important role in mediating fibrosis. Most notably, there are few reports about accurate relationship between CALD1 and IPF so far, and our investigation uncovered that the high expression of CALD1 is related to IPF, suggesting that CALD1 may be involved in the pathogenesis. Typically, our immunohistochemistry results showed that high expression of CALD1 was mainly in foci of proliferating fibroblasts surrounding small airways. Further investigation into whether this molecular operates in IPF will be of great importance and interest.

CDH2 (Cadherin 2, N-cadherin) is a member of the cadherin family, a calcium-dependent single-chain transmembrane glycoprotein that mediates cell-cell adhesion (31, 32), which is expressed in nervous, fibrous, and musculoskeletal tissues (33). Considerable researches have been devoted to investigate the role of N-cadherin in invasion and metastasis of carcinoma induced by EMT, which characterized by the upregulation of N-cadherin followed by the downregulation of E-cadherin (33, 34). N-cadherin junction stabilizes the fibroblast growth factor receptor (FGFR) to reinforce angiogenesis and cell proliferation and migration via activating MAPK/ERK signaling pathway and PI3K-I/Akt pathway, while E-cadherin enhances cell-cell adhesion via suppressing activation of Wnt/β-catenin and PI3K pathway (34). Importantly, several findings have revealed that EMT causally contributes to pulmonary fibrosis, which was triggered by multiple pathways and molecules (35–38). Despite these cues, the exploration of N-cadherin in pulmonary fibrosis has often been limited to its role as a molecular marker for EMT, with limited investigation into its molecular mechanisms in fibrosis. In a recent study (39), the focus shifted to pro-N-Cadherin (PNC), revealing abnormal PNC localization in the bronchiolar epithelium of fibrotic lungs, closely aligning with our experimental results. This aberrant localization might be attributed to the cross-reactivity of the antibody we used with PNC, as our detection primarily centered on mature N-Cadherin. The study utilized antibodies that did not cross-react between PNC and N-cadherin. Their results indicated abnormal PNC localization in fibrotic tissues, including the heart, lungs, and liver. Furthermore, PNC was found to be highly expressed on the surface of myofibroblasts isolated from fibrotic tissues compared to normal tissues. However, the mechanisms of PNC and N-cadherin in pulmonary fibrosis require further investigation, as many aspects remain unclear.

POSTN (Periostin), initially termed osteoblast-specific factor 2, encodes an extracellular matrix protein and promotes cell adhesion (40). Periostin is secreted by fibroblasts, epithelial and endothelial cells, which drives chronic inflammation and fibrosis in various tissues (40, 41). In respiratory diseases, periostin is recognized to be contributed in the development of asthma and IPF (42). Further investigations illustrated that periostin secreted by airway epithelial cells induces activation of TGF-β and up-regulation of collagen I, which lead to differentiation of fibroblasts to myofibroblasts as well as collagen fibrillogenesis and cross-linking (43). Consistently, several lines of evidence indicated that periostin is highly expressed in the serum and lung tissue of IPF patients (44, 45). This observation is further supported by the findings from several immunohistochemical studies, which consistently demonstrate the specific localization of periostin to regions of active fibrosis, including fibrotic foci and the subepithelial and subendothelial regions (41, 45–47). Recent research shows that periostin enhances the TGF-β/Smad2/3 signaling pathway via integrin α_V_β_3_/α_V_β_5_ in lung fibroblasts, contributing to pulmonary fibrosis development. Periostin also acts as a downstream effector of TGF-β in these fibroblasts, implying that the interaction between TGF-β and periostin may amplify TGF-β signaling through periostin (47). Our experimental validation indicates that the increase in POSTN expression observed in lung tissues from IPF patients significantly surpasses the levels validated in the fibroblast model tests. We hypothesize that this difference may be linked to post-transcriptional regulation, recognizing the inherent limitations in the cell model. In a study (48) involving hypoxia-induced pulmonary hypertension (HPH), miR205, miR-20a-5p, and miR-541 were predicted to target POSTN, and these microRNAs might compete with several lncRNAs and circRNAs for binding to POSTN. Whether POSTN exhibits certain post-transcriptional regulatory mechanisms in pulmonary fibrosis remains to be investigated. Furthermore, a study (49) revealed that periostin originating from activated fibroblasts in the IPF lung significantly contributes to the proliferation of non-small cell lung cancer (NSCLC), and inhibiting the interaction between periostin and its receptors can effectively mitigate the aggressive behavior induced by IPF in NSCLC. Additionally, it has been reported that treatment of antibodies (OC-20) against periostin effectively improved the fibrotic progression and prognosis in mouse models of bleomycin (BLM)-induced pulmonary fibrosis (45). Another research evaluated small interfering RNA or antisense oligonucleotide against periostin can inhibit lung fibrosis efficently by direct administration into the lung by intranasal route (50). In summary, there is a clear association between POSTN and IPF, and inhibiting POSTN shows promise as a treatment for lung fibrosis.

Moreover, the co-expression network of CALD1, CDH2, and POSTN was examined by gene MANIA. Significantly, TGF-β1 had vital connections with CALD1, CDH2, and POSTN, and also linked 20 peripheral predicted genes like SORBS1, CTNND2, ACTN1, SOX2, which worth further exploration. Enrichment analysis of these 23 genes revealed significant enrichments in various functions, including actin regulation, cell junction organization, cadherin binding, adherens junctions, leukocyte transendothelial migration, and fascia adherens. These functions are closely related to cellular structure and adhesion and may contribute to the structural alterations observed in the alveoli and interstitium, as well as the abnormal cell-cell interactions associated with pulmonary fibrosis. During the development of IPF, these functions likely play a crucial role in cell adhesion, maintaining the integrity of lung tissue structure, and influencing the migration and actions of inflammatory cells (51).

In summary, our study uncovered the significant involvement of CALD1, CDH2, and POSTN in the process of fibrogenesis and shed light on the potential mechanism underlying the aberrant expression of these genes in IPF patients. We observed elevated expression of CALD1, CDH2, and POSTN in both a primary fibroblast model and lung tissues of IPF patients. Furthermore, knockdown of CALD1, CDH2, and POSTN led to a reduction in TGF-β1-induced fibrotic markers, suggesting their potential roles in the development of pulmonary fibrosis. These findings highlight the potential contribution of CALD1, CDH2, and POSTN to the pathogenesis of pulmonary fibrosis.

## Data availability statement

The data presented in the study are deposited in the NCBI SRA repository, accession number PRJNA842213.

## Ethics statement

The studies involving humans were approved by Ethics Committee of Nanjing Drum Tower Hospital in accordance with the Declaration of Helsinki (1989) (NO.2016-160-01). The studies were conducted in accordance with the local legislation and institutional requirements. The participants provided their written informed consent to participate in this study.

## Author contributions

SW: Conceptualization, Validation, Visualization, Writing – original draft, Methodology, Project administration, Software, Writing – review & editing. ML: Data curation, Formal analysis, Supervision, Writing – review & editing. MZ: Investigation, Resources, Validation, Writing – review & editing. XuY: Data curation, Project administration, Writing – review & editing. HG: Investigation, Visualization, Writing – review & editing. CJ: Data curation, Writing – review & editing. HZ: Software, Visualization, Writing – review & editing. XiY: Resources, Writing – review & editing. QL: Resources, Validation, Writing – review & editing. XH: Conceptualization, Writing – review & editing, Writing – original draft. MC: Funding acquisition, Project administration, Writing – review & editing, Writing – original draft.

## Supplementary material

Supplementary Figure 1 Interaction analysis of the feature genes. **(A)** Sirius red staining images visualize collagen fibers in the control group and TGF-β1 stimulated group of primary human lung fibroblasts (HLFs). The images were captured under original magnification ×200. **(B)** Western blot analysis of Collagen I and α-SMA expression in the cell model following TGF-β1 induction. **(C)** Representative H&E staining images of healthy control and IPF patients’ lung tissue at ×200 magnification.

Supplementary Figure 2 Validation of Knockdown Efficacy and Assessment of Cellular Responses. **(A)** Validation of CALD1, CDH2, and POSTN knockdown by RT-qPCR. **(B)** Validation of CALD1, CDH2, and POSTN knockdown by western blot. Each gene was knocked down using two different siRNAs, and the most effective knockdown was selected for subsequent experiments. *p< 0.05, **p < 0.001, ***p < 0.0001, and ****p < 0.00001., ns, not significant by Welch one-way ANOVA test, Error bars denote SD. **(C)** Cell proliferation ability were assessed by Cell Counting Kit-8 (CCK8) after siRNAs knockdown. p >0.05. **(D)**.Western blot analysis of Bcl2 and Bax expression to access cellular apotosis, and the Bax/Bcl2 ratio was calculated.

Supplementary Table 1 Primer used to amplify the human transcripts during RT-qPCR.

Supplementary Table 2 Sequences of siRNAs.

Supplementary Table 3 Antibodies for western blot and IHC.

Supplementary Table 4 Top 50 hub genes in RNA-seq PPI network edges ranked by MCC method.

Supplementary Table 5 Top 30 hub genes in PPI network edges of GEO datasets ranked by MCC method.
