# Supplementary figures and images for: The gene expression of CALD1, CDH2, and POSTN in fibroblast are related to idiopathic pulmonary fibrosis (part 1 of 2)
Source: Front Immunol. 2024 Feb 2;15:1275064. doi: 10.3389/fimmu.2024.1275064 (PMC10869495; doi:10.3389/fimmu.2024.1275064)

A

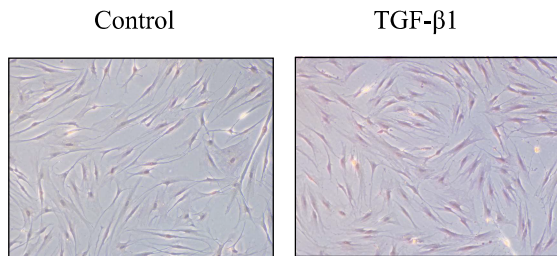

B

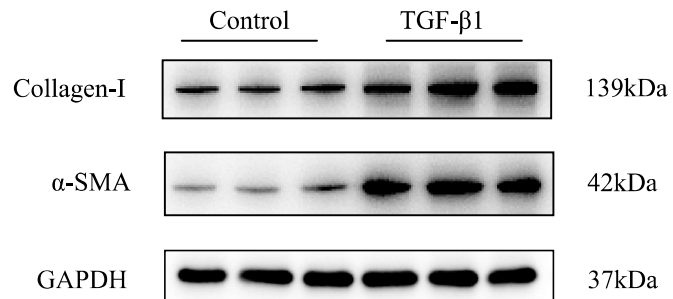

C

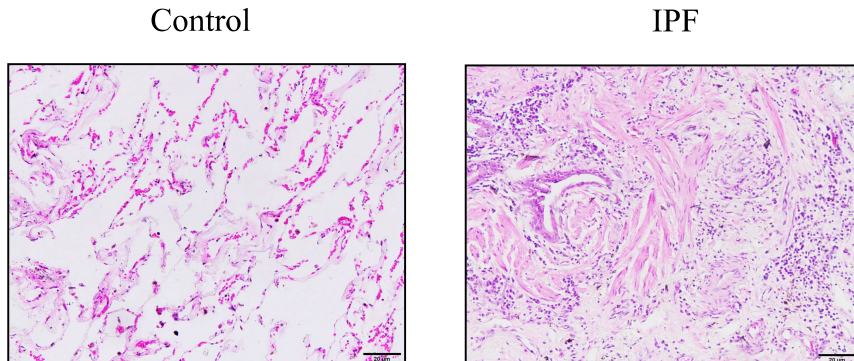

Supplement: Supplementary Figure 1 — Interaction analysis of the feature genes. (A) Sirius red staining images visualize collagen fibers in the control group and TGF-β1 stimulated group of primary human lung fibroblasts (HLFs). The images were captured under original magnification ×200. (B) Western blot analysis of Collagen I and α-SMA expression in the cell model following TGF-β1 induction. (C) Representative H&E staining images of healthy control and IPF patients’ lung tissue at ×200 magnification. [file Image_1.pdf]

A

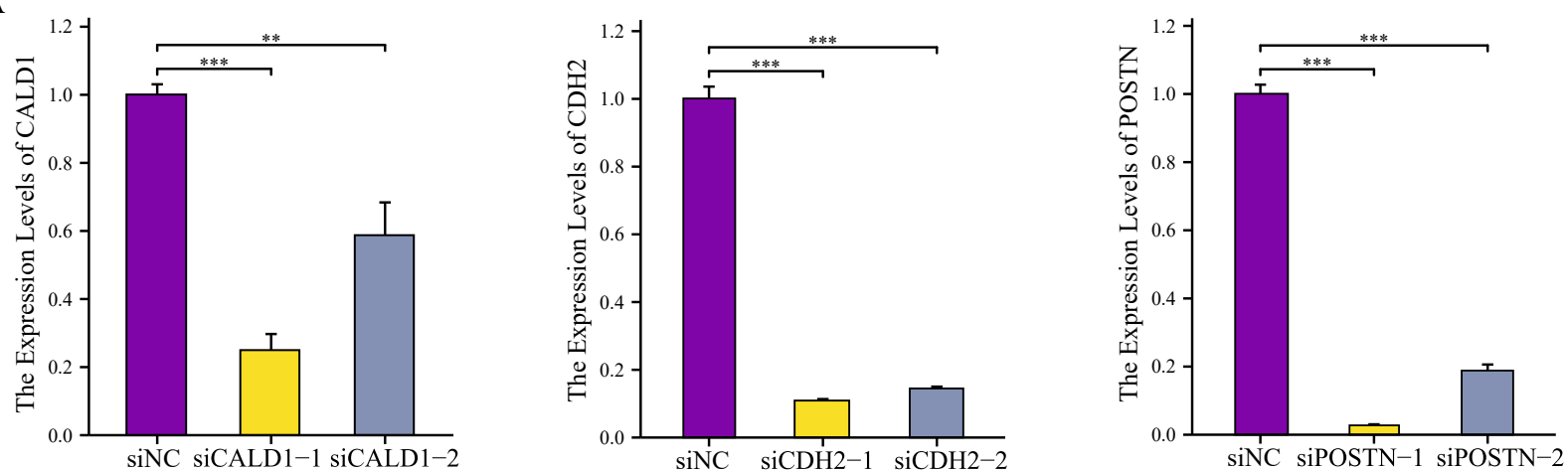

B

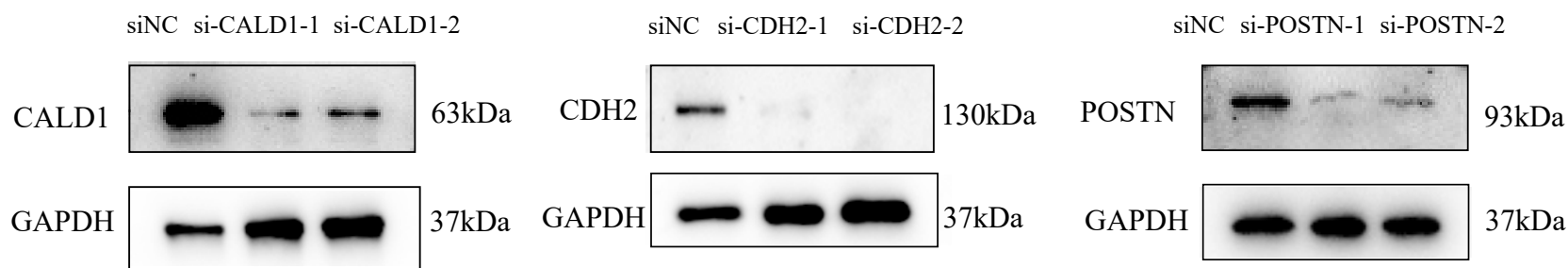

C

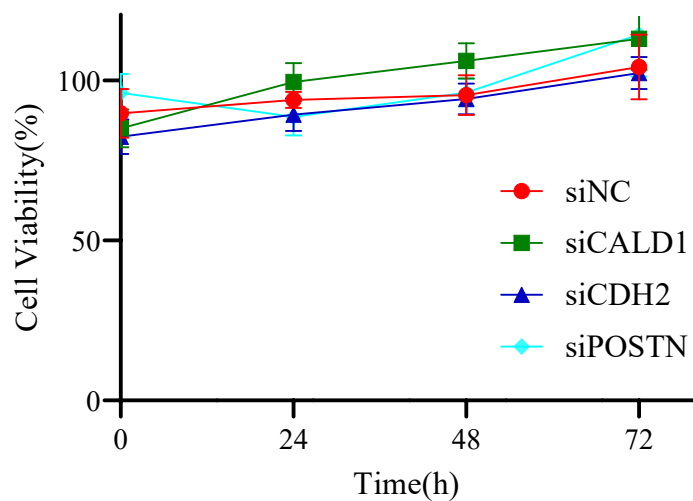

D

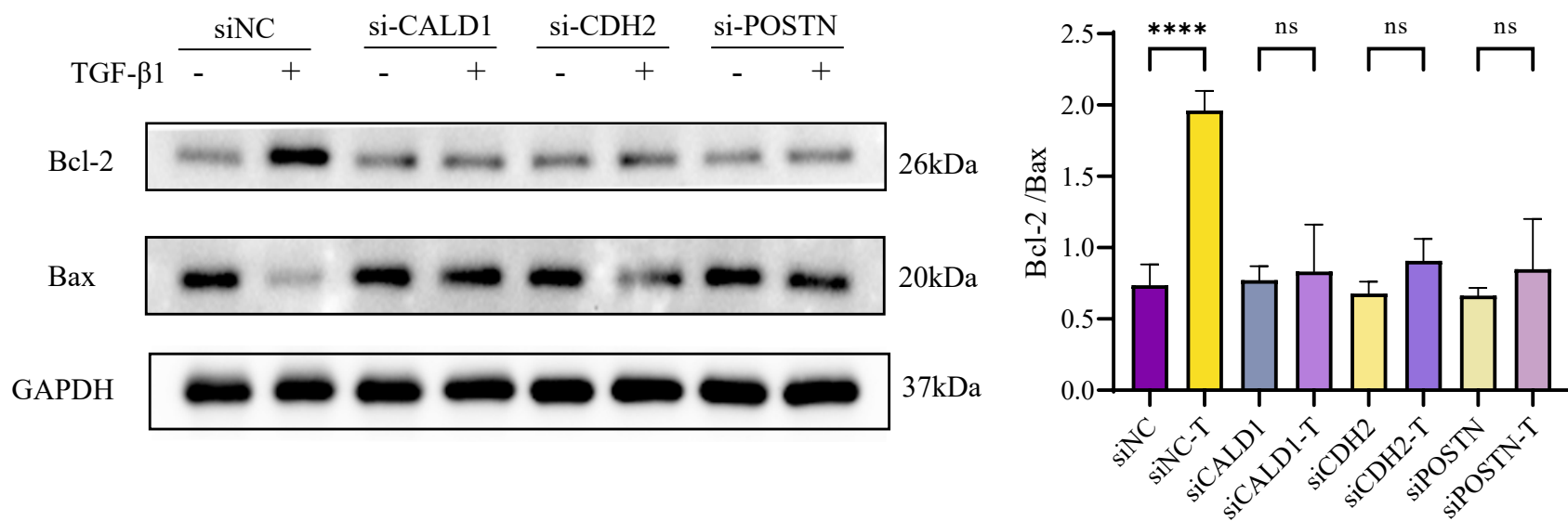

Supplement: Supplementary Figure 2 — Validation of Knockdown Efficacy and Assessment of Cellular Responses. (A) Validation of CALD1, CDH2, and POSTN knockdown by RT-qPCR. (B) Validation of CALD1, CDH2, and POSTN knockdown by western blot. Each gene was knocked down using two different siRNAs, and the most effective knockdown was selected for subsequent experiments. *p< 0.05, **p < 0.001, ***p < 0.0001, and ****p < 0.00001., ns, not significant by Welch one-way ANOVA test, Error bars denote SD. (C) Cell proliferation ability were assessed by Cell Counting Kit-8 (CCK8) after siRNAs knockdown. p >0.05. (D).Western blot analysis of Bcl2 and Bax expression to access cellular apotosis, and the Bax/Bcl2 ratio was calculated. [file Image_2.pdf]

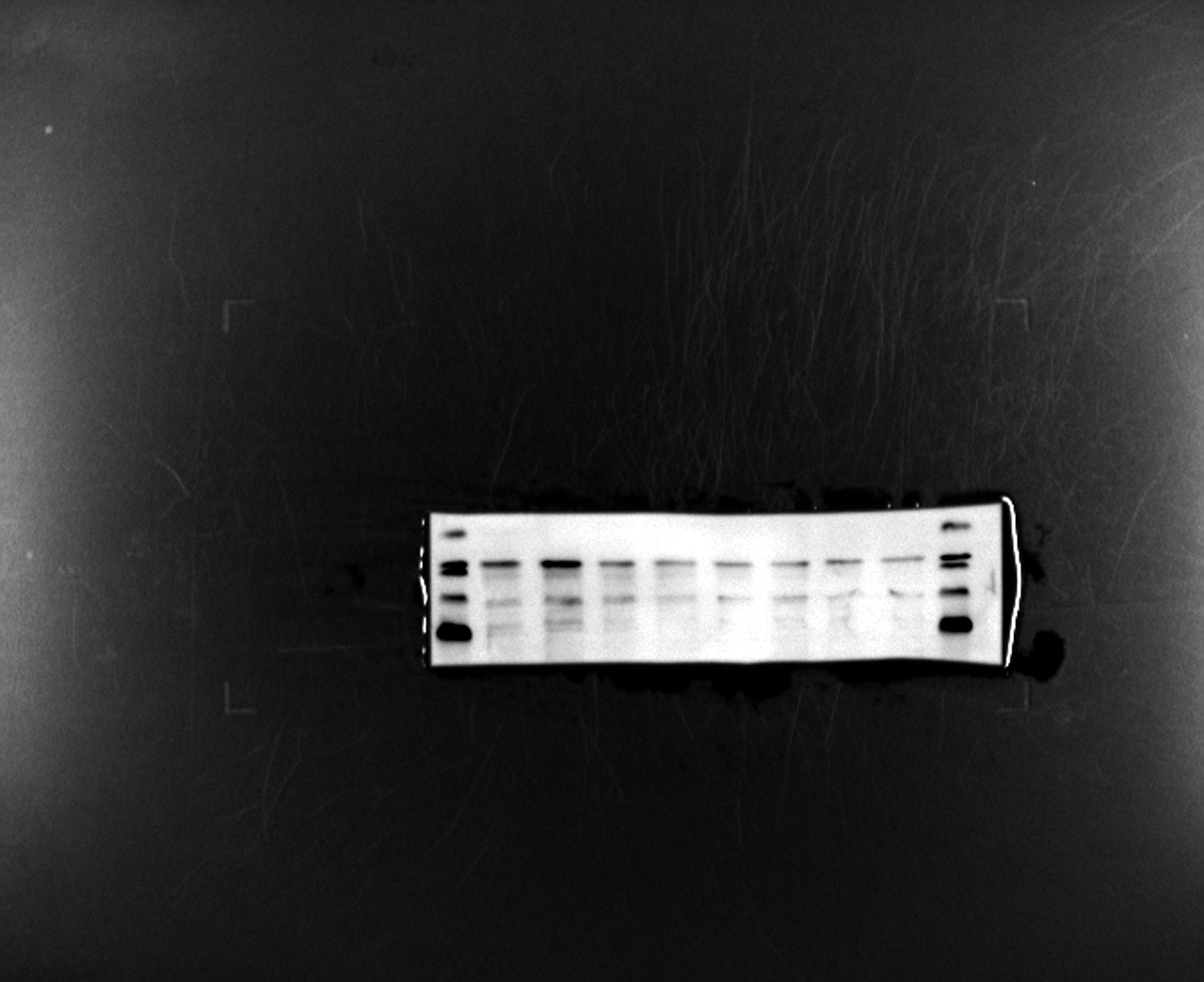

Supplement: Supplementary file 7 [file DataSheet_3.zip › fig5H-col1-1-0.Tif]

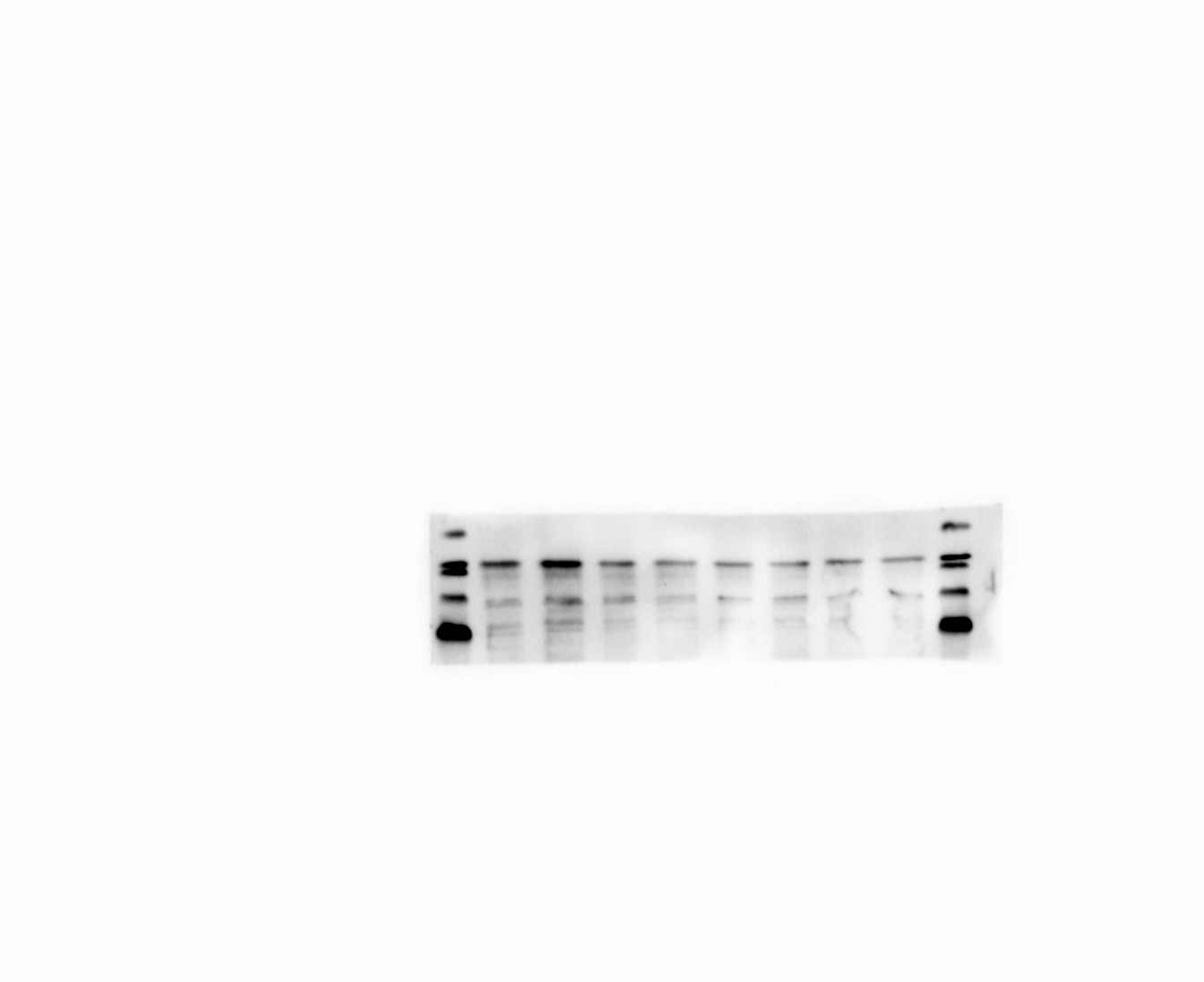

Supplement: Supplementary file 7 [file DataSheet_3.zip › fig5H-col1-1.Tif]

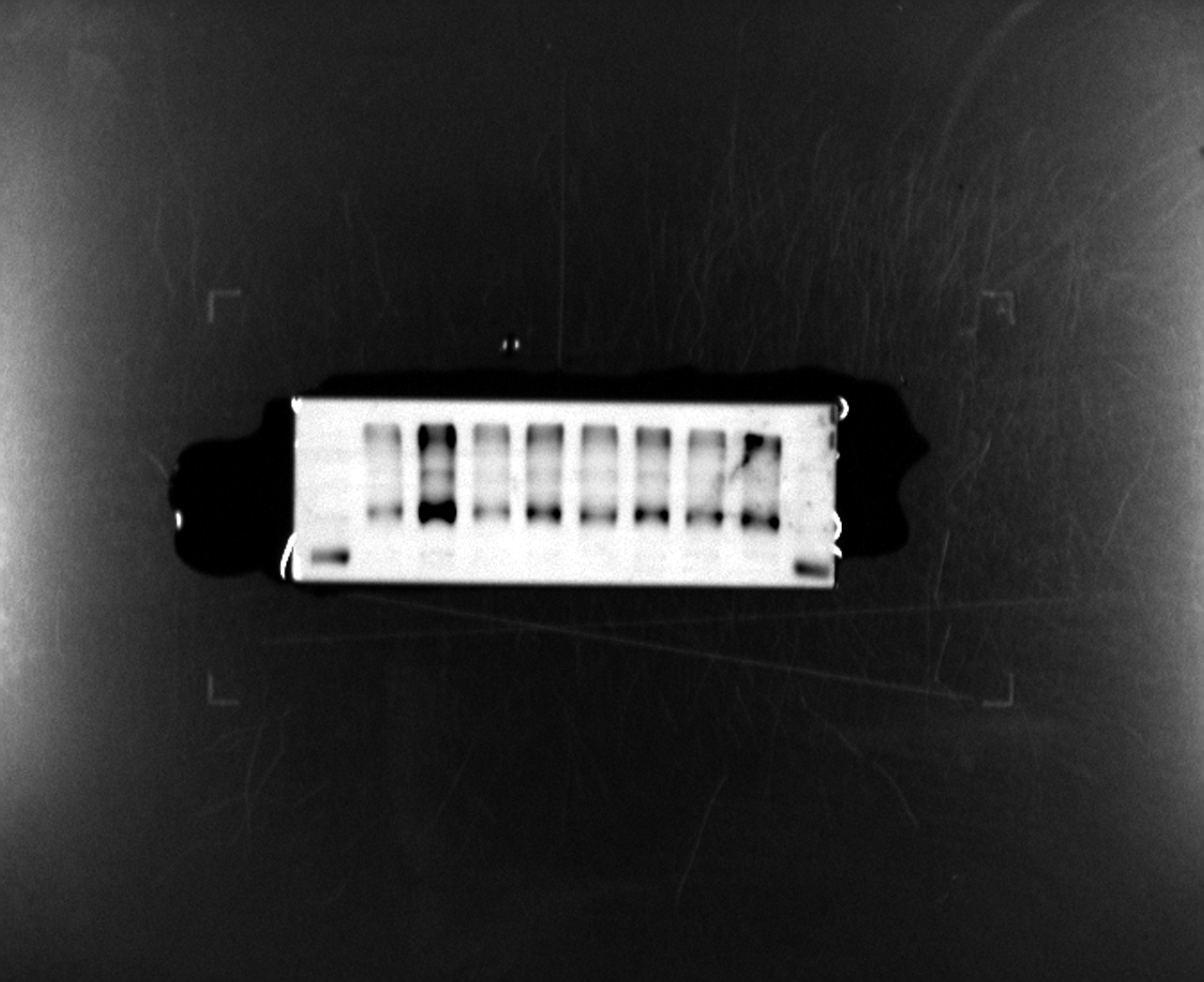

Supplement: Supplementary file 7 [file DataSheet_3.zip › fig5H-col1-2-0.Tif]

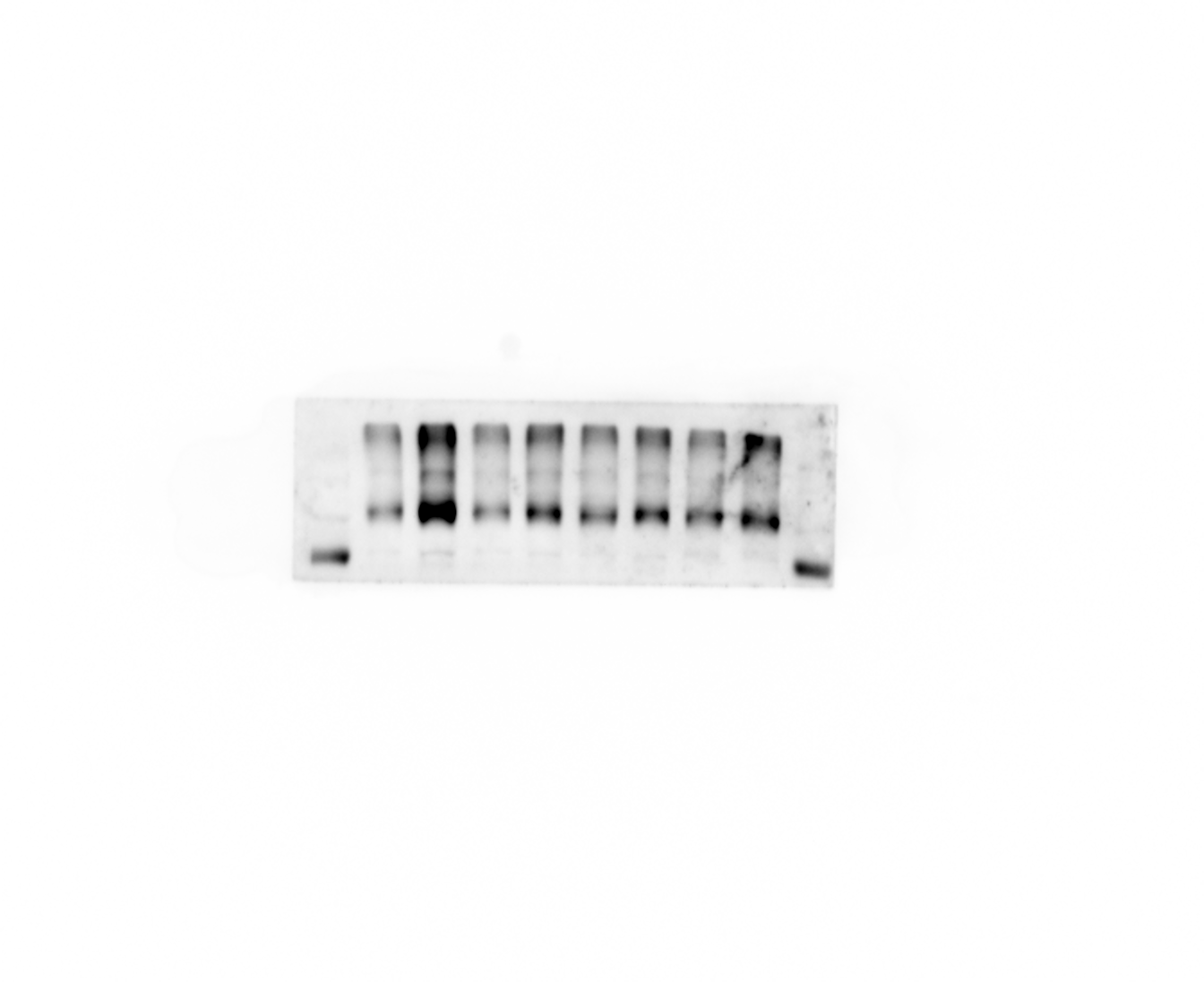

Supplement: Supplementary file 7 [file DataSheet_3.zip › fig5H-col1-2.Tif]

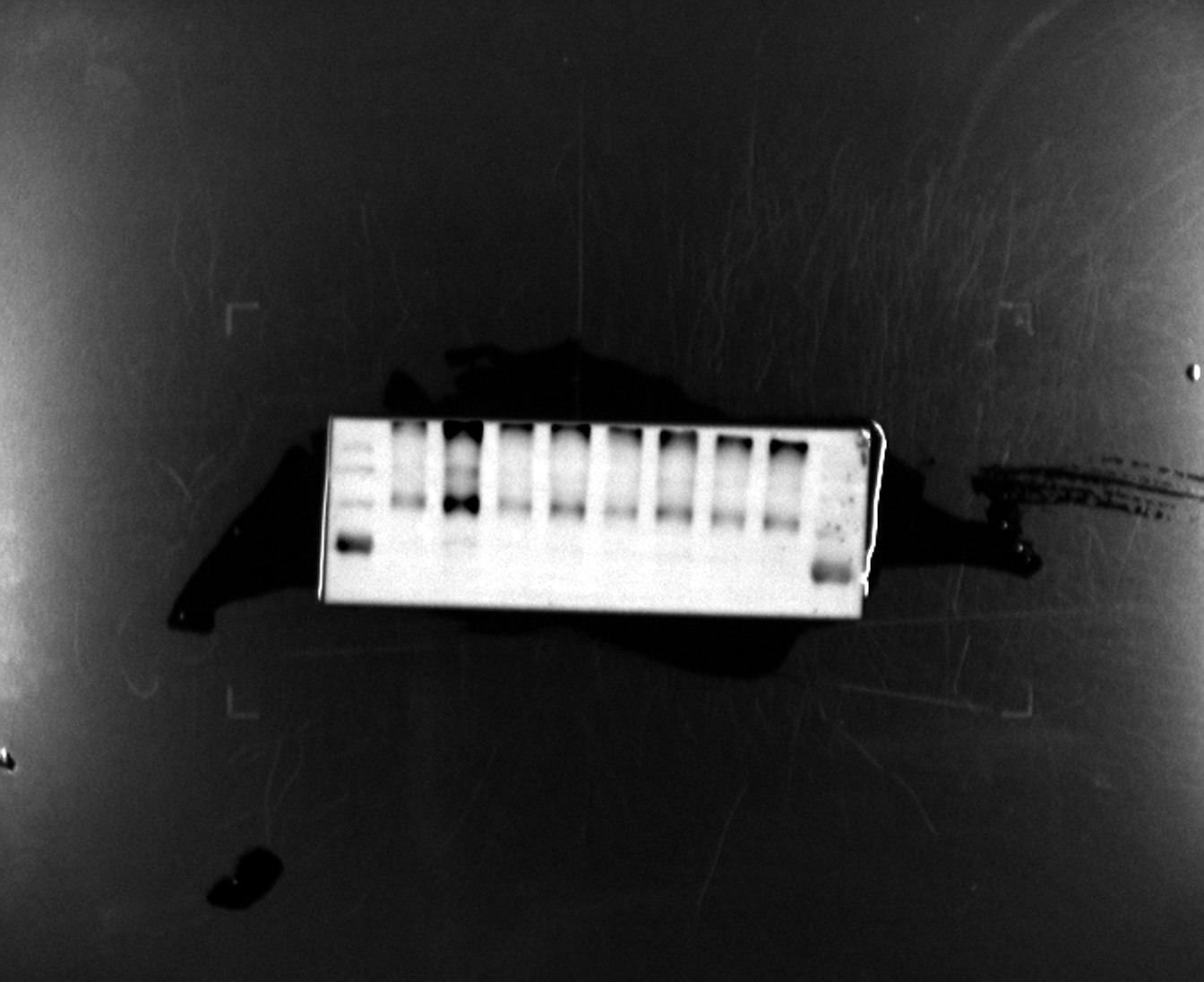

Supplement: Supplementary file 7 [file DataSheet_3.zip › fig5H-col1-3-0.Tif]

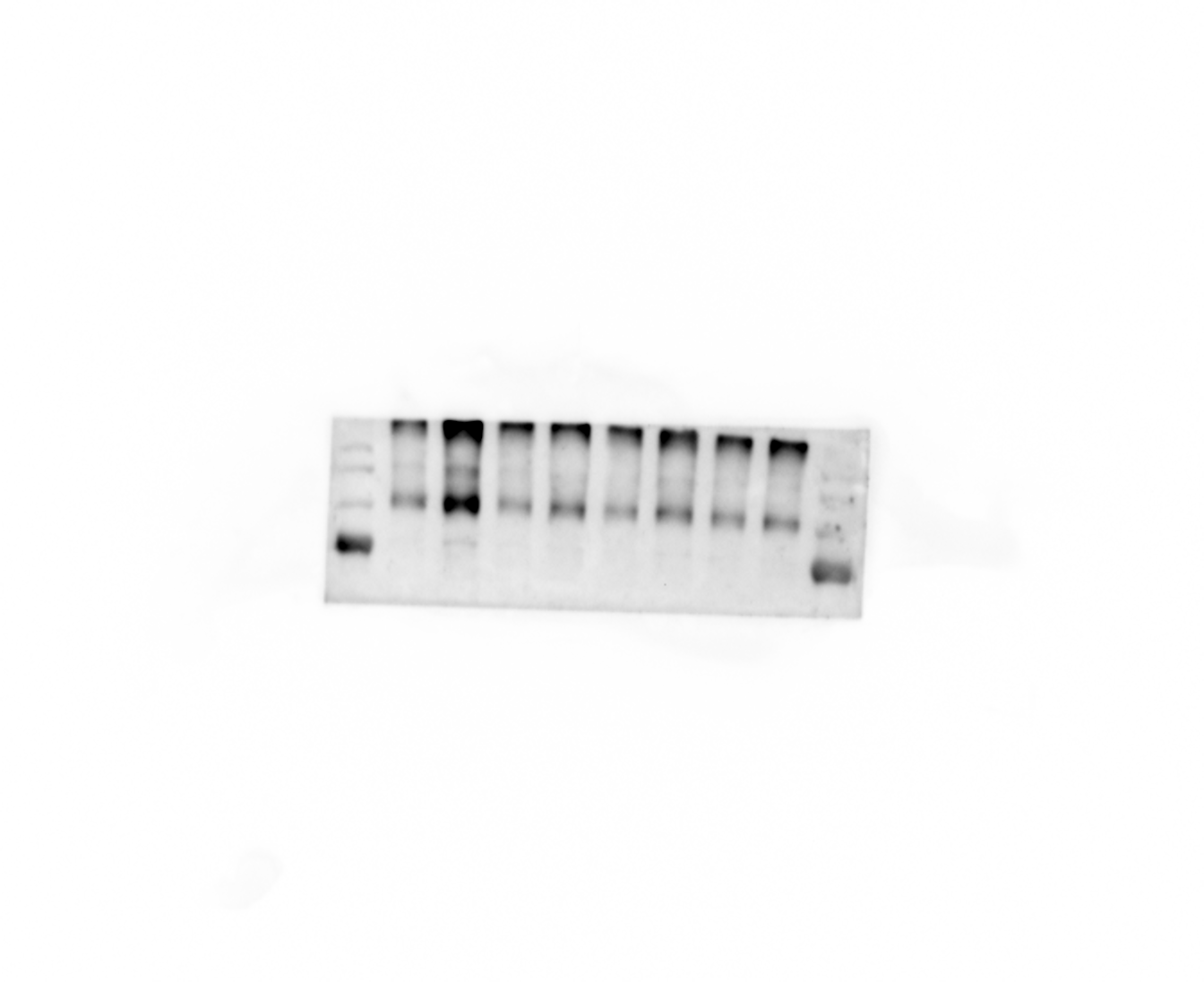

Supplement: Supplementary file 7 [file DataSheet_3.zip › fig5H-col1-3.Tif]

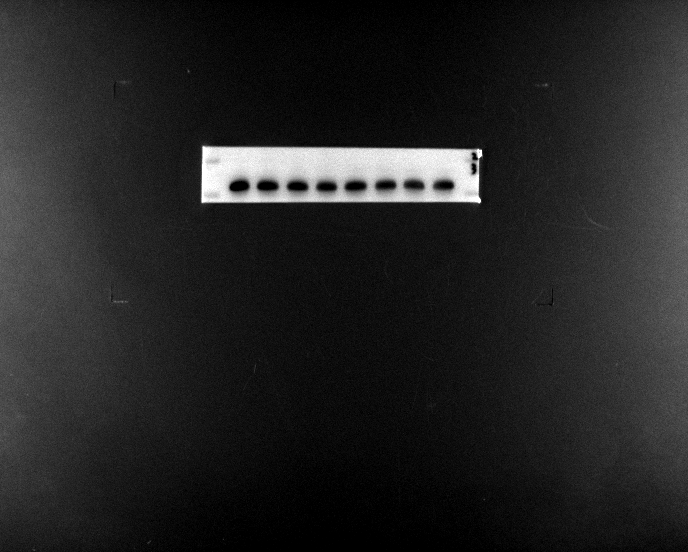

Supplement: Supplementary file 7 [file DataSheet_3.zip › fig5H-gapdh-1-0.Tif]

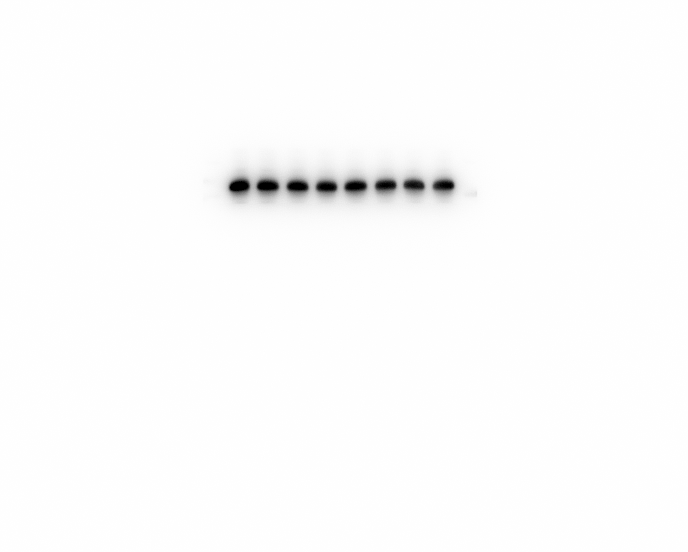

Supplement: Supplementary file 7 [file DataSheet_3.zip › fig5H-gapdh-1.Tif]

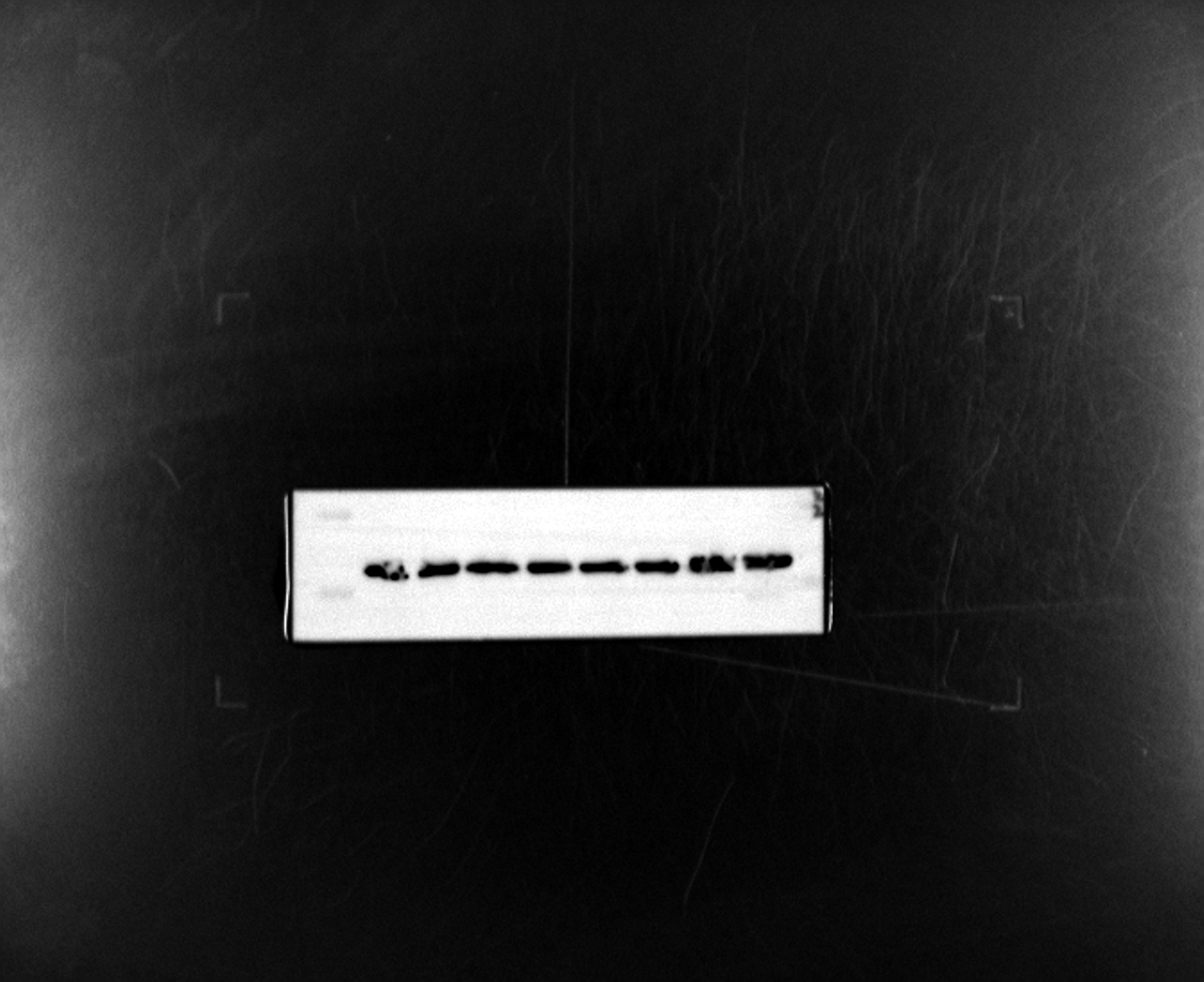

Supplement: Supplementary file 7 [file DataSheet_3.zip › fig5H-gapdh-2-0.Tif]

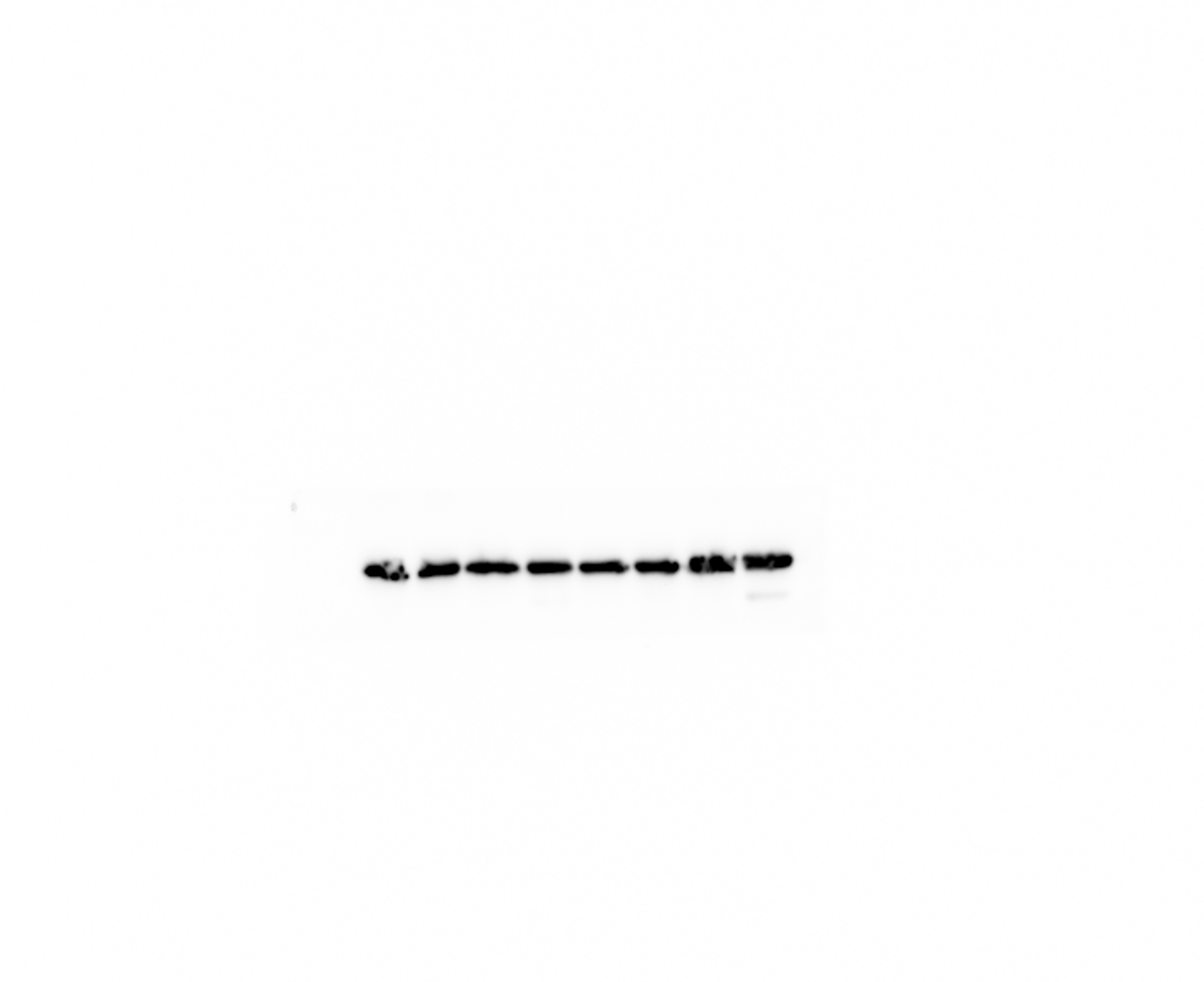

Supplement: Supplementary file 7 [file DataSheet_3.zip › fig5H-gapdh-2.Tif]

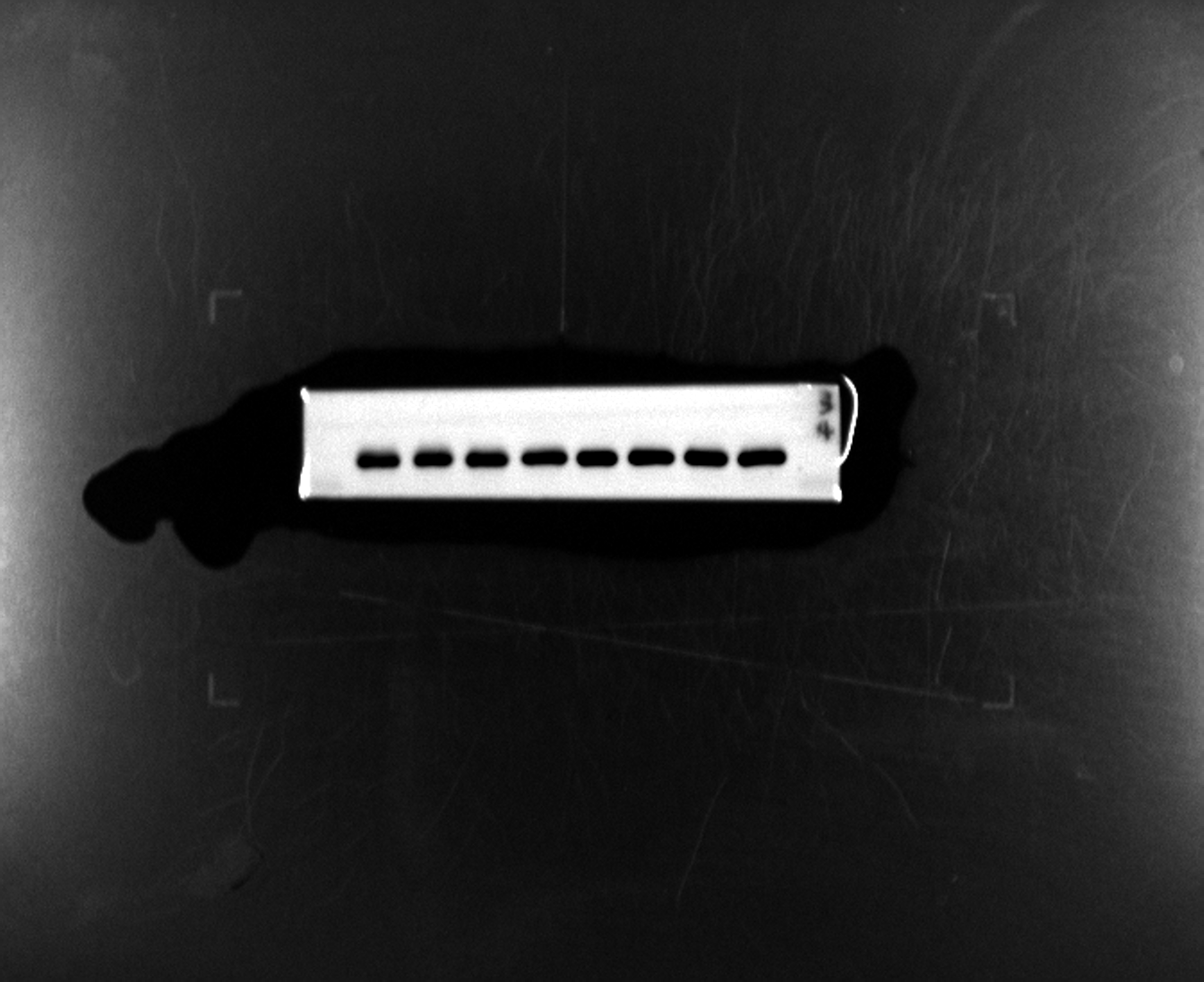

Supplement: Supplementary file 7 [file DataSheet_3.zip › fig5H-gapdh-3-0.Tif]

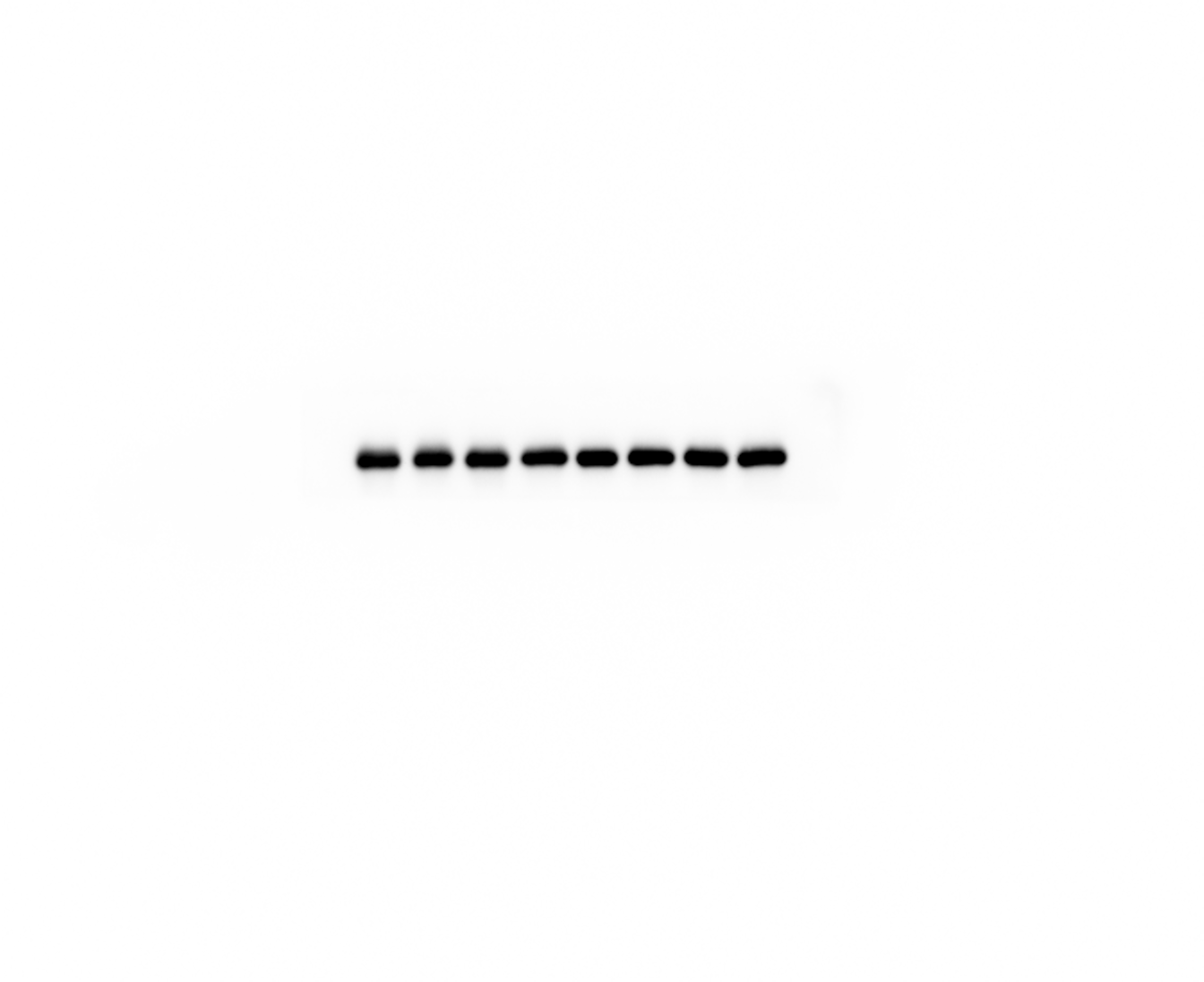

Supplement: Supplementary file 7 [file DataSheet_3.zip › fig5H-gapdh-3.Tif]

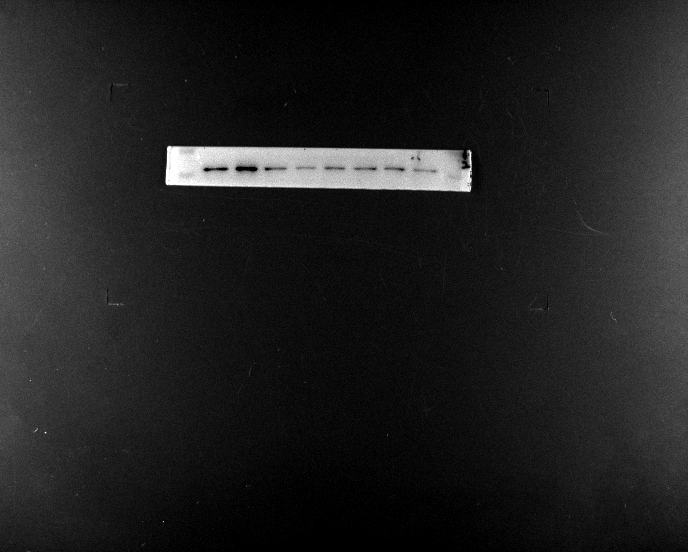

Supplement: Supplementary file 7 [file DataSheet_3.zip › fig5H-sma-1-0.Tif]

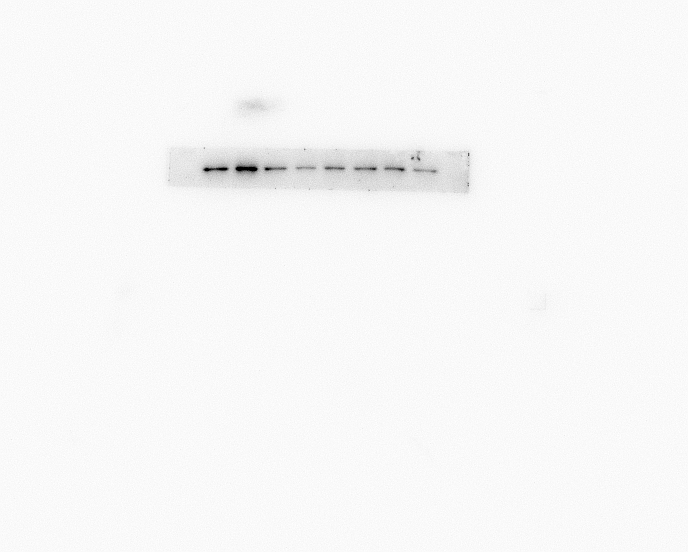

Supplement: Supplementary file 7 [file DataSheet_3.zip › fig5H-sma-1.Tif]

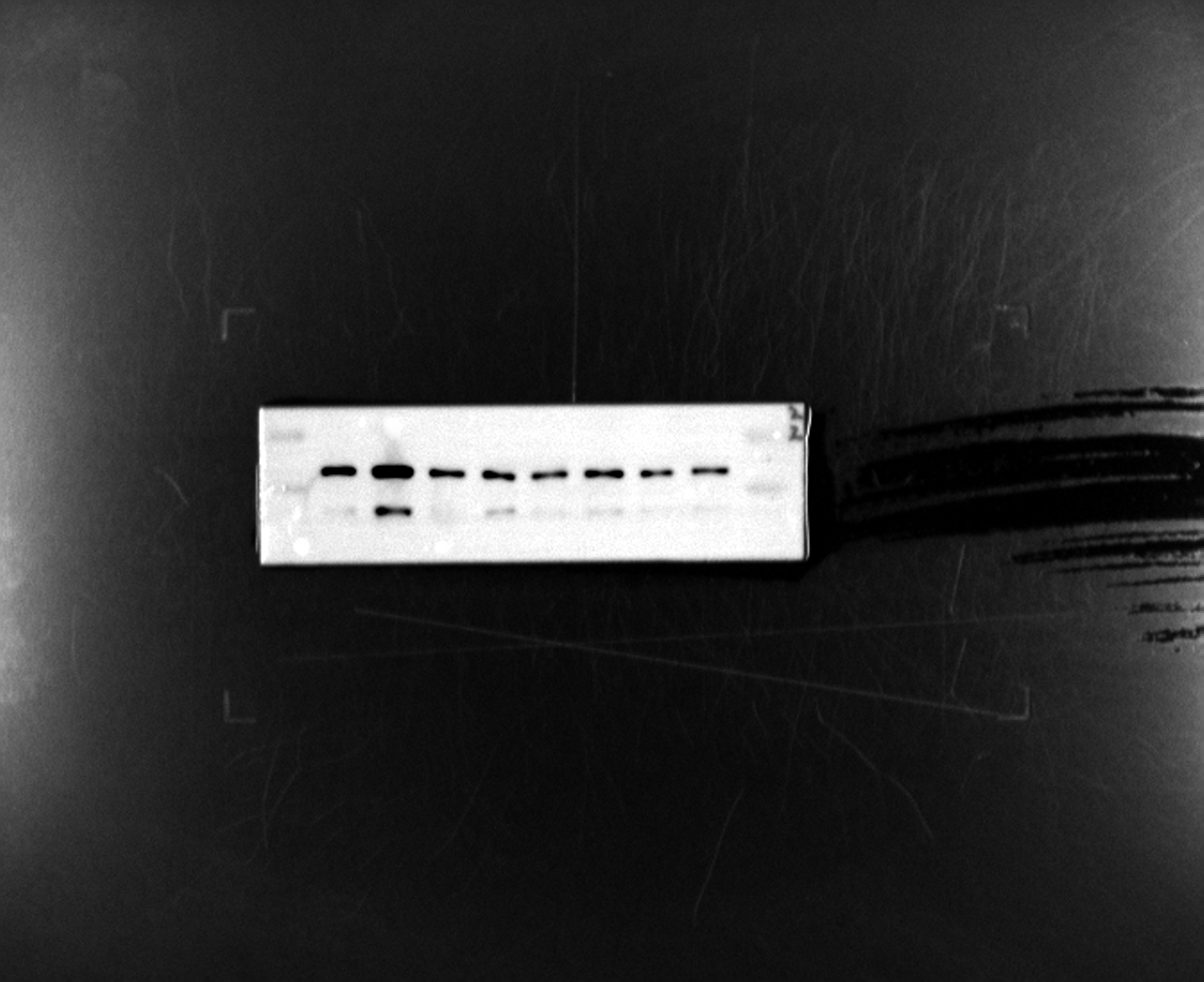

Supplement: Supplementary file 7 [file DataSheet_3.zip › fig5H-sma-2-0.Tif]

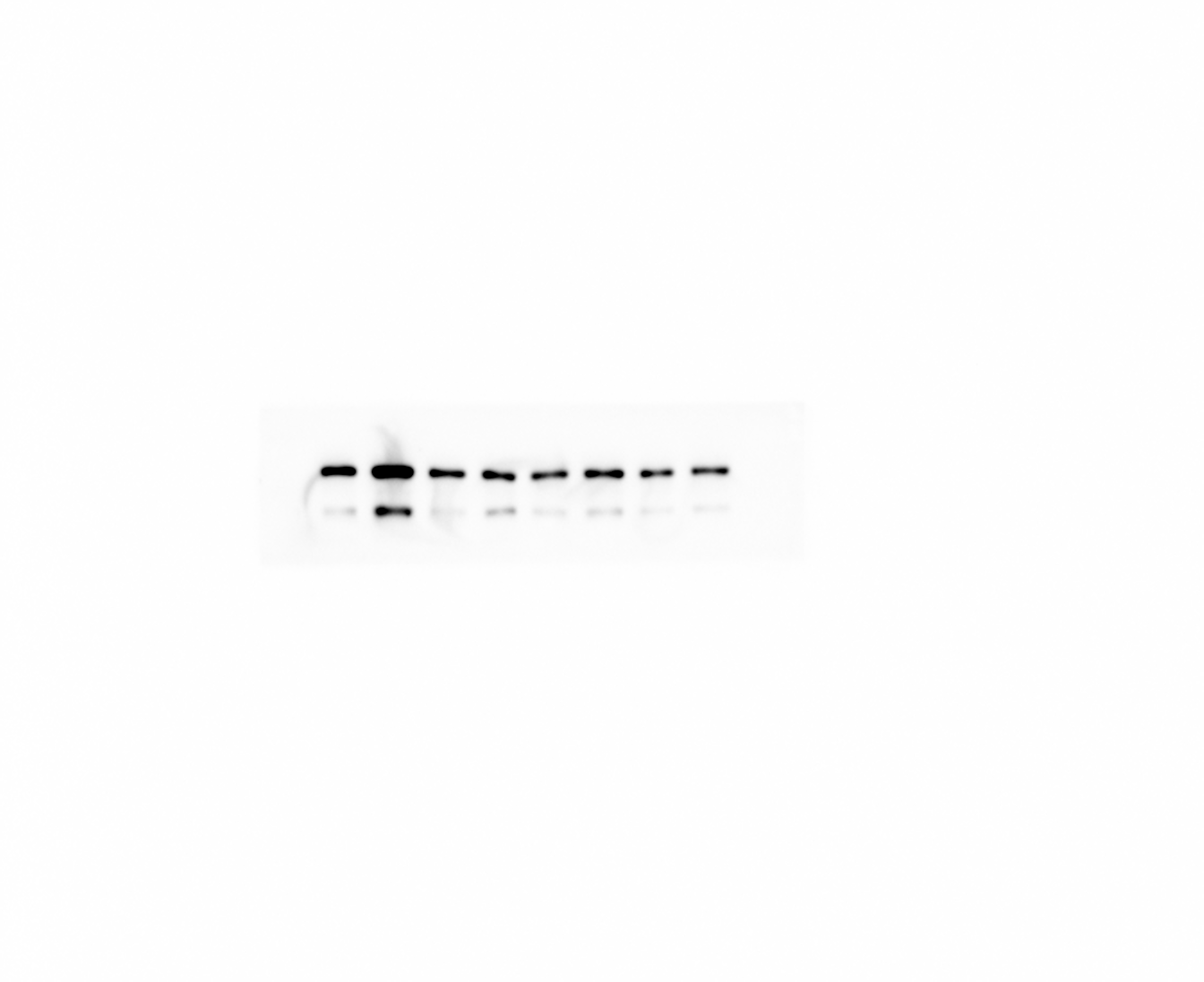

Supplement: Supplementary file 7 [file DataSheet_3.zip › fig5H-sma-2.Tif]

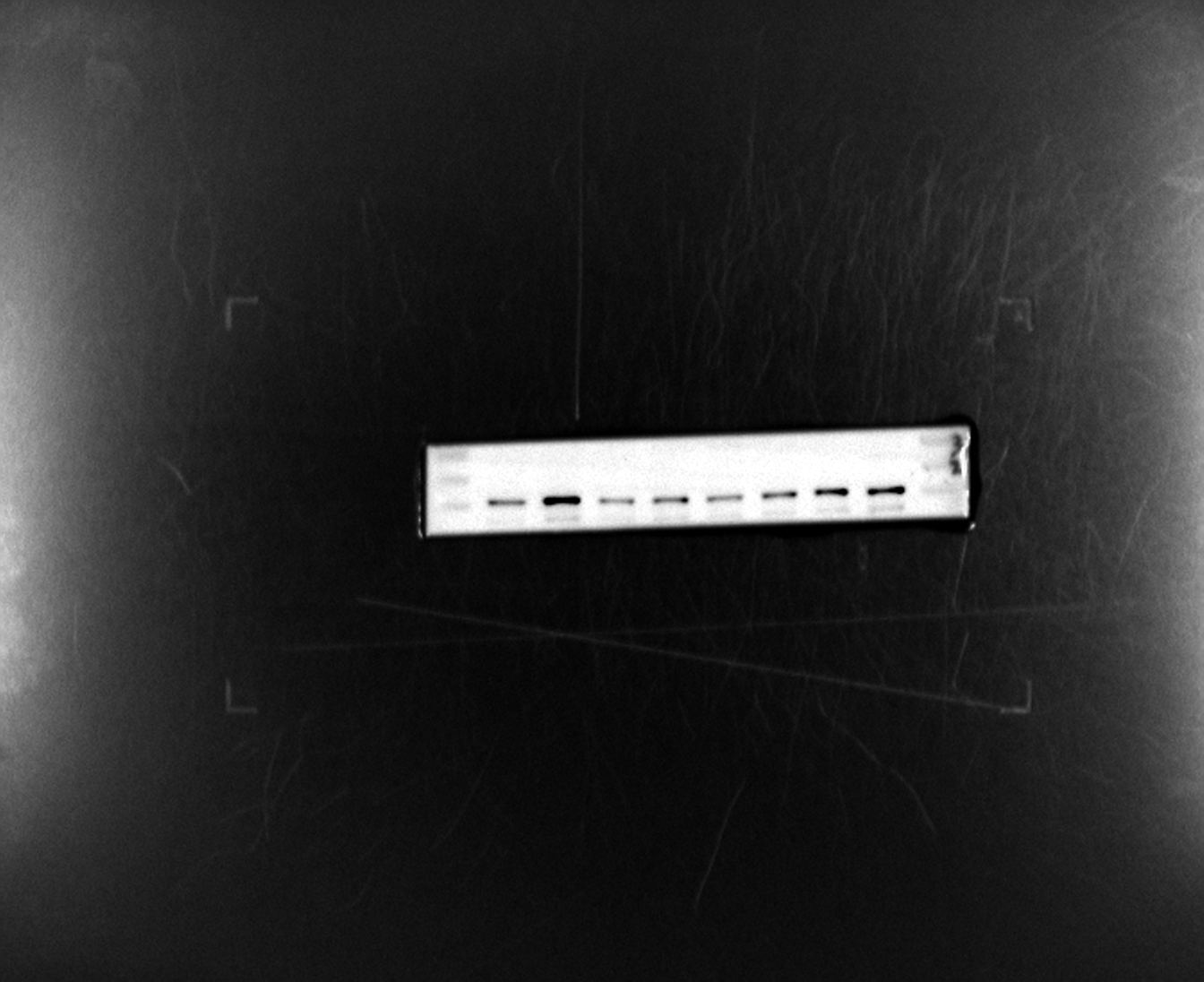

Supplement: Supplementary file 7 [file DataSheet_3.zip › fig5H-sma-3-0.Tif]

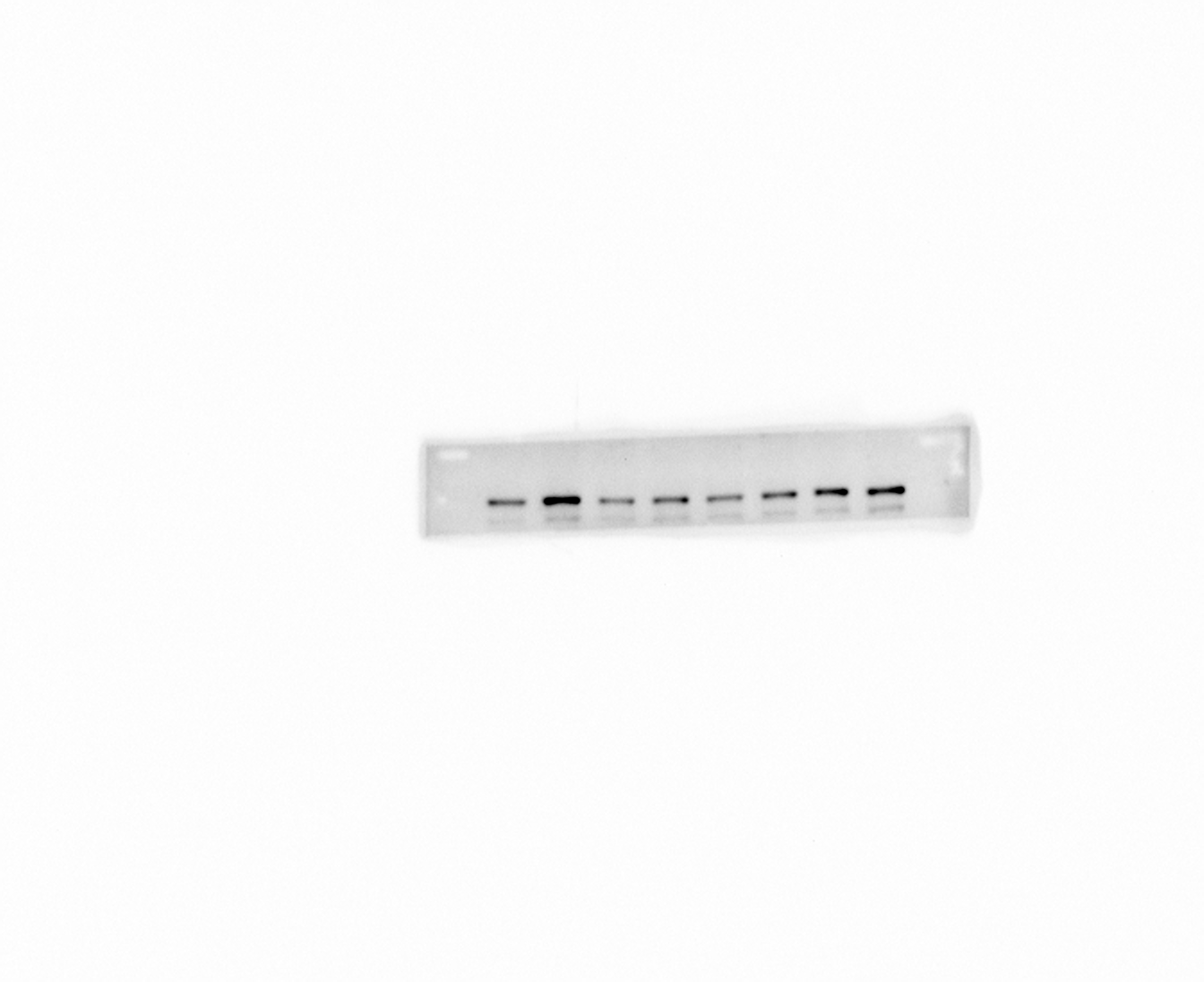

Supplement: Supplementary file 7 [file DataSheet_3.zip › fig5H-sma-3.Tif]

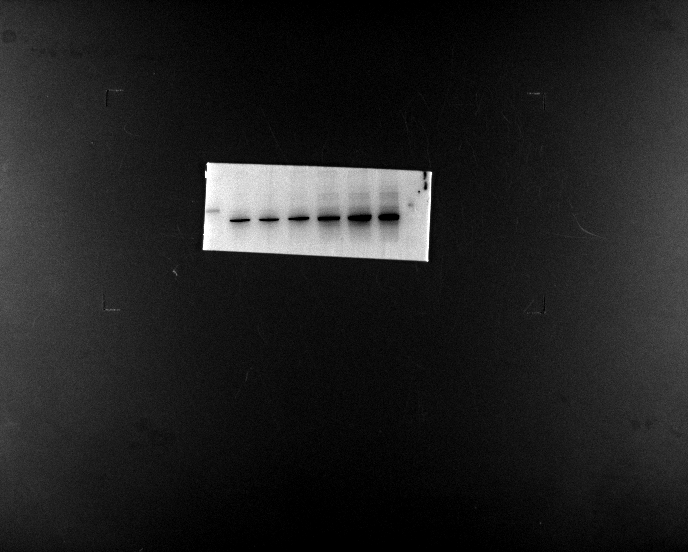

Supplement: Supplementary file 7 [file DataSheet_3.zip › figS1B-col1-0.Tif]

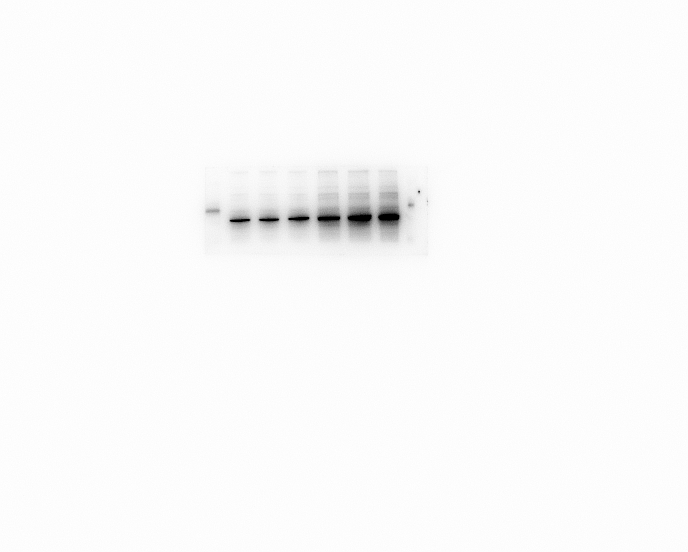

Supplement: Supplementary file 7 [file DataSheet_3.zip › figS1B-col1.Tif]

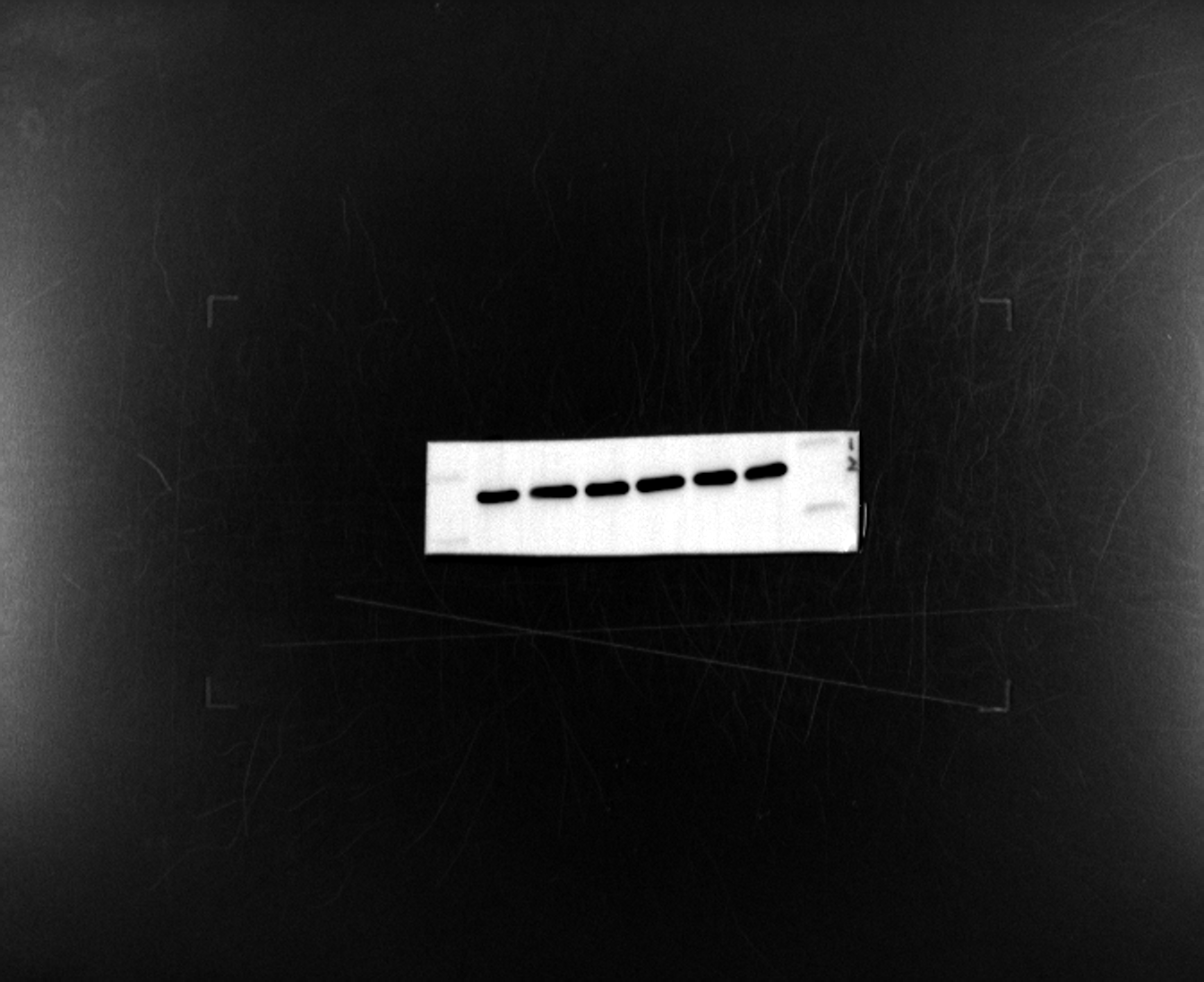

Supplement: Supplementary file 7 [file DataSheet_3.zip › figS1B-gap-0.Tif]

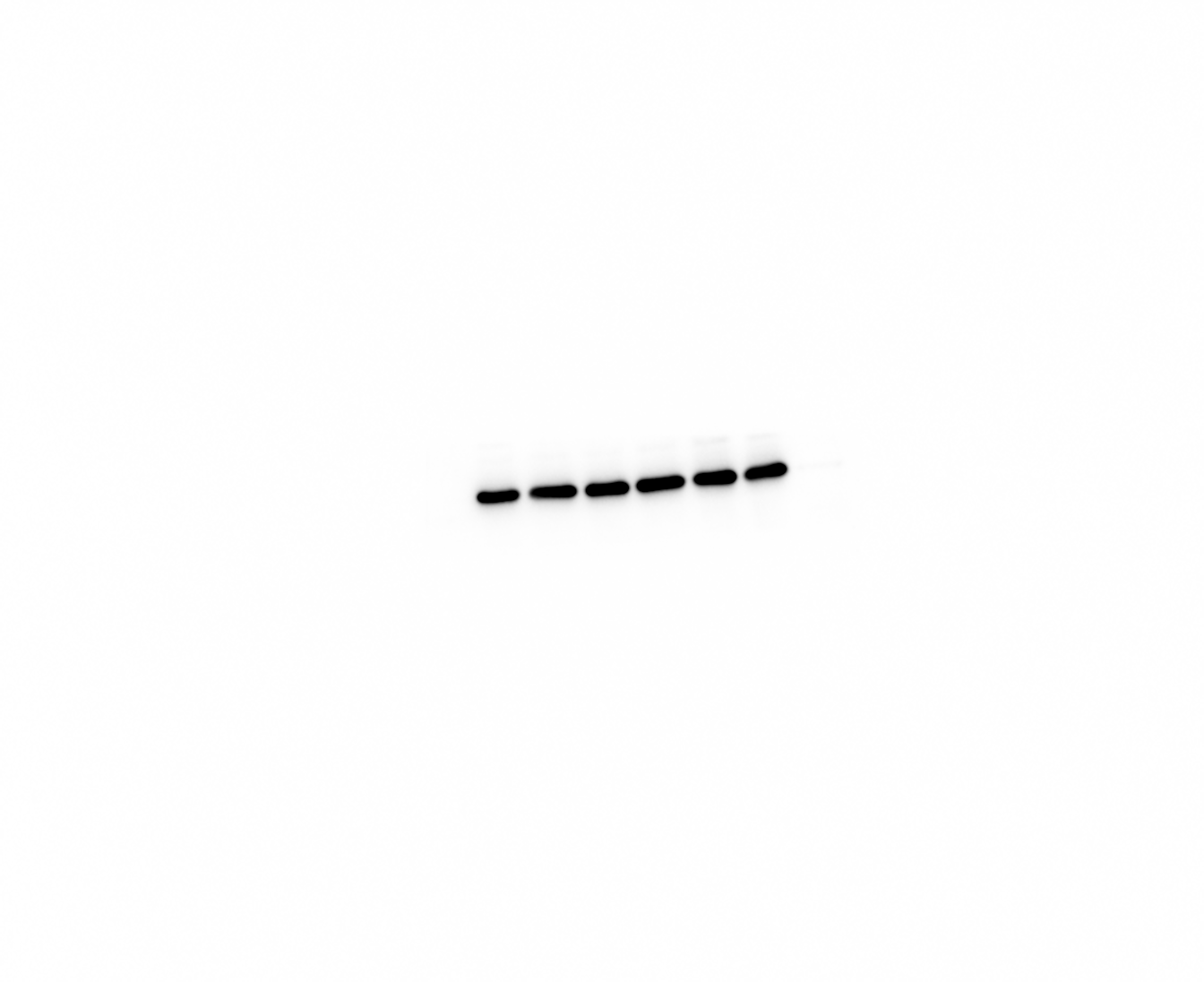

Supplement: Supplementary file 7 [file DataSheet_3.zip › figS1B-gap.Tif]

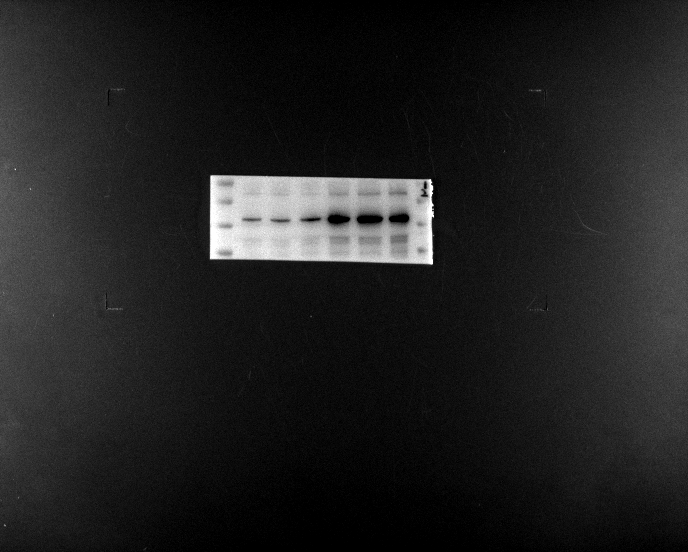

Supplement: Supplementary file 7 [file DataSheet_3.zip › figS1B-sma-0.Tif]

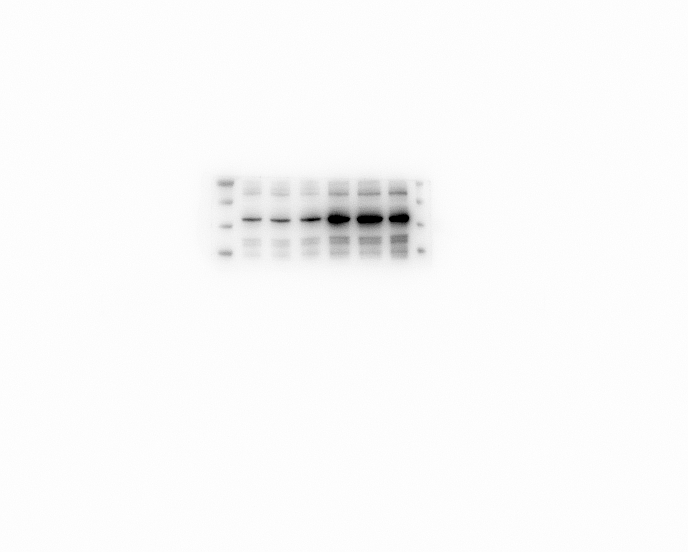

Supplement: Supplementary file 7 [file DataSheet_3.zip › figS1B-sma.Tif]

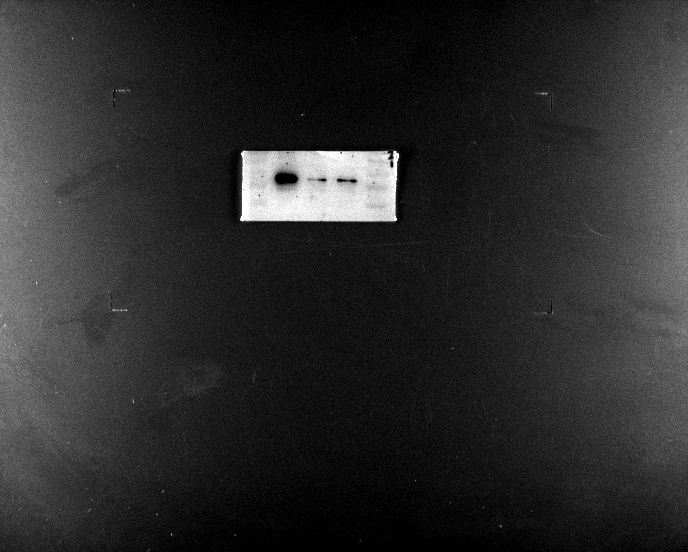

Supplement: Supplementary file 7 [file DataSheet_3.zip › figS2B-cald1-0.Tif]

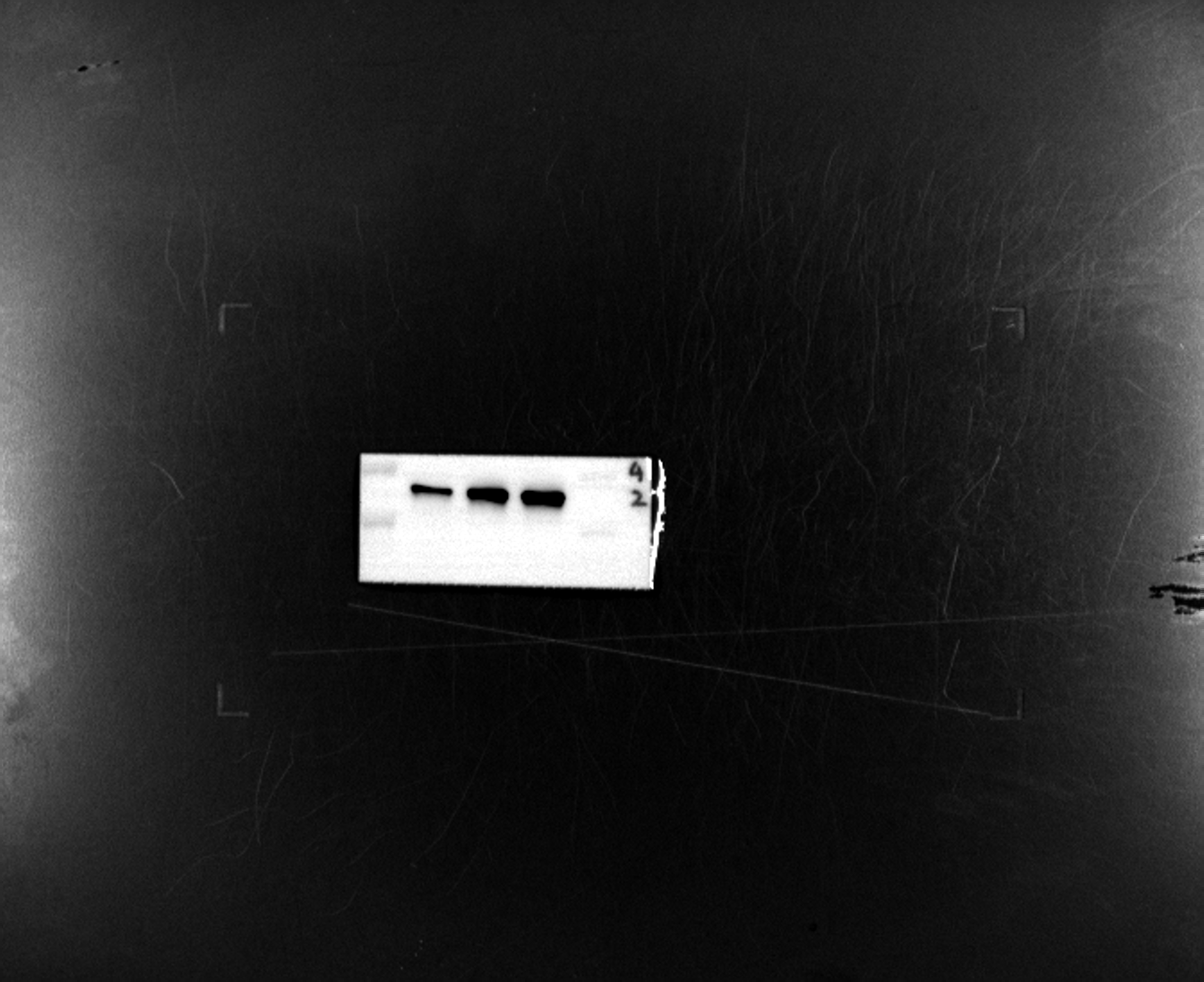

Supplement: Supplementary file 7 [file DataSheet_3.zip › figS2B-CALD1-gapdh-0.Tif]

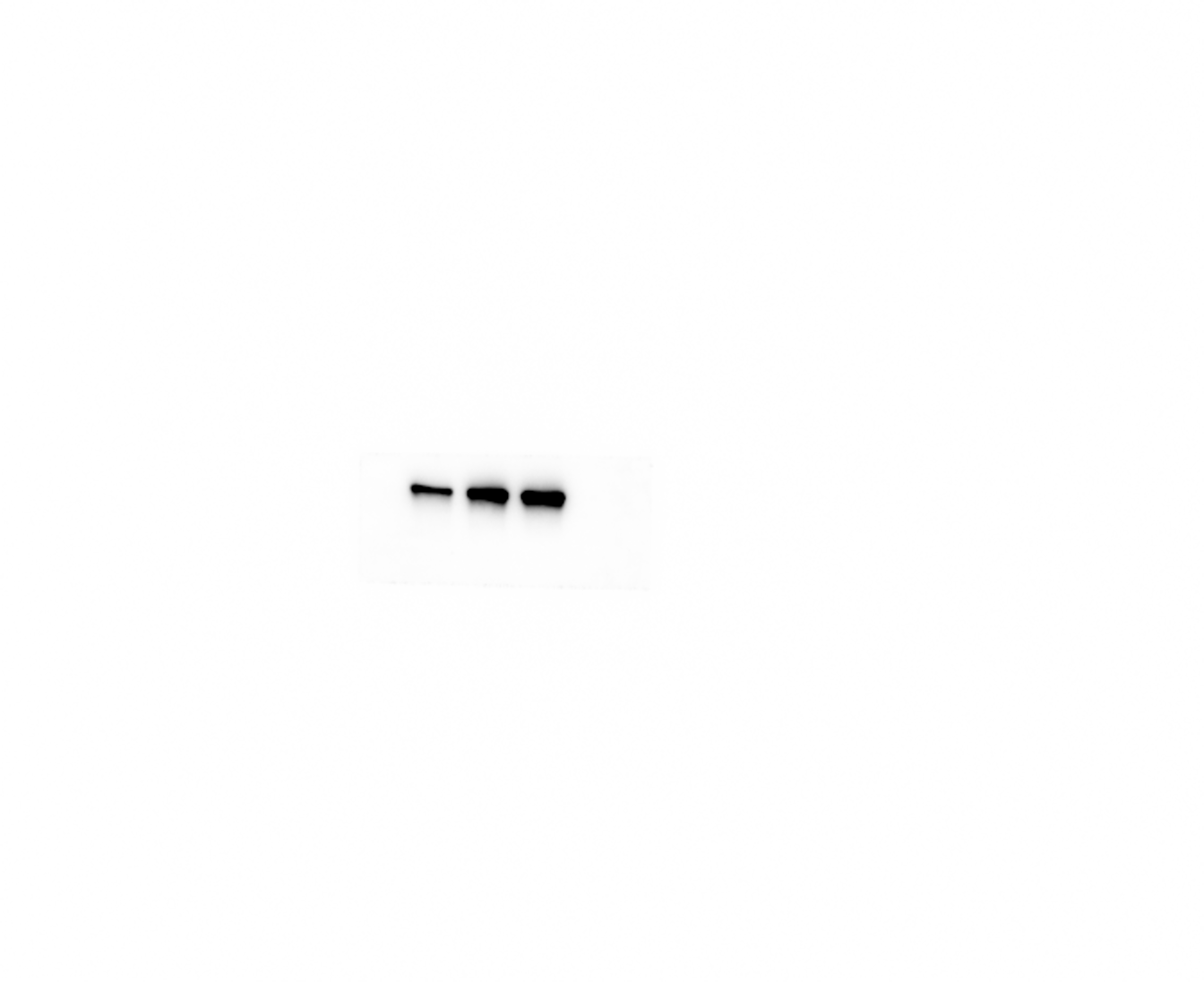

Supplement: Supplementary file 7 [file DataSheet_3.zip › figS2B-CALD1-gapdh.Tif]

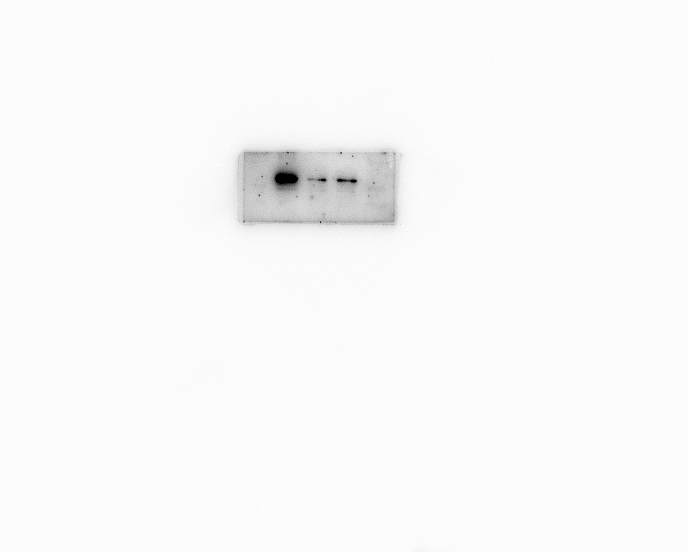

Supplement: Supplementary file 7 [file DataSheet_3.zip › figS2B-cald1.Tif]

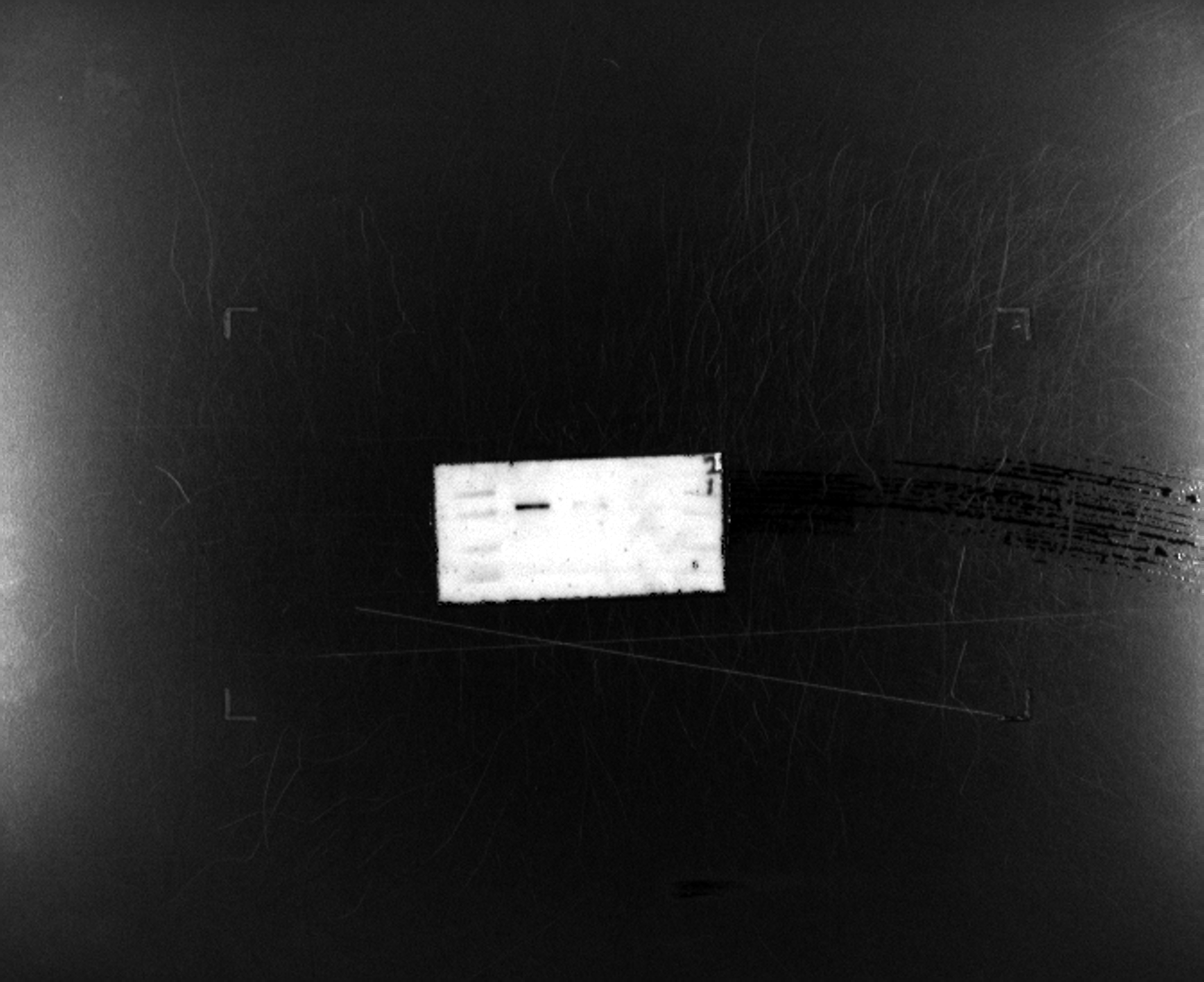

Supplement: Supplementary file 7 [file DataSheet_3.zip › figS2B-cdh2-0.Tif]

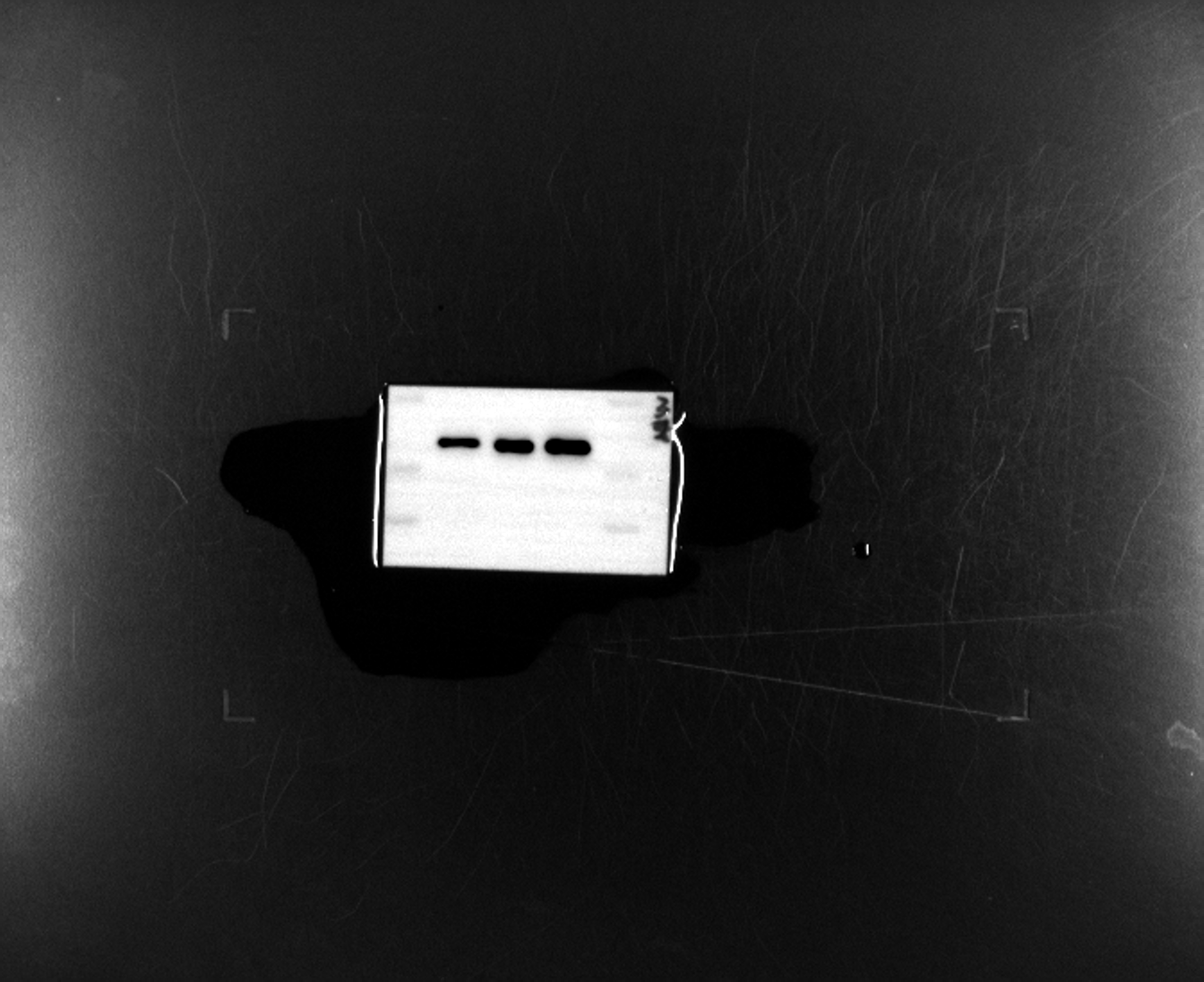

Supplement: Supplementary file 7 [file DataSheet_3.zip › figS2B-CDH2-gapdh-0.Tif]

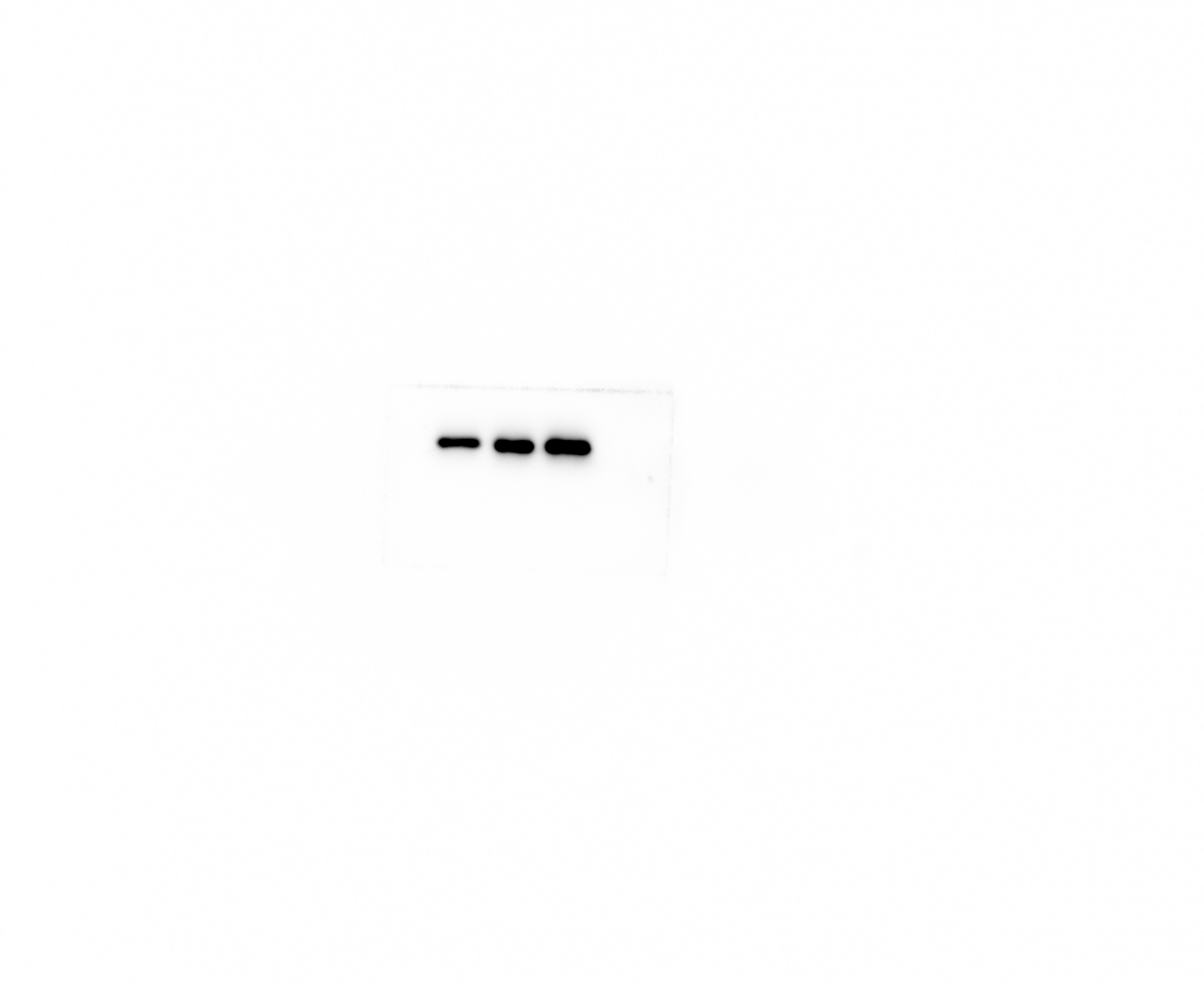

Supplement: Supplementary file 7 [file DataSheet_3.zip › figS2B-CDH2-gapdh.Tif]

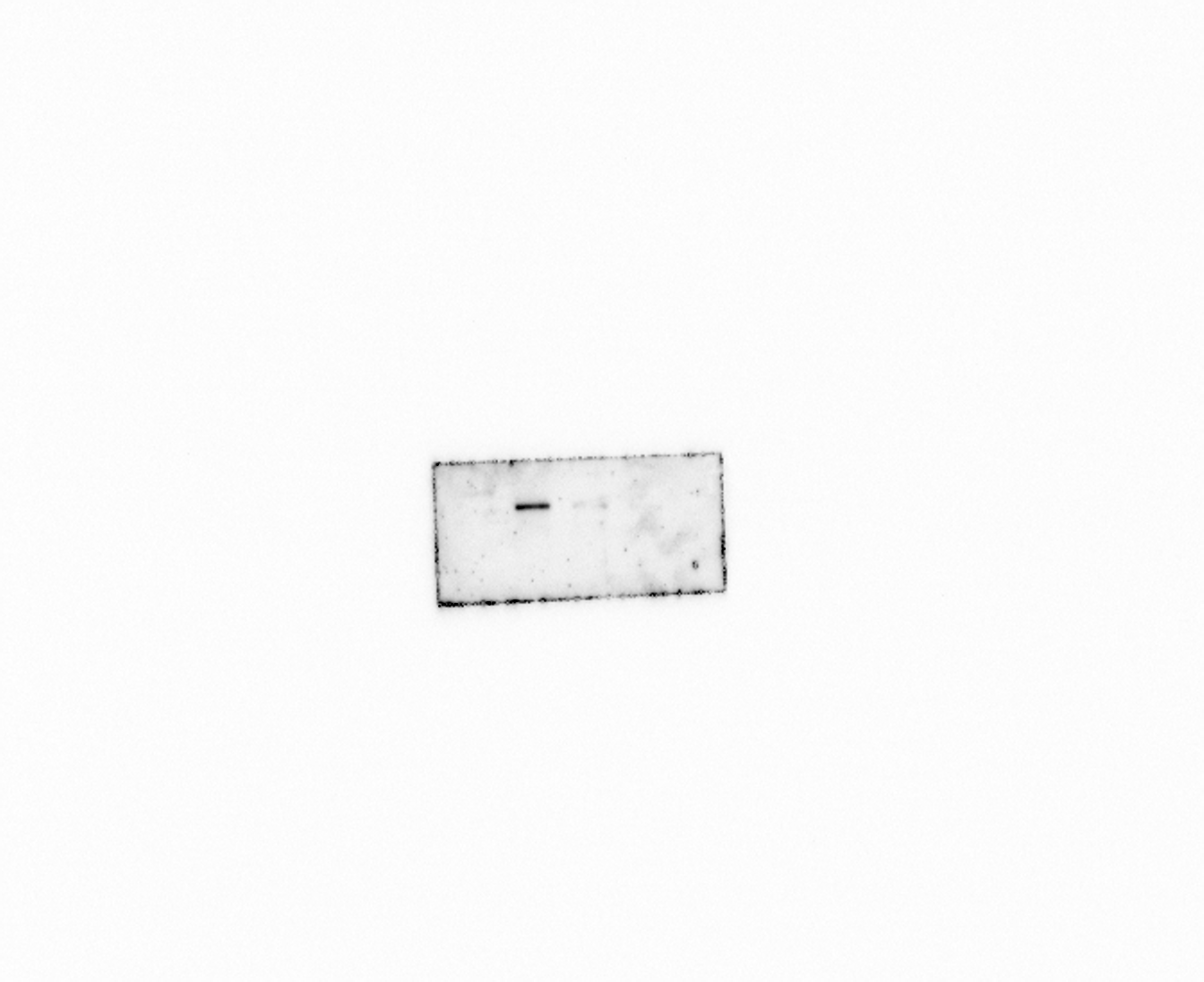

Supplement: Supplementary file 7 [file DataSheet_3.zip › figS2B-cdh2.Tif]

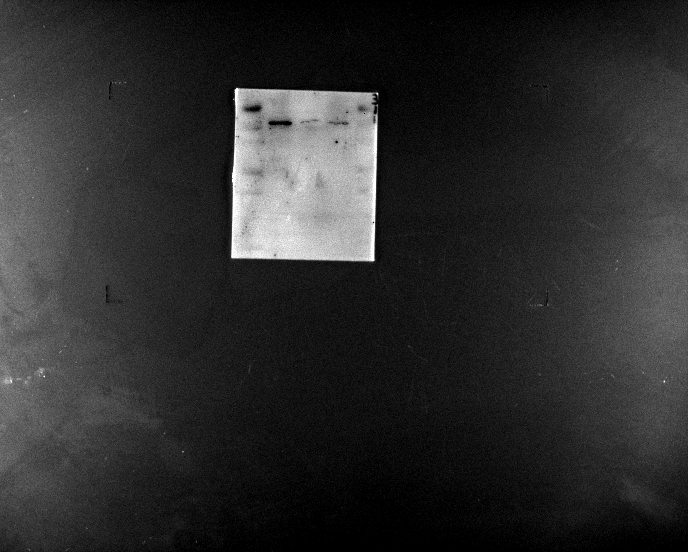

Supplement: Supplementary file 7 [file DataSheet_3.zip › figS2B-postn-0.Tif]

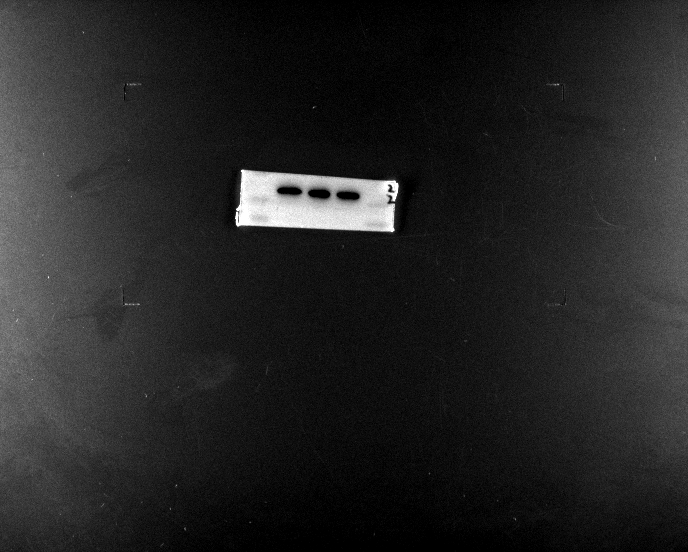

Supplement: Supplementary file 7 [file DataSheet_3.zip › figS2B-POSTN-gapdh-0.Tif]

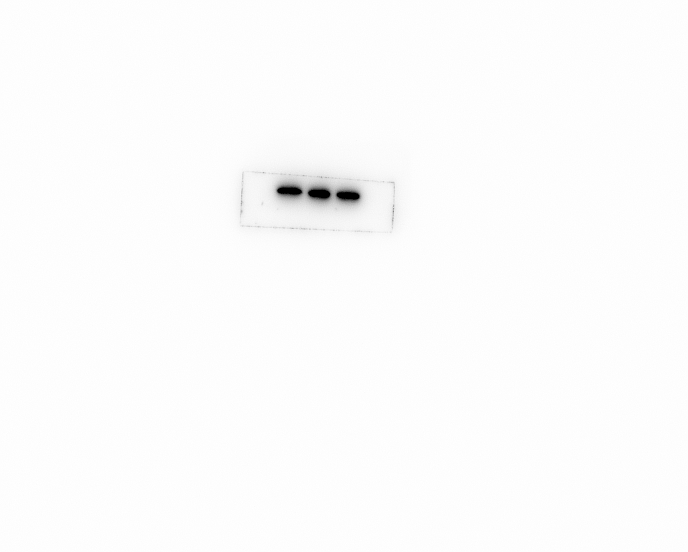

Supplement: Supplementary file 7 [file DataSheet_3.zip › figS2B-POSTN-gapdh.Tif]

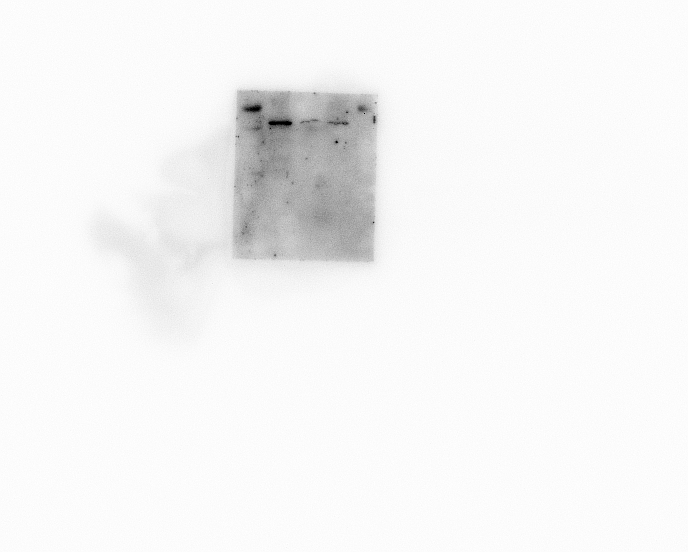

Supplement: Supplementary file 7 [file DataSheet_3.zip › figS2B-postn.Tif]

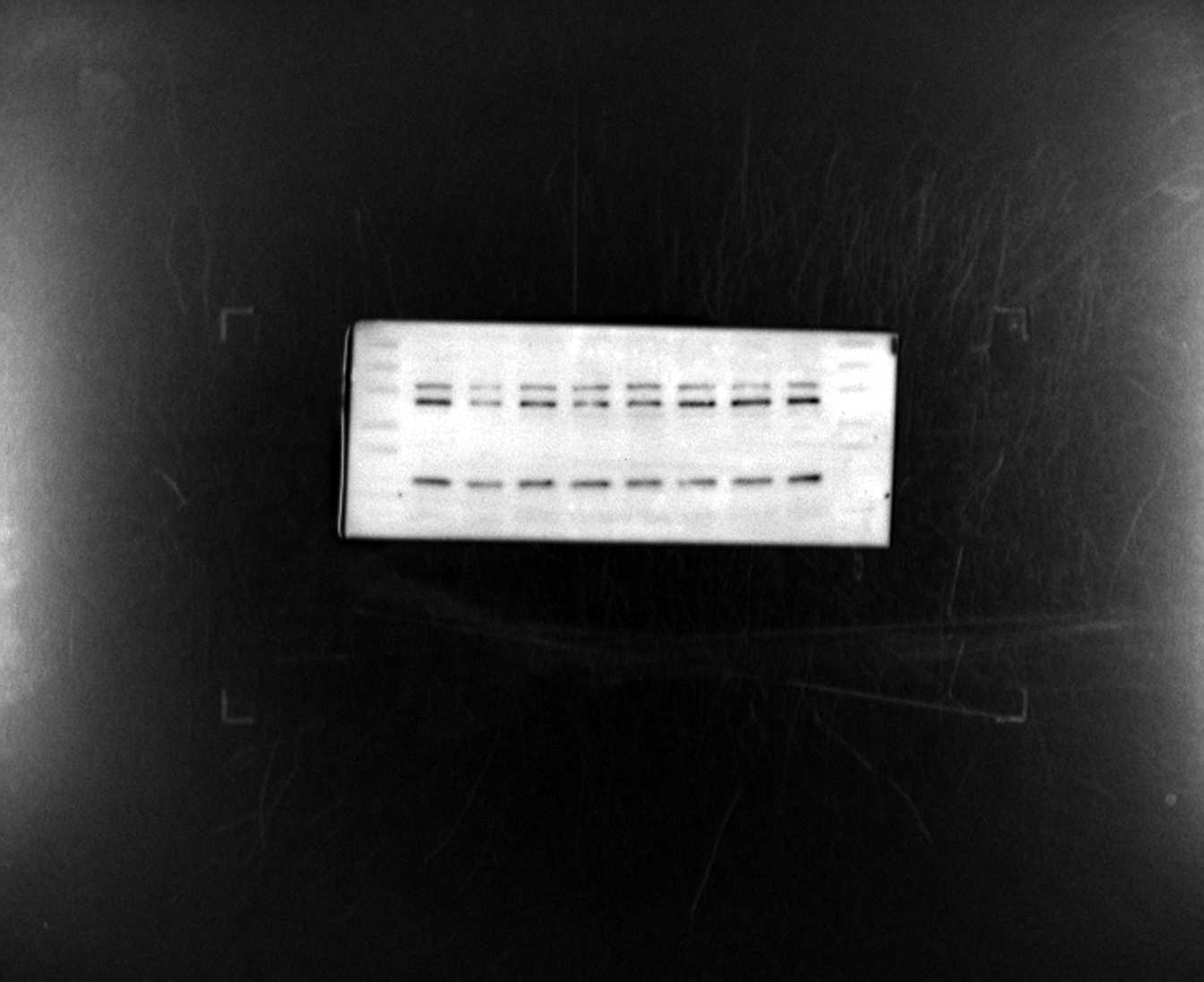

Supplement: Supplementary file 7 [file DataSheet_3.zip › figS2D-bax-1-0.Tif]

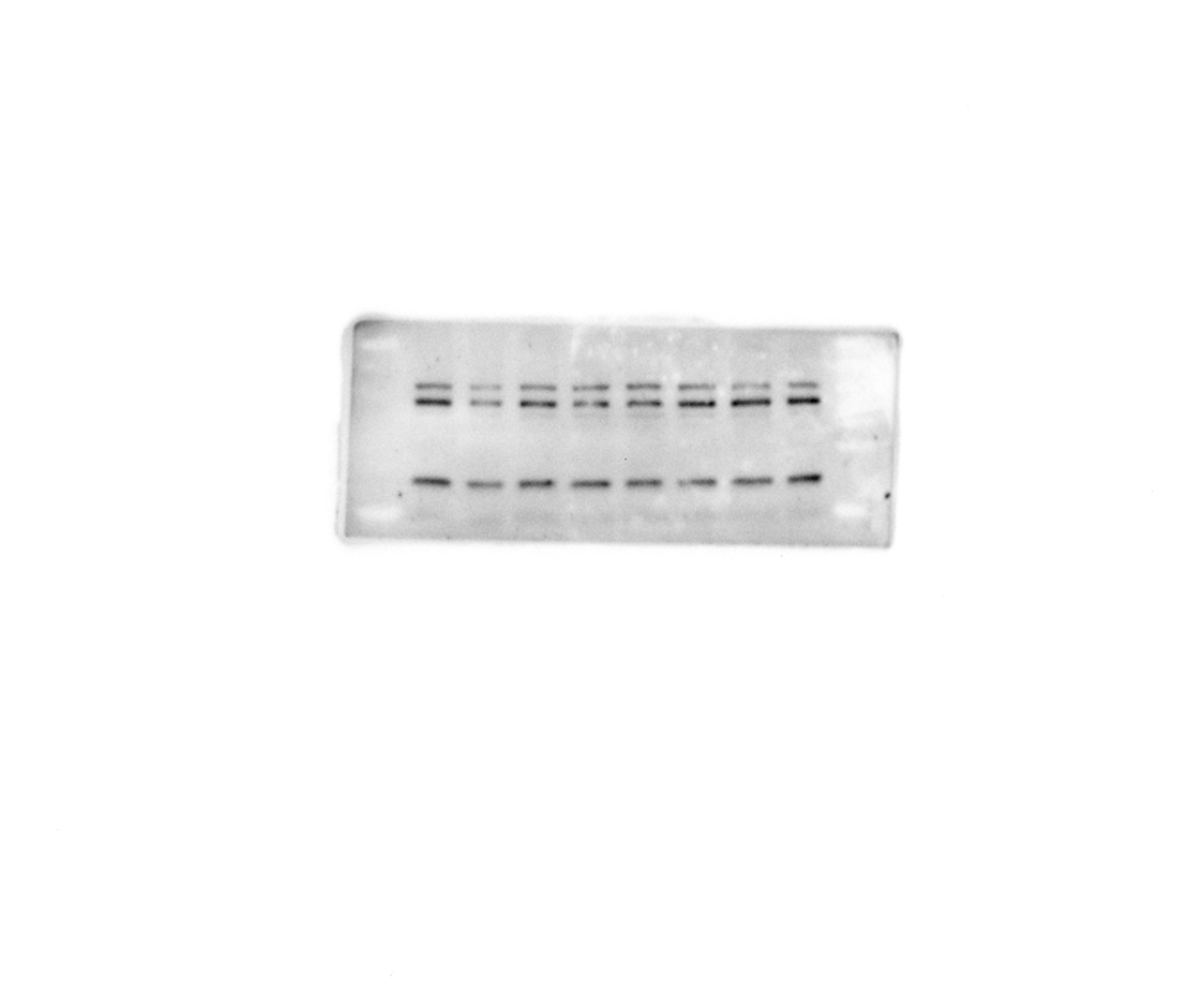

Supplement: Supplementary file 7 [file DataSheet_3.zip › figS2D-bax-1.Tif]

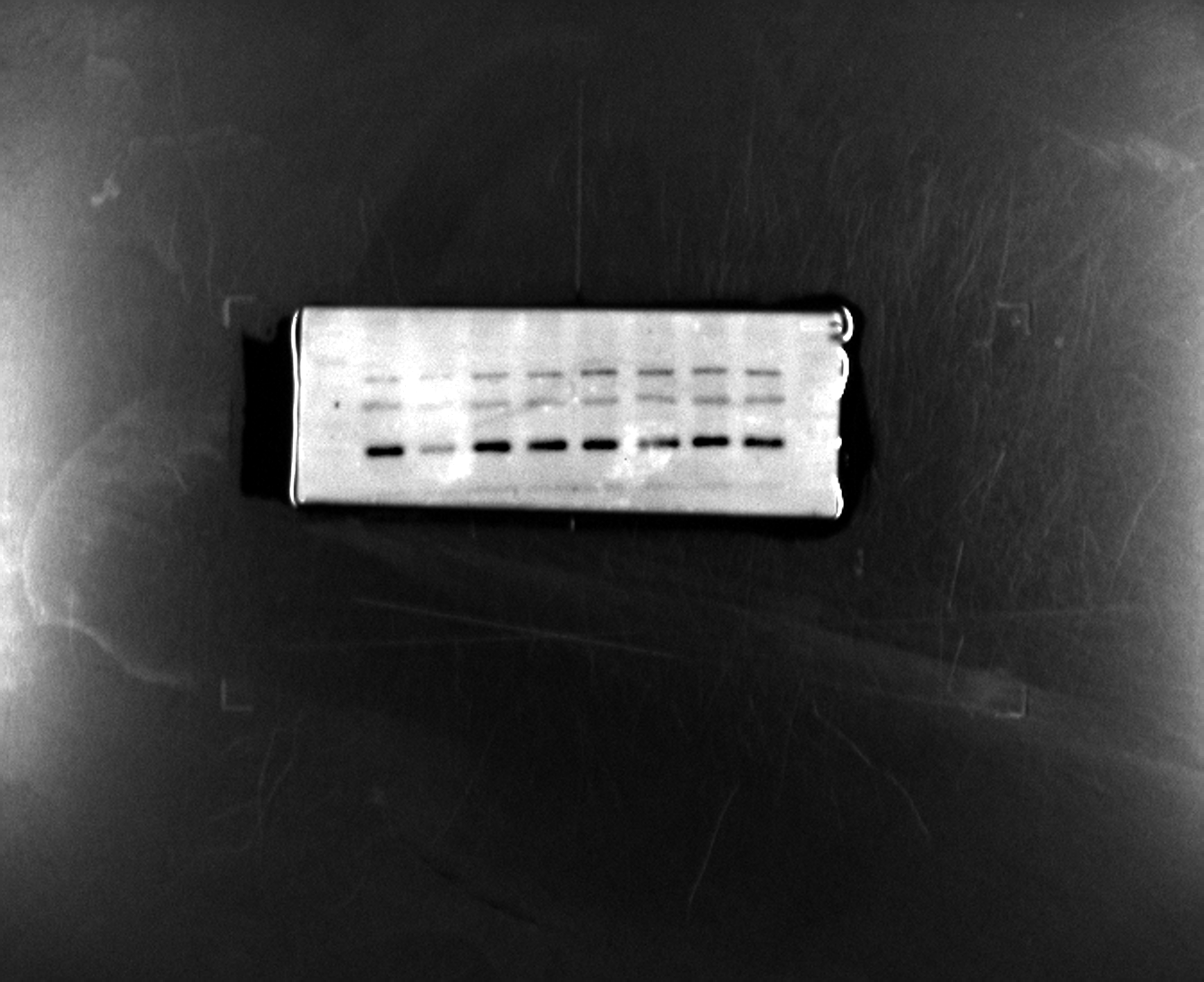

Supplement: Supplementary file 7 [file DataSheet_3.zip › figS2D-bax-2-0.Tif]

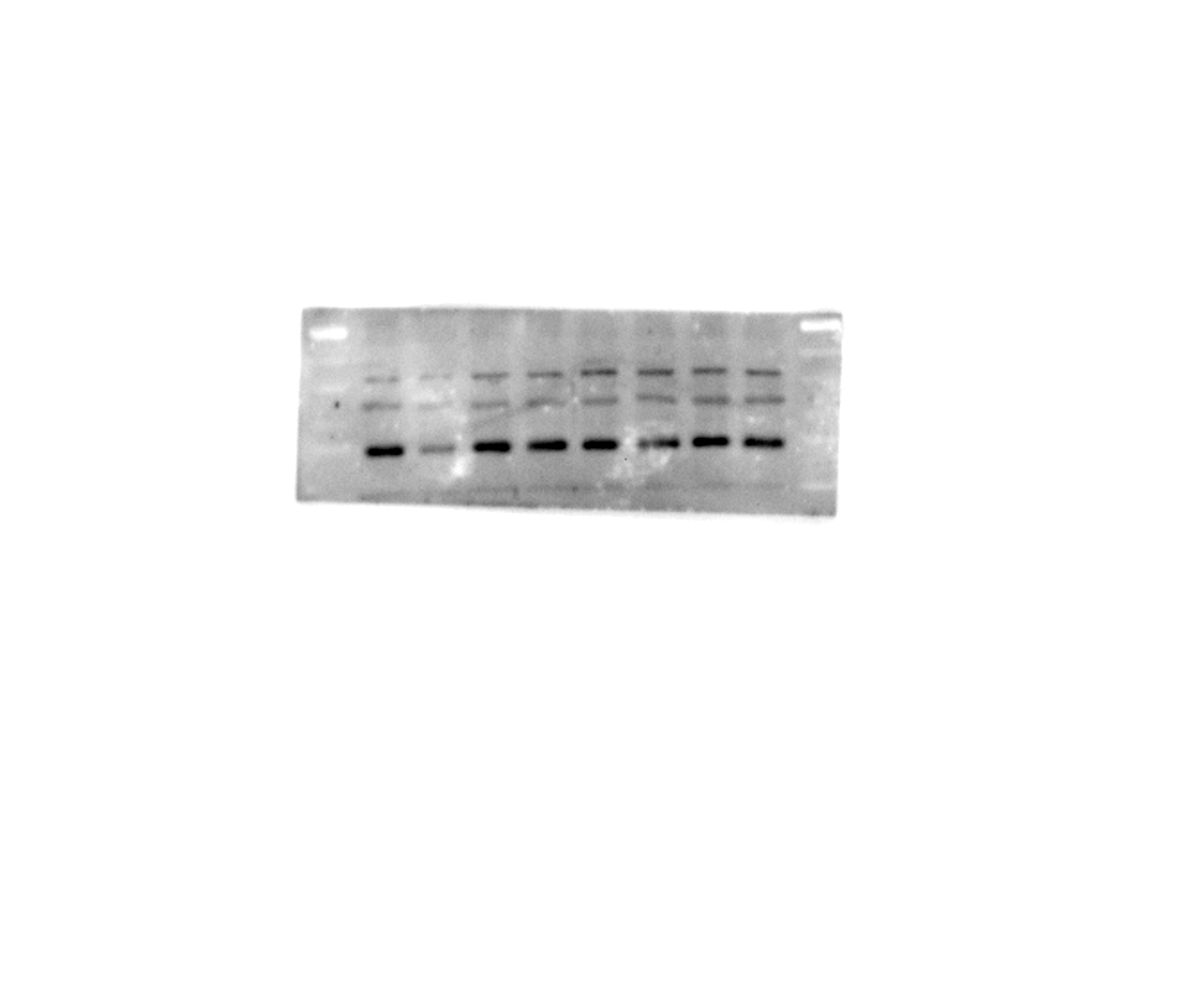

Supplement: Supplementary file 7 [file DataSheet_3.zip › figS2D-bax-2.Tif]

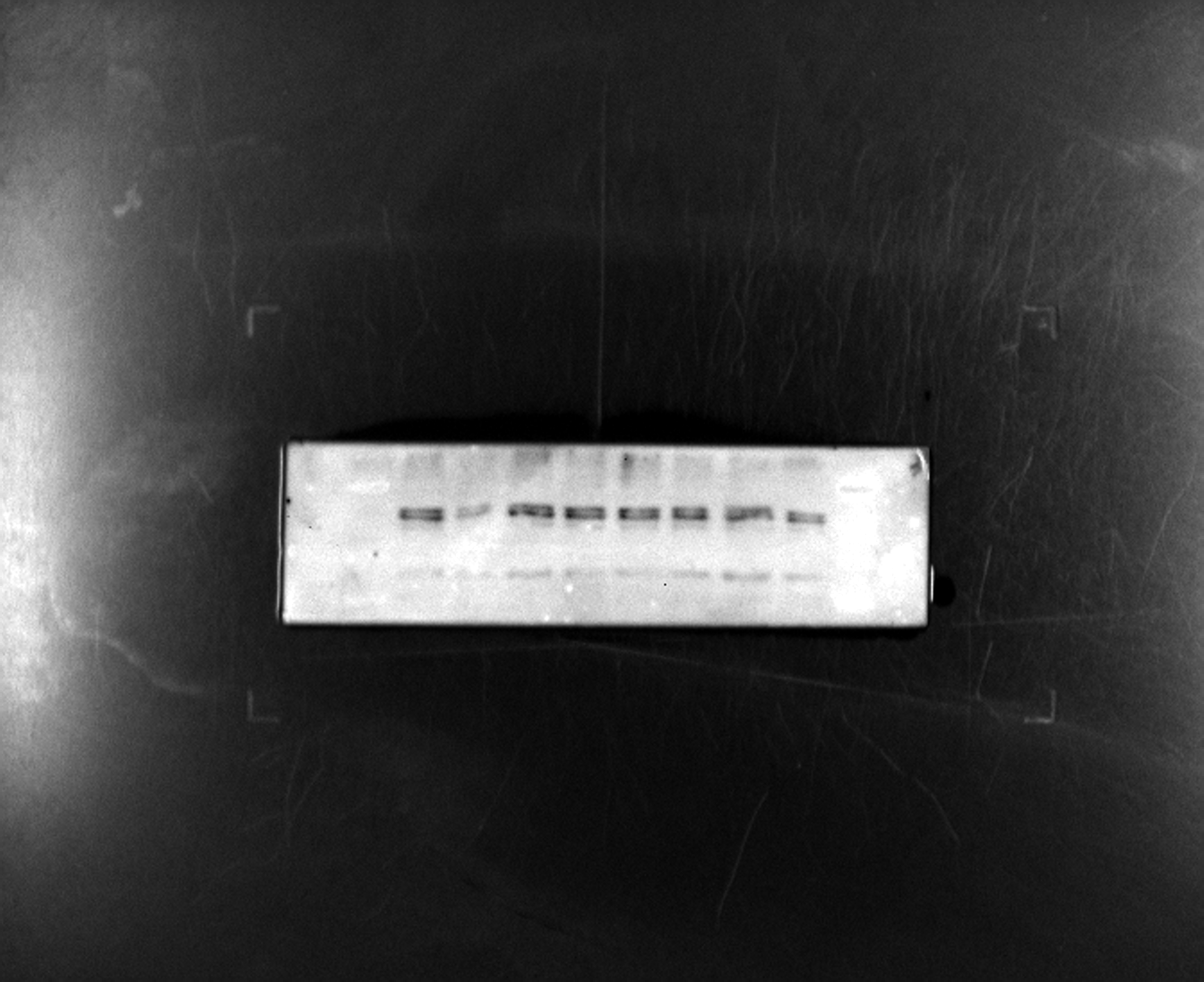

Supplement: Supplementary file 7 [file DataSheet_3.zip › figS2D-bax-3-0.Tif]

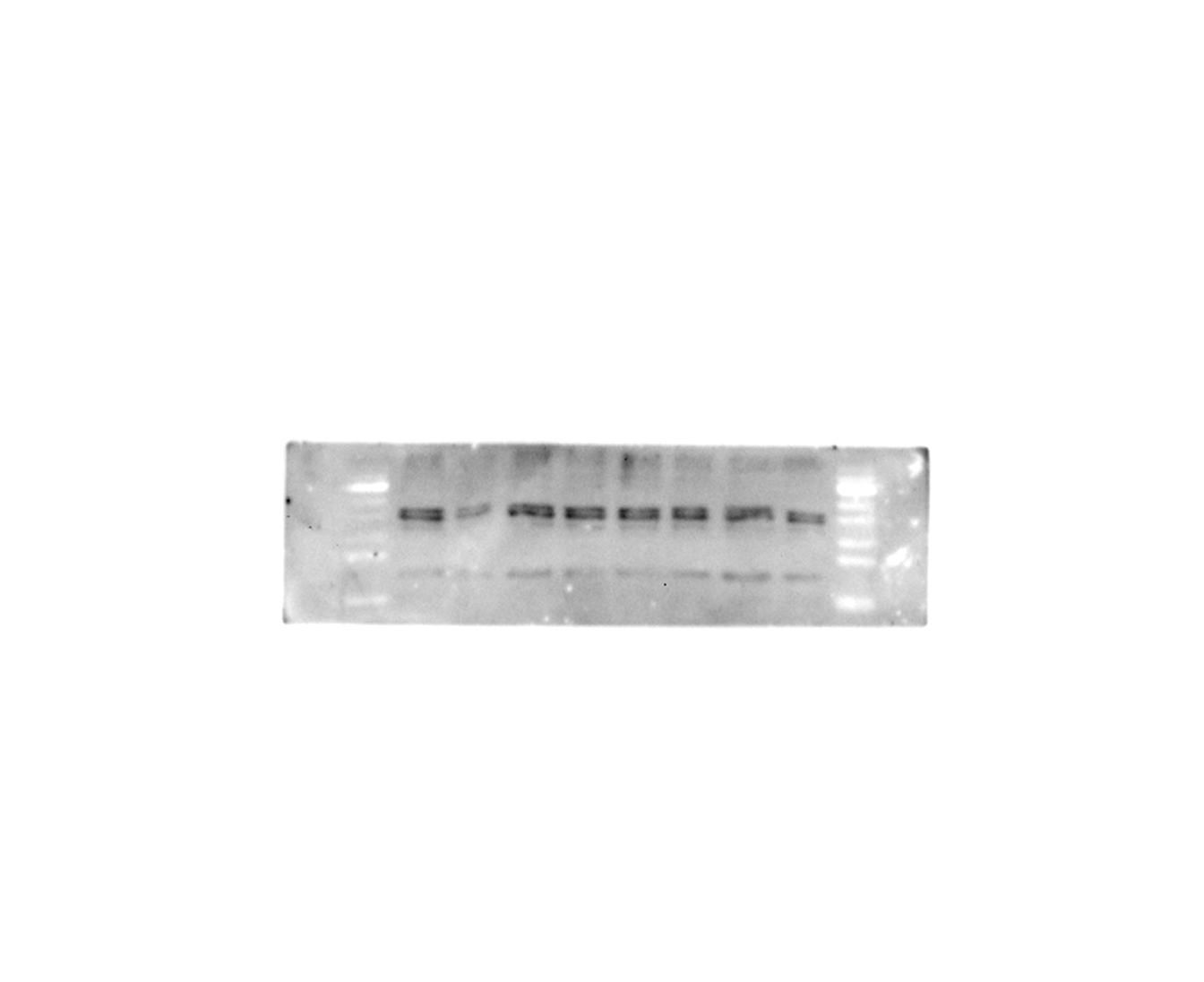

Supplement: Supplementary file 7 [file DataSheet_3.zip › figS2D-bax-3.Tif]

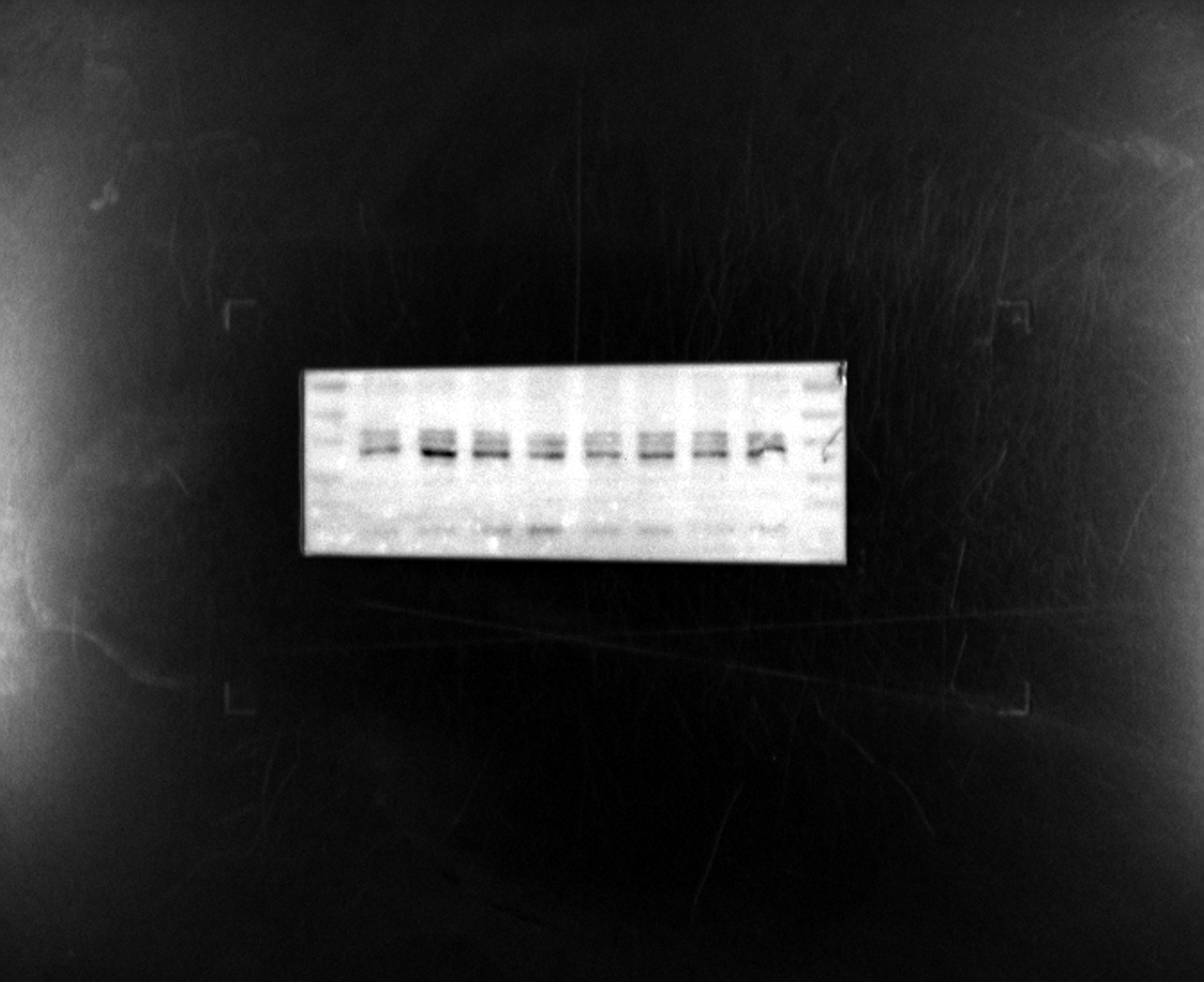

Supplement: Supplementary file 7 [file DataSheet_3.zip › figS2D-bcl2-1-0.Tif]

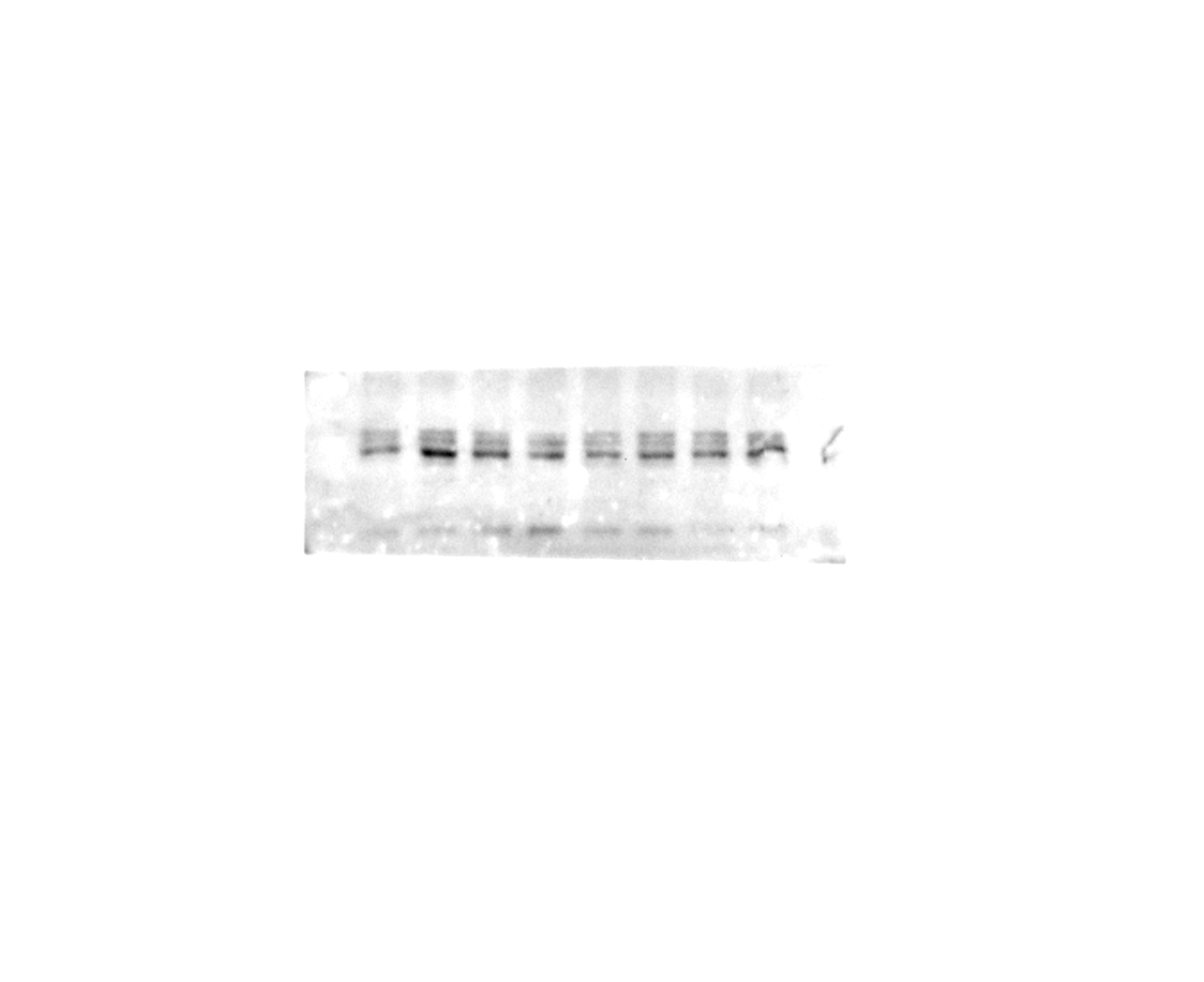

Supplement: Supplementary file 7 [file DataSheet_3.zip › figS2D-bcl2-1.Tif]

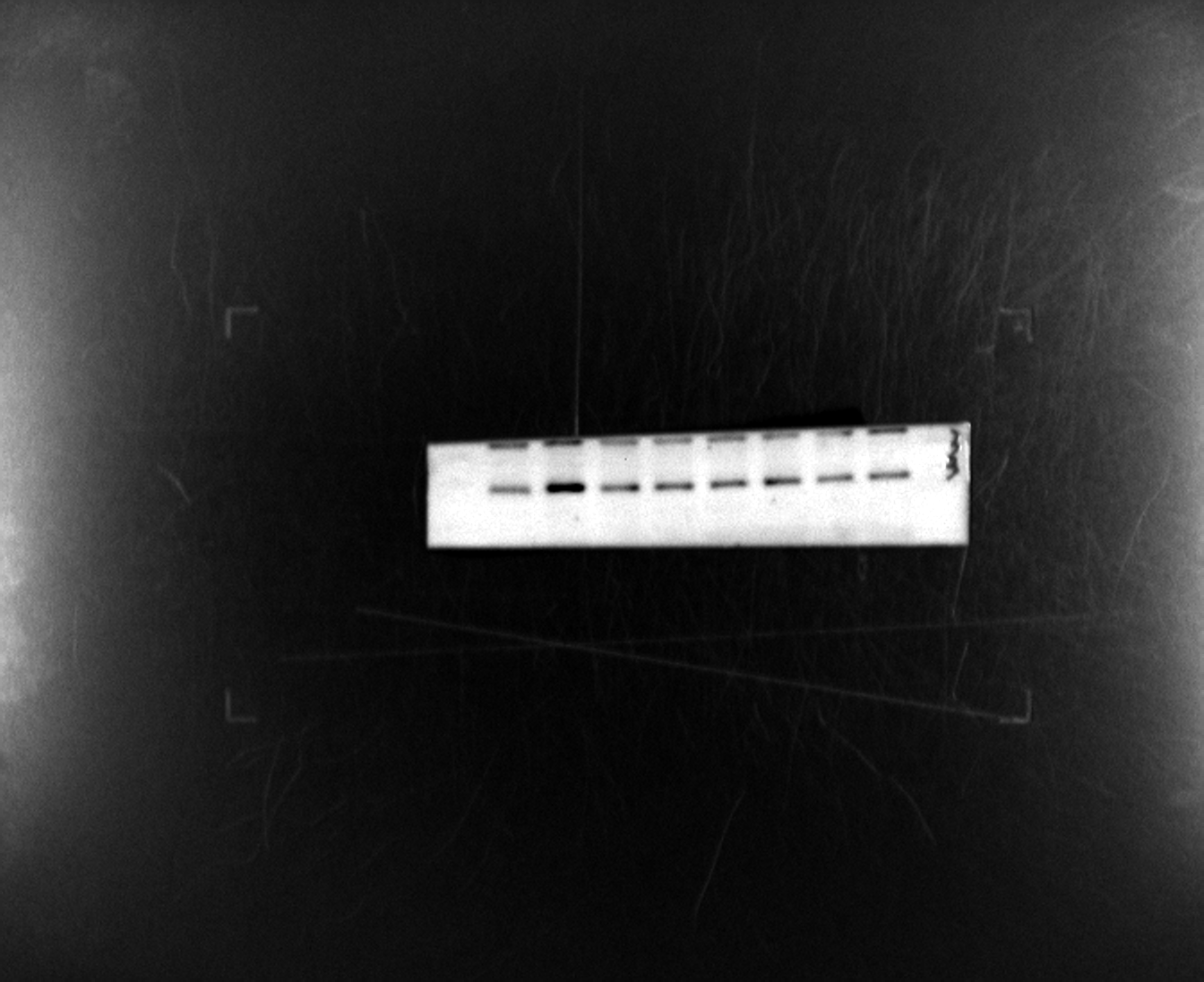

Supplement: Supplementary file 7 [file DataSheet_3.zip › figS2D-bcl2-2-0.Tif]

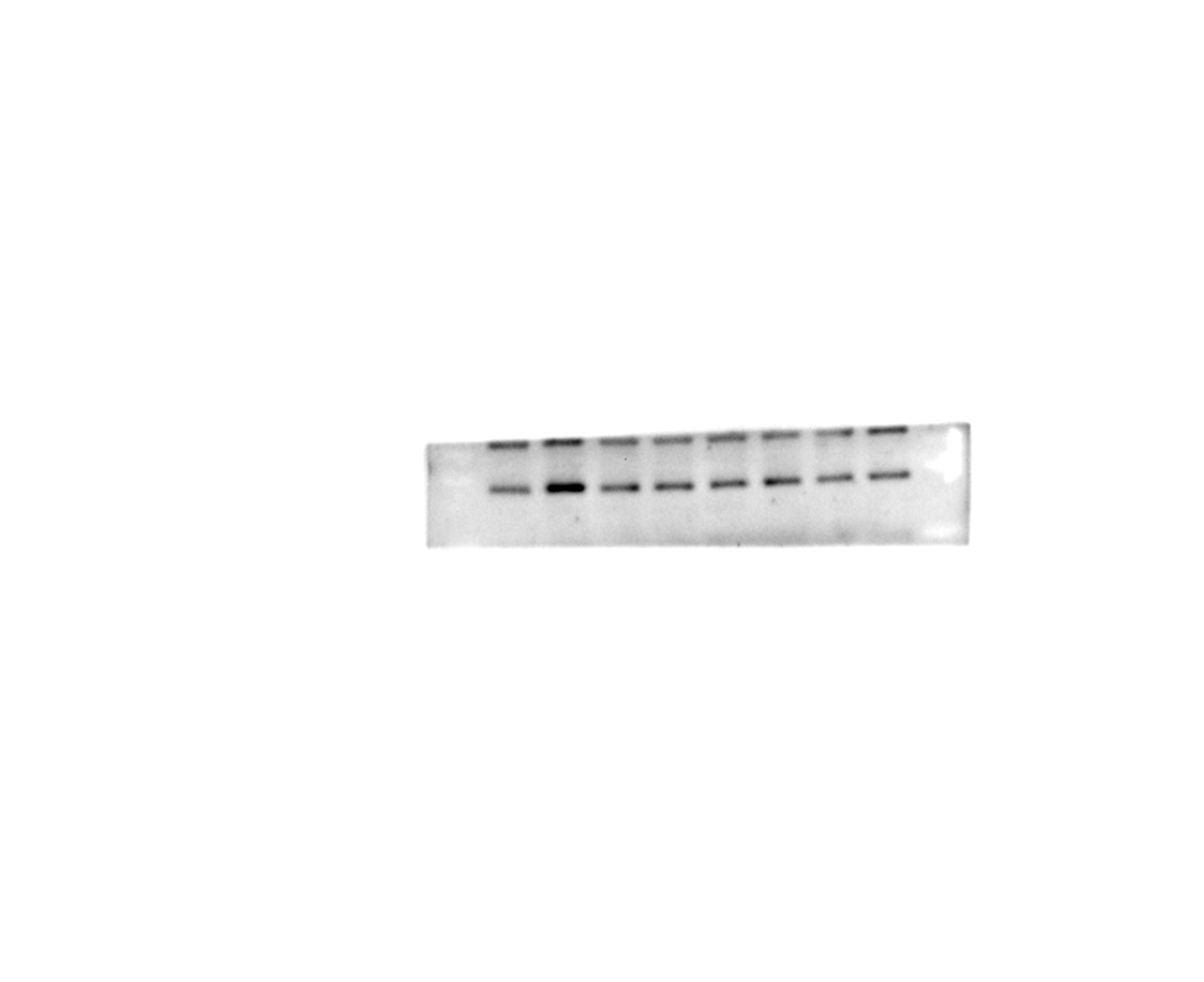

Supplement: Supplementary file 7 [file DataSheet_3.zip › figS2D-bcl2-2.Tif]

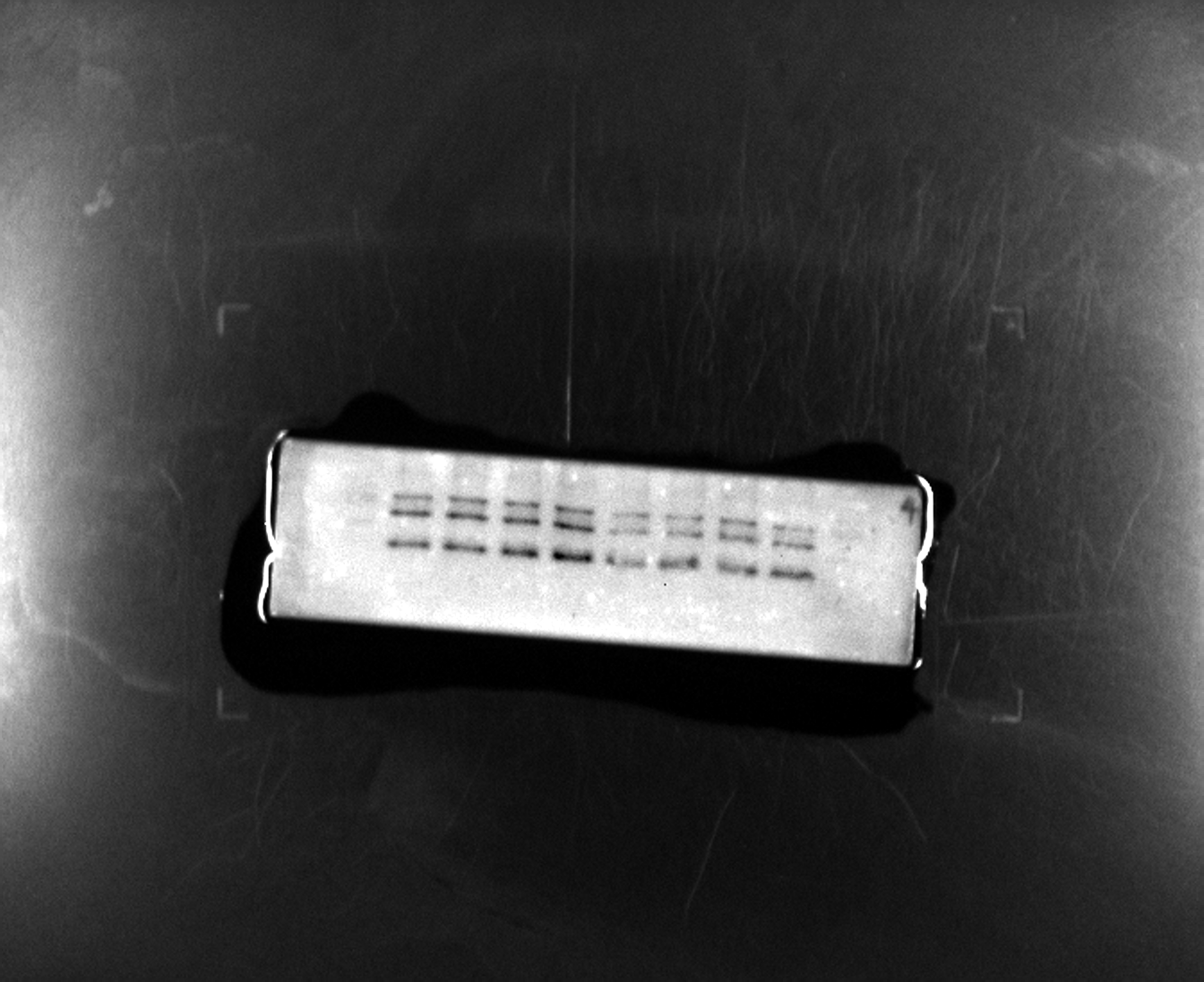

Supplement: Supplementary file 7 [file DataSheet_3.zip › figS2D-bcl2-3-0.Tif]

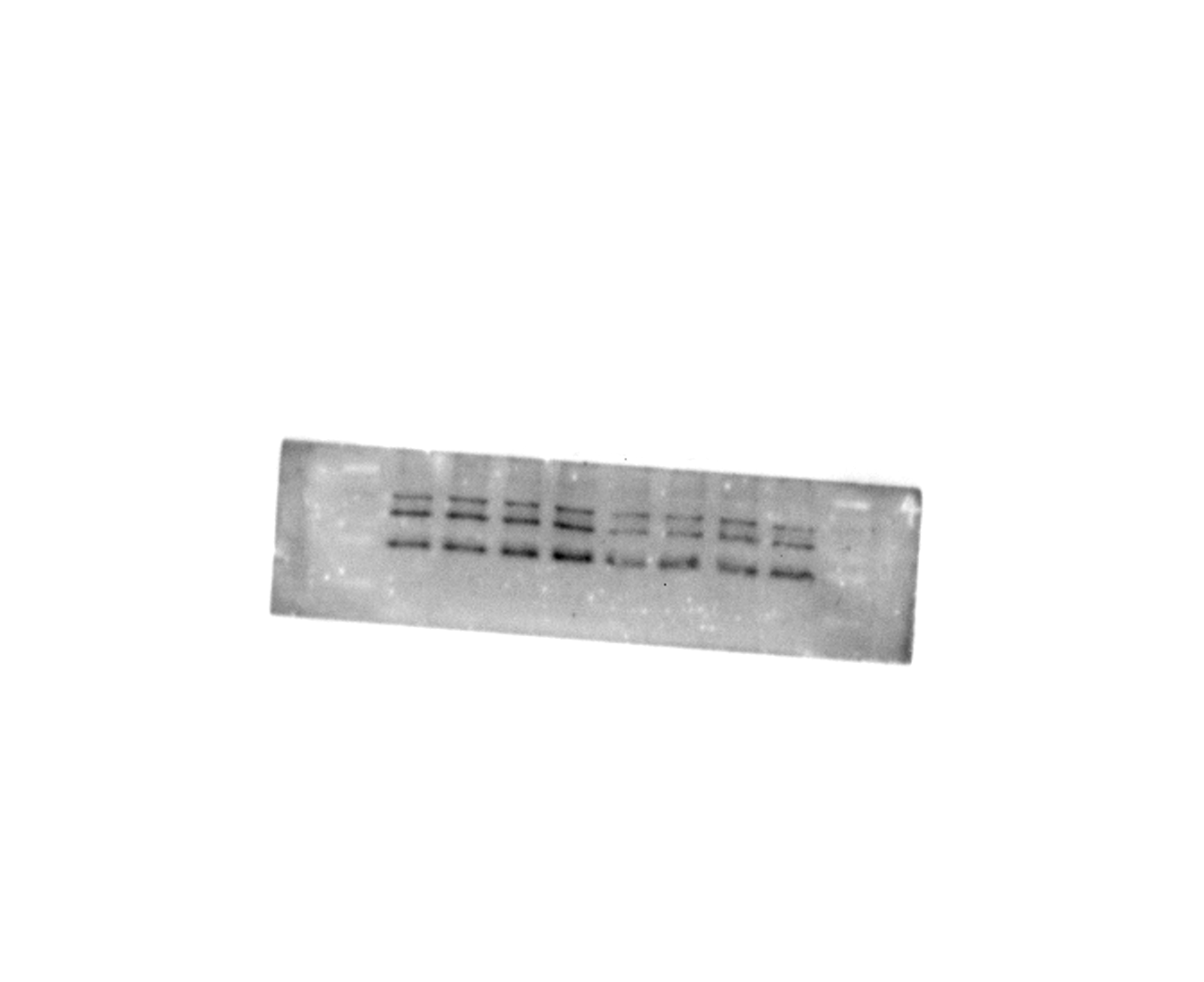

Supplement: Supplementary file 7 [file DataSheet_3.zip › figS2D-bcl2-3.Tif]

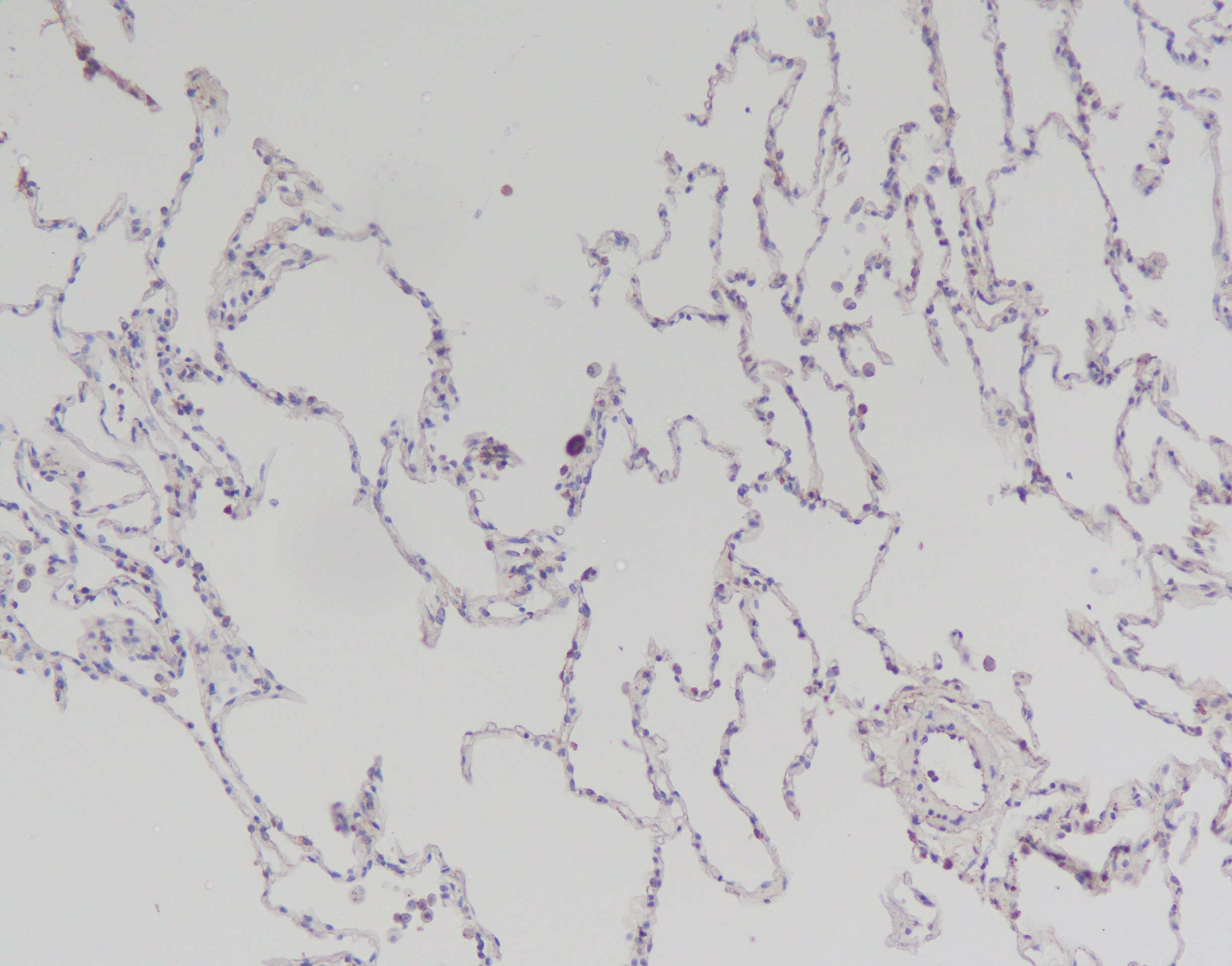

Supplement: Supplementary file 8 [file DataSheet_4.zip › 20X-CALD1-HC140.jpeg]

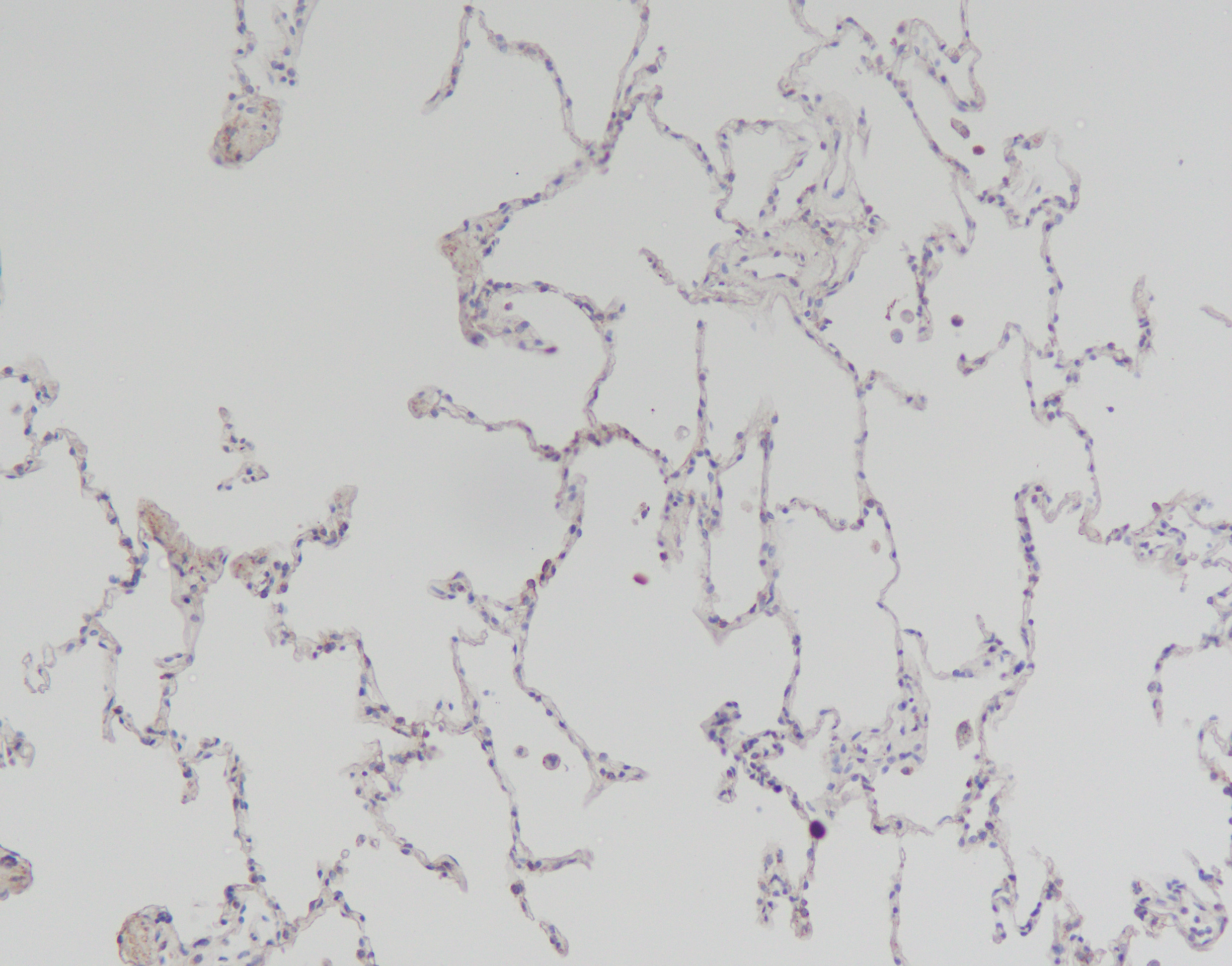

Supplement: Supplementary file 8 [file DataSheet_4.zip › 20X-CALD1-HC241.jpeg]

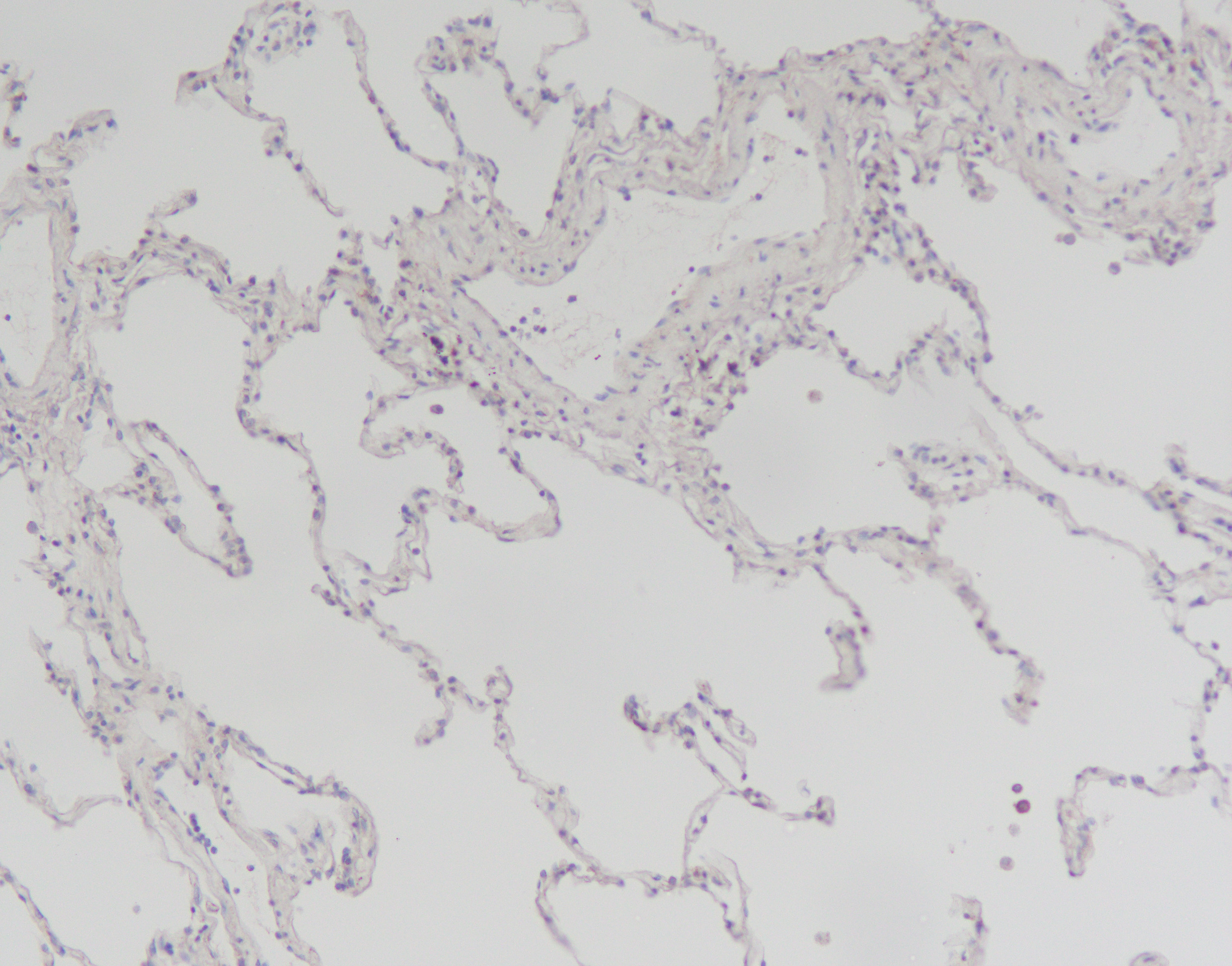

Supplement: Supplementary file 8 [file DataSheet_4.zip › 20X-CALD1-HC342.jpeg]

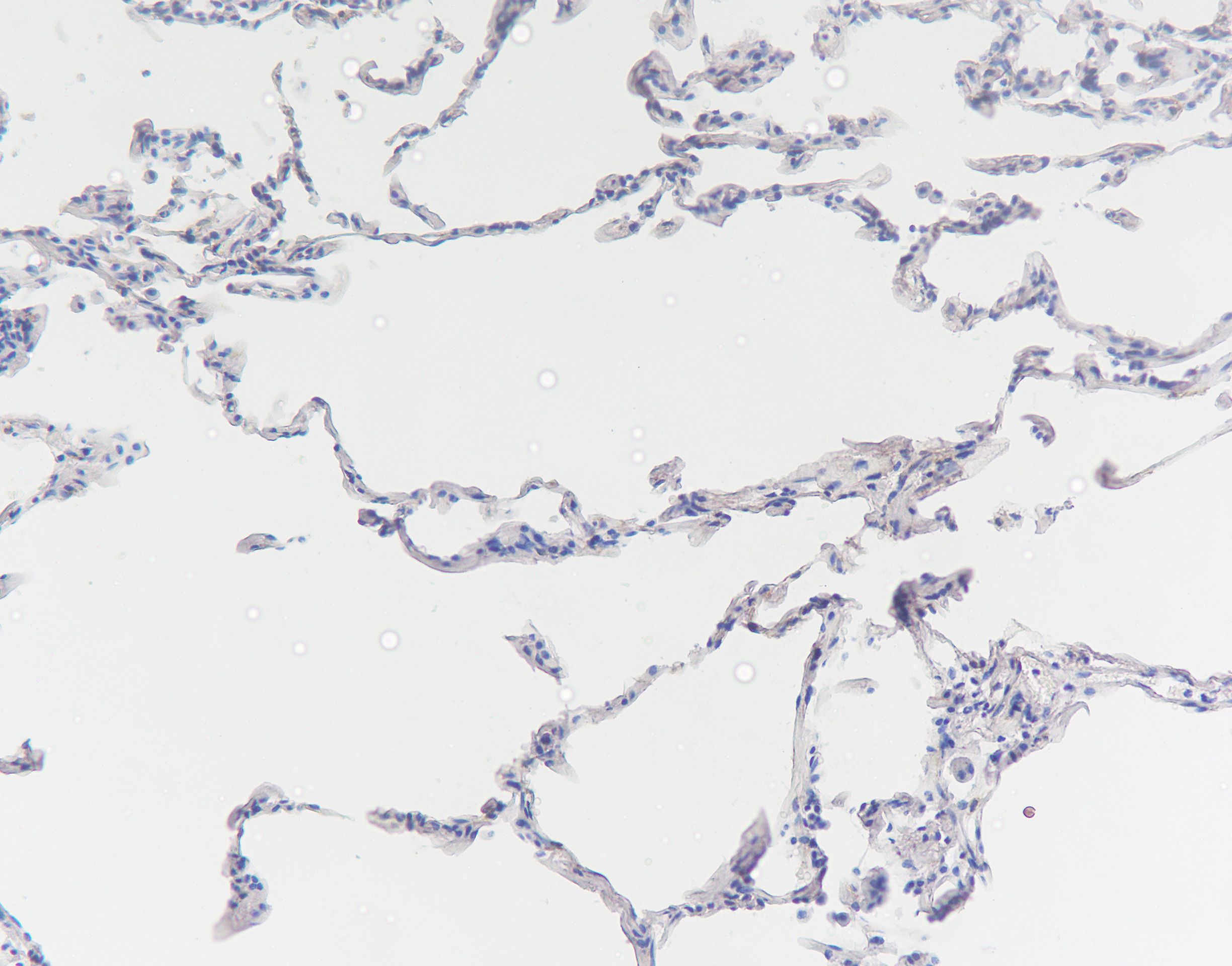

Supplement: Supplementary file 8 [file DataSheet_4.zip › 20X-CALD1-HC411.jpeg]

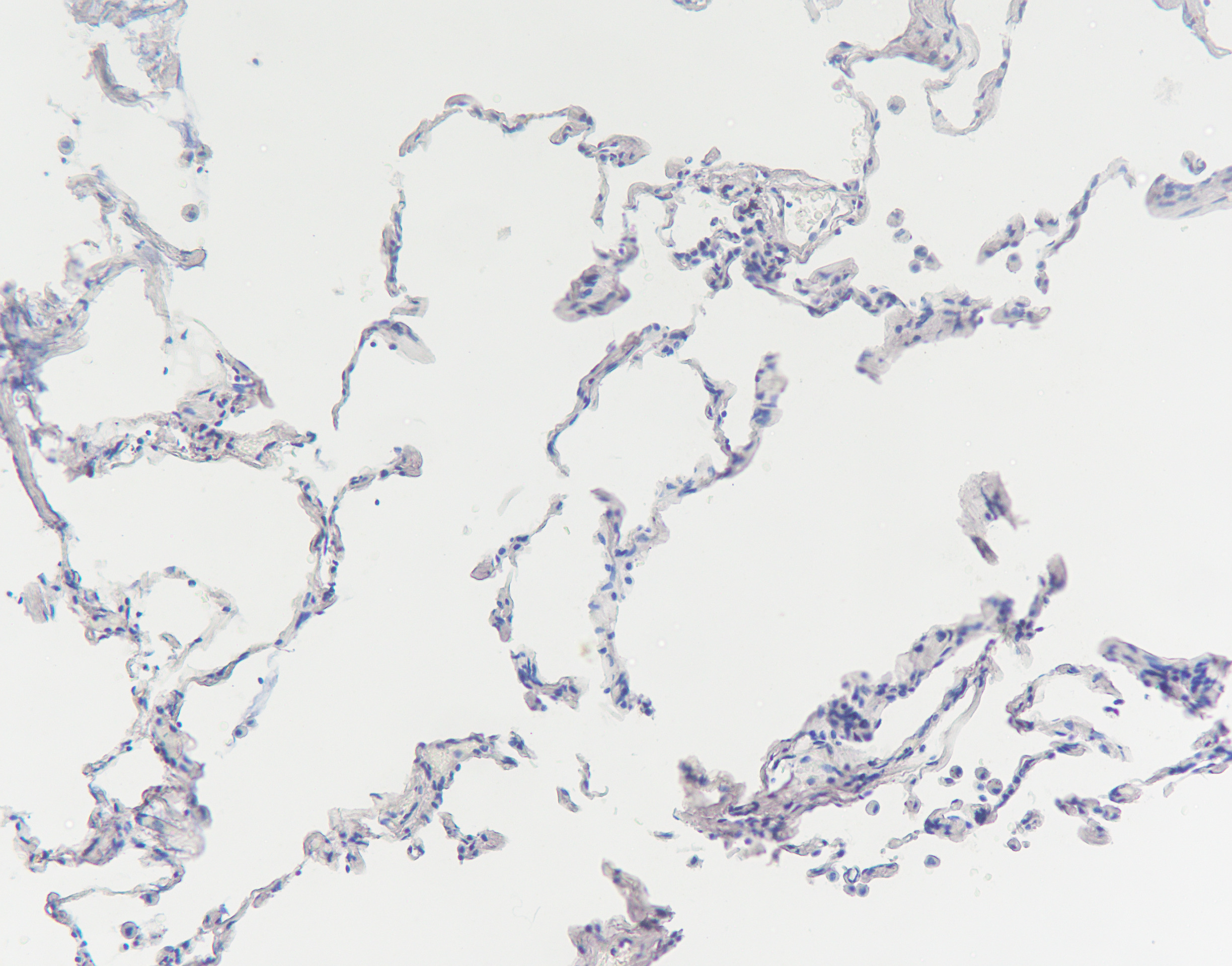

Supplement: Supplementary file 8 [file DataSheet_4.zip › 20X-CALD1-HC512.jpeg]

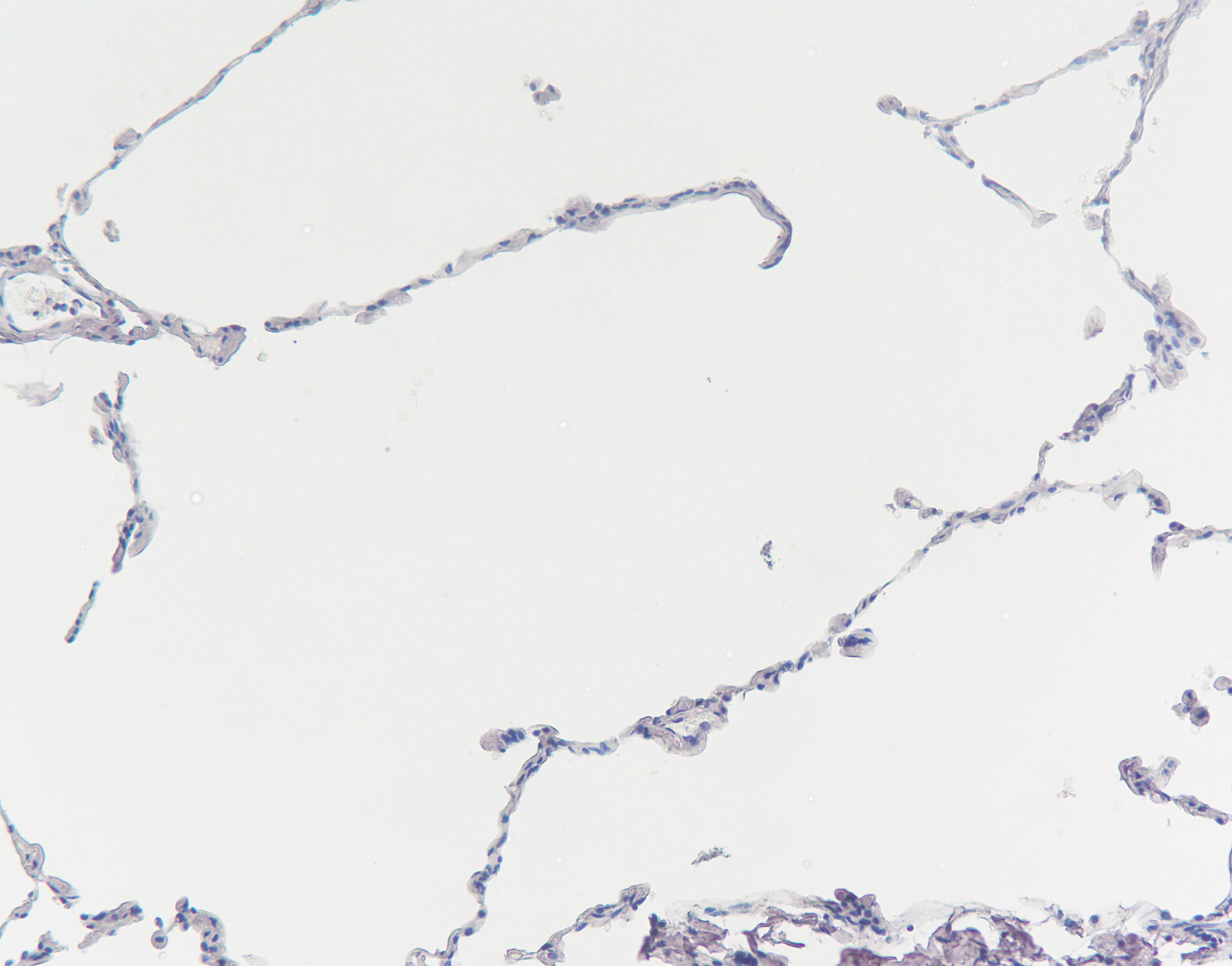

Supplement: Supplementary file 8 [file DataSheet_4.zip › 20X-CALD1-HC613.jpeg]

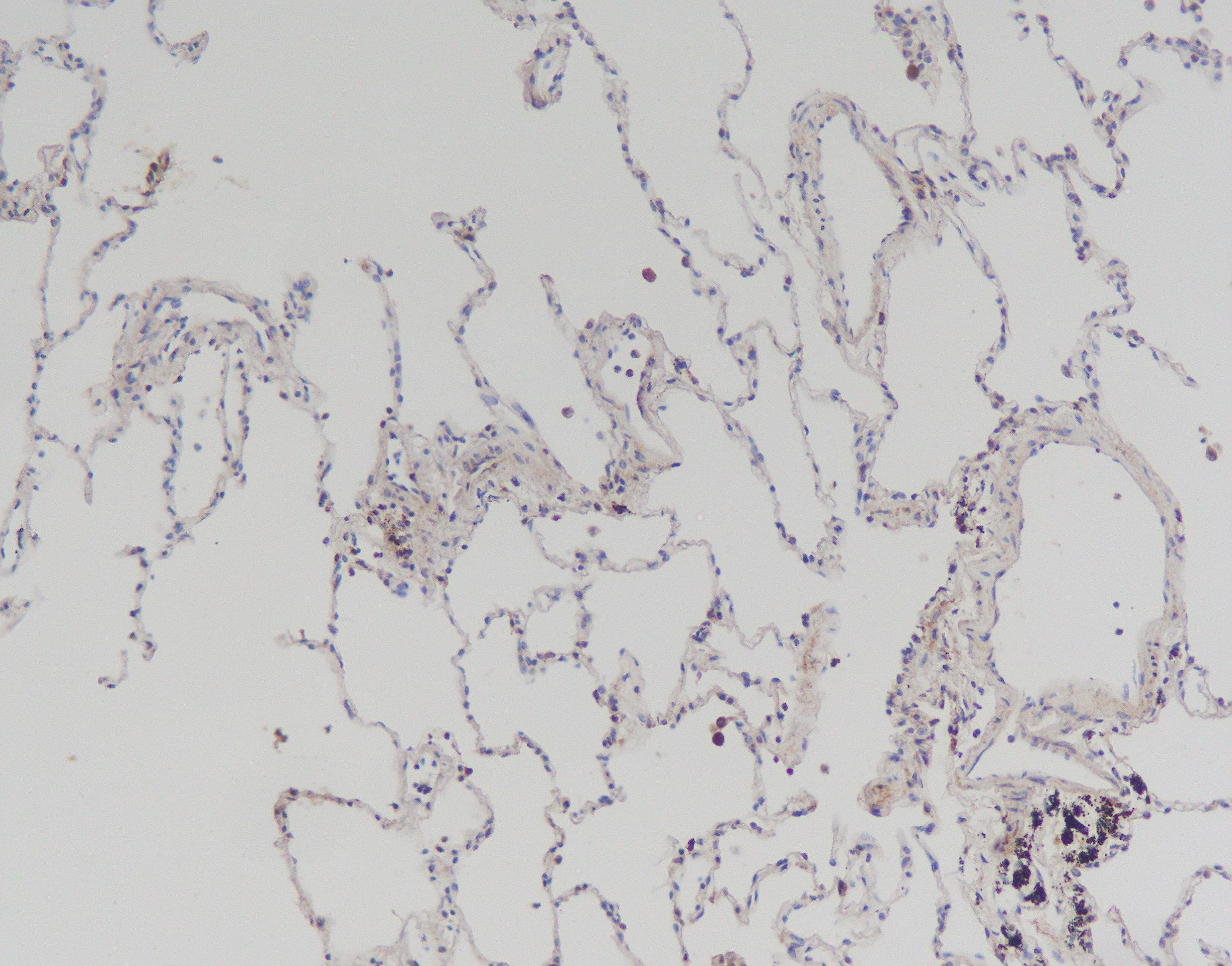

Supplement: Supplementary file 8 [file DataSheet_4.zip › 20X-CALD1-HC743.jpeg]

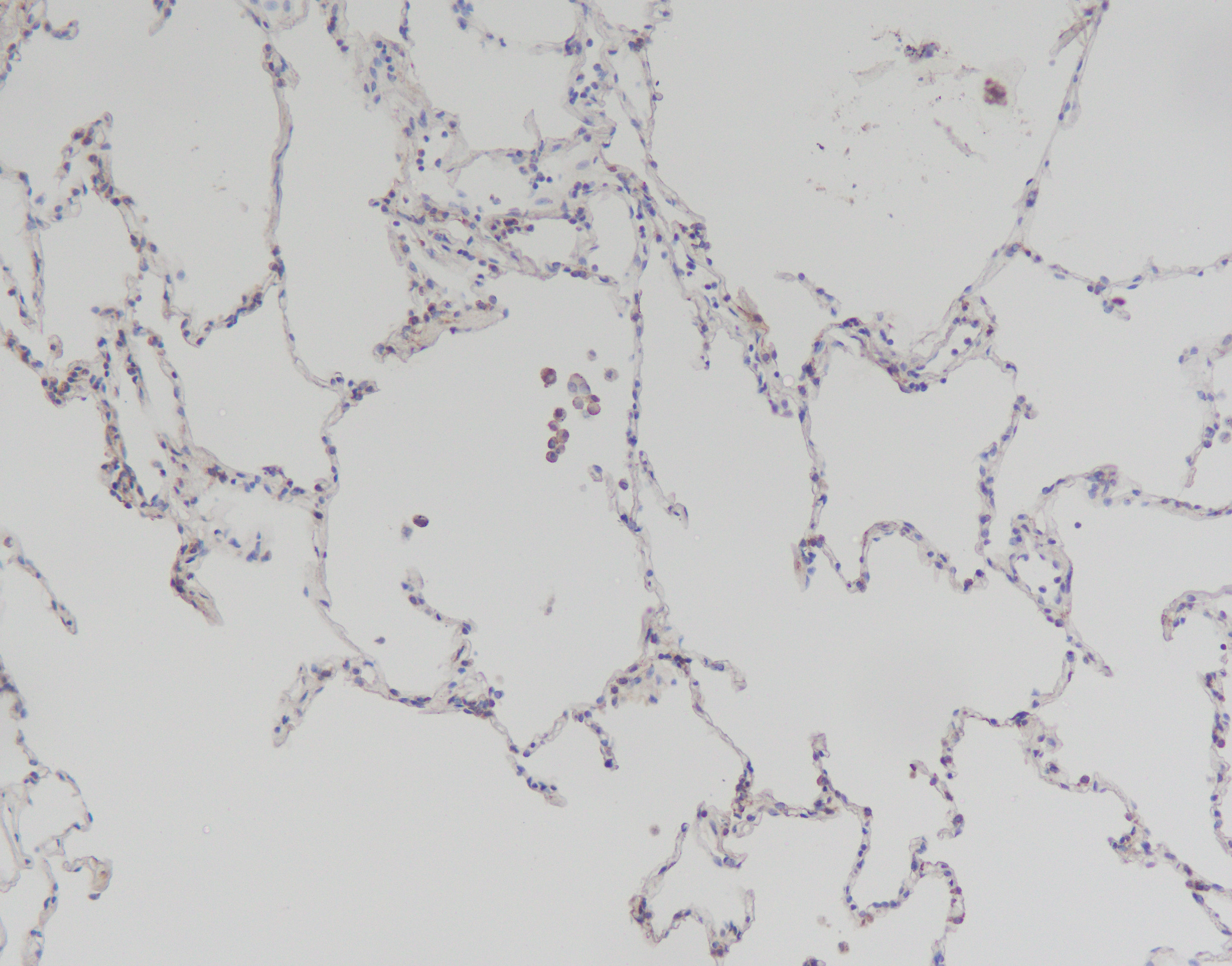

Supplement: Supplementary file 8 [file DataSheet_4.zip › 20X-CALD1-HC844.jpeg]

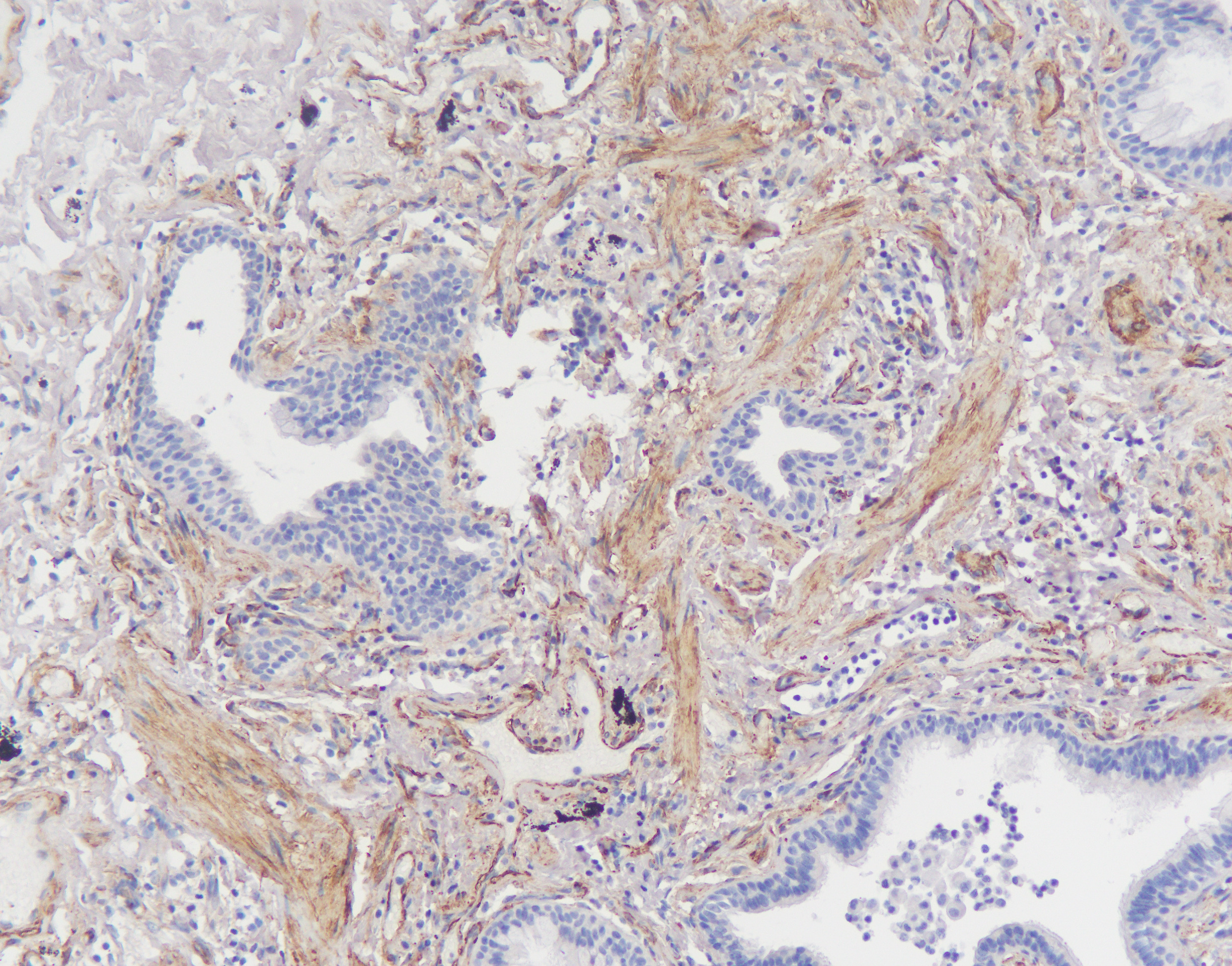

Supplement: Supplementary file 8 [file DataSheet_4.zip › 20X-CALD1-IPF145.jpeg]

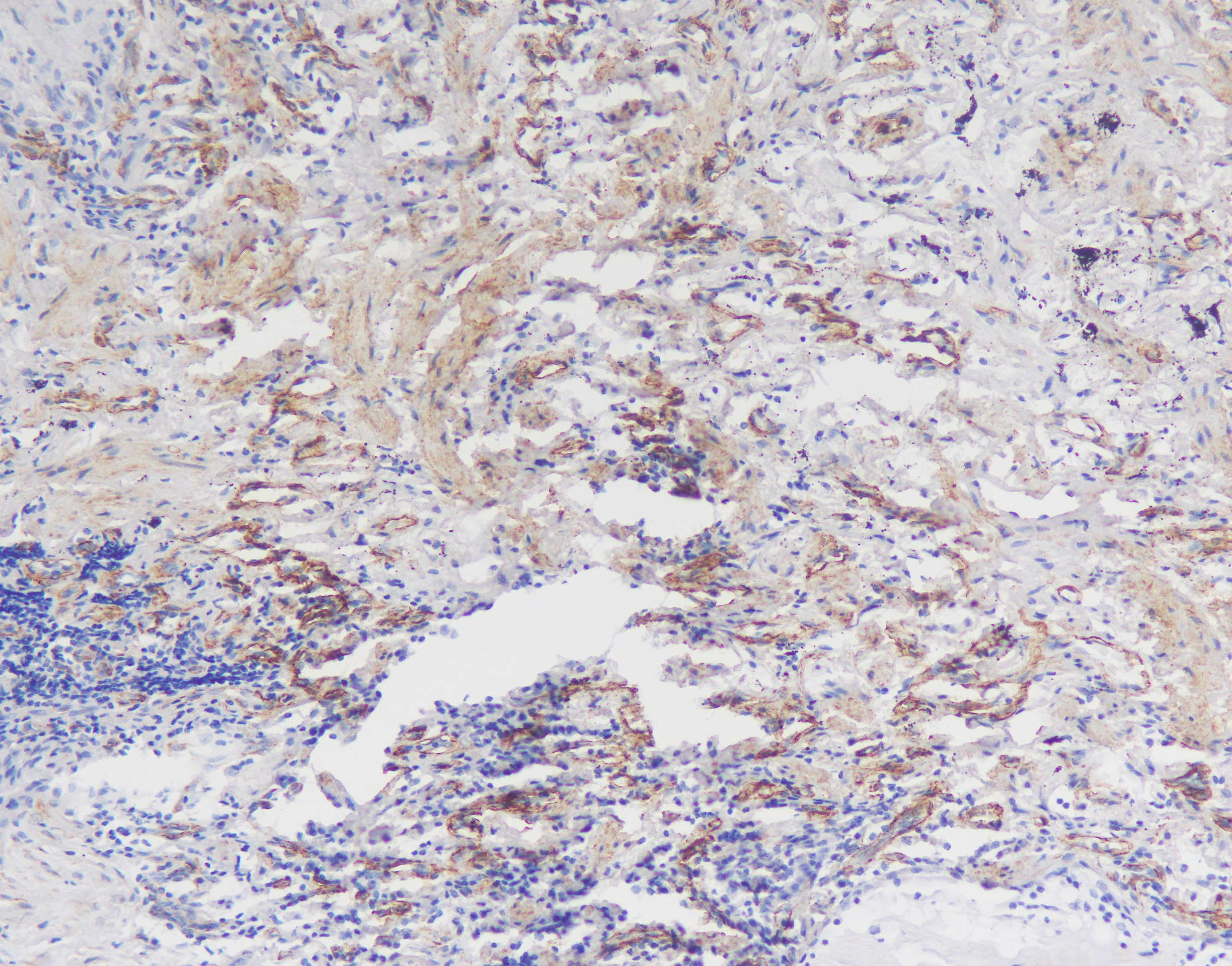

Supplement: Supplementary file 8 [file DataSheet_4.zip › 20X-CALD1-IPF246.jpeg]

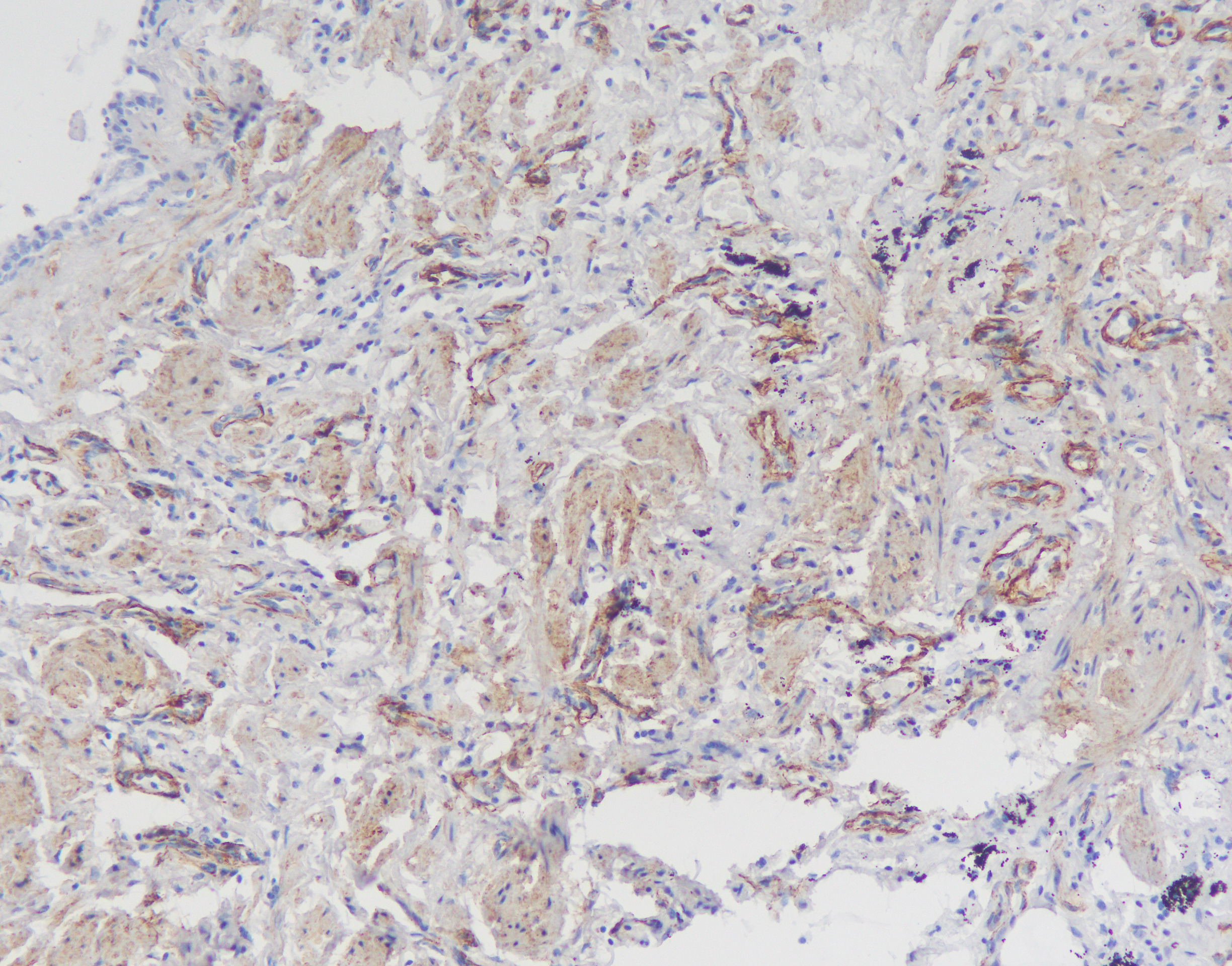

Supplement: Supplementary file 8 [file DataSheet_4.zip › 20X-CALD1-IPF347.jpeg]

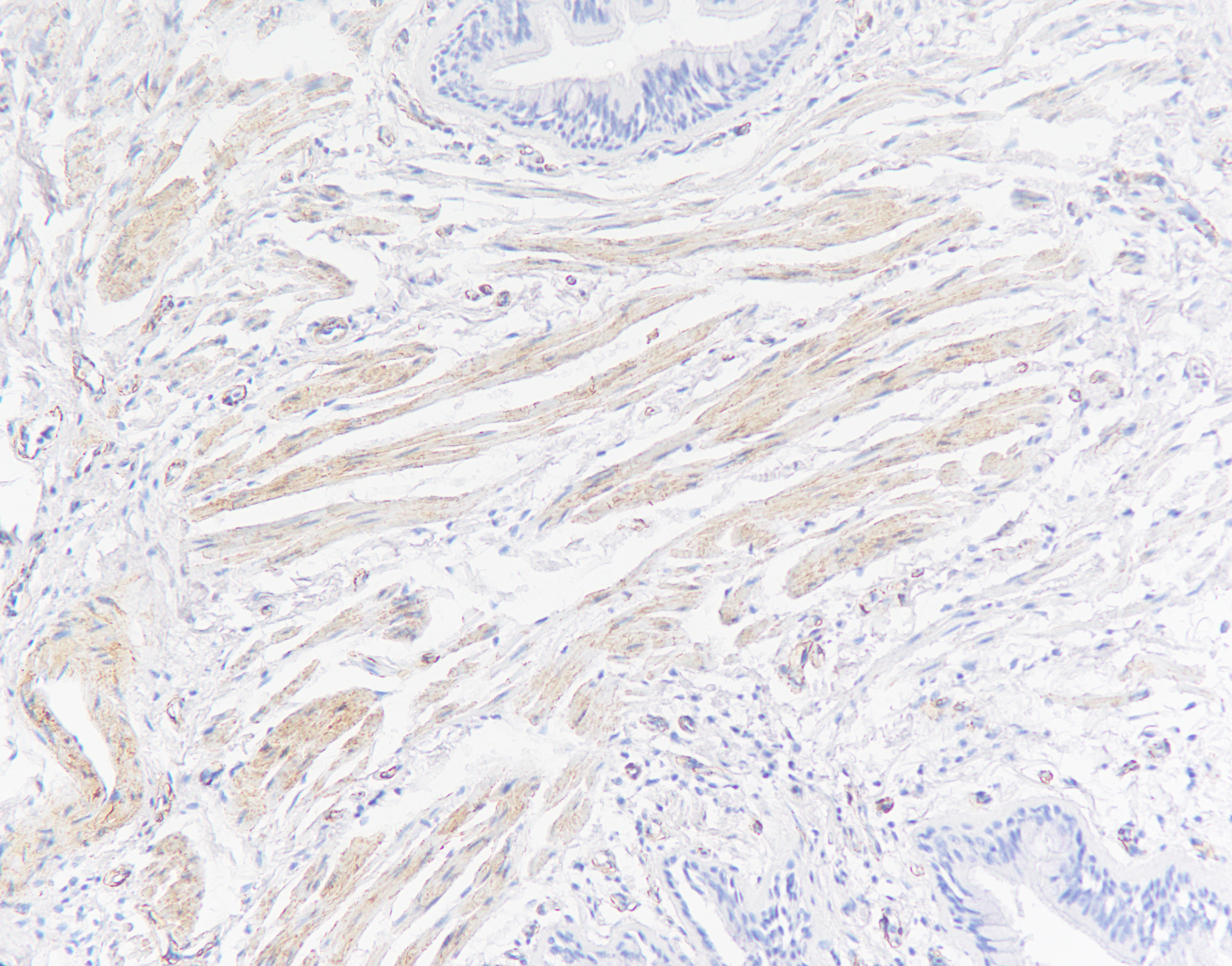

Supplement: Supplementary file 8 [file DataSheet_4.zip › 20X-CALD1-IPF414.jpeg]

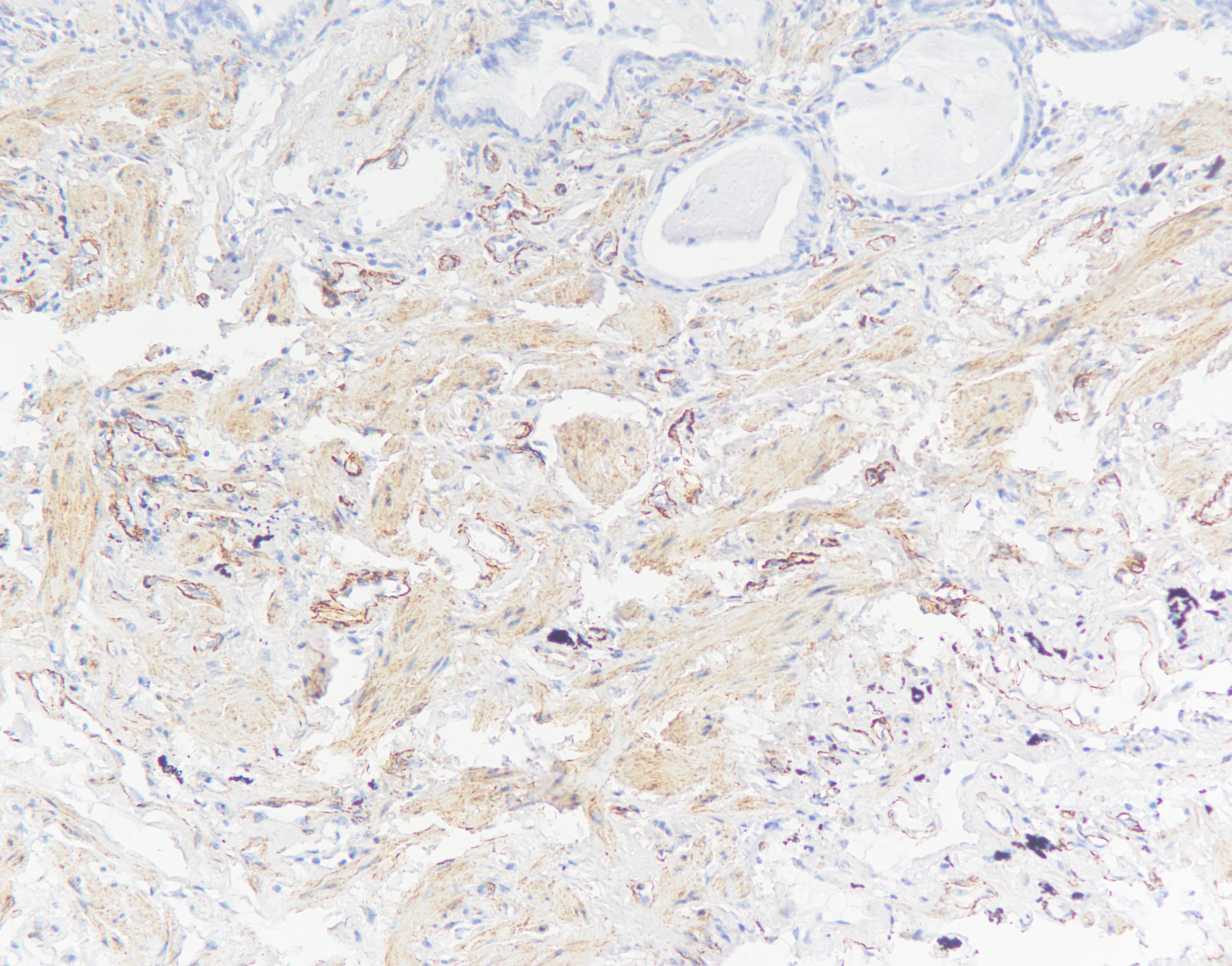

Supplement: Supplementary file 8 [file DataSheet_4.zip › 20X-CALD1-IPF515.jpeg]

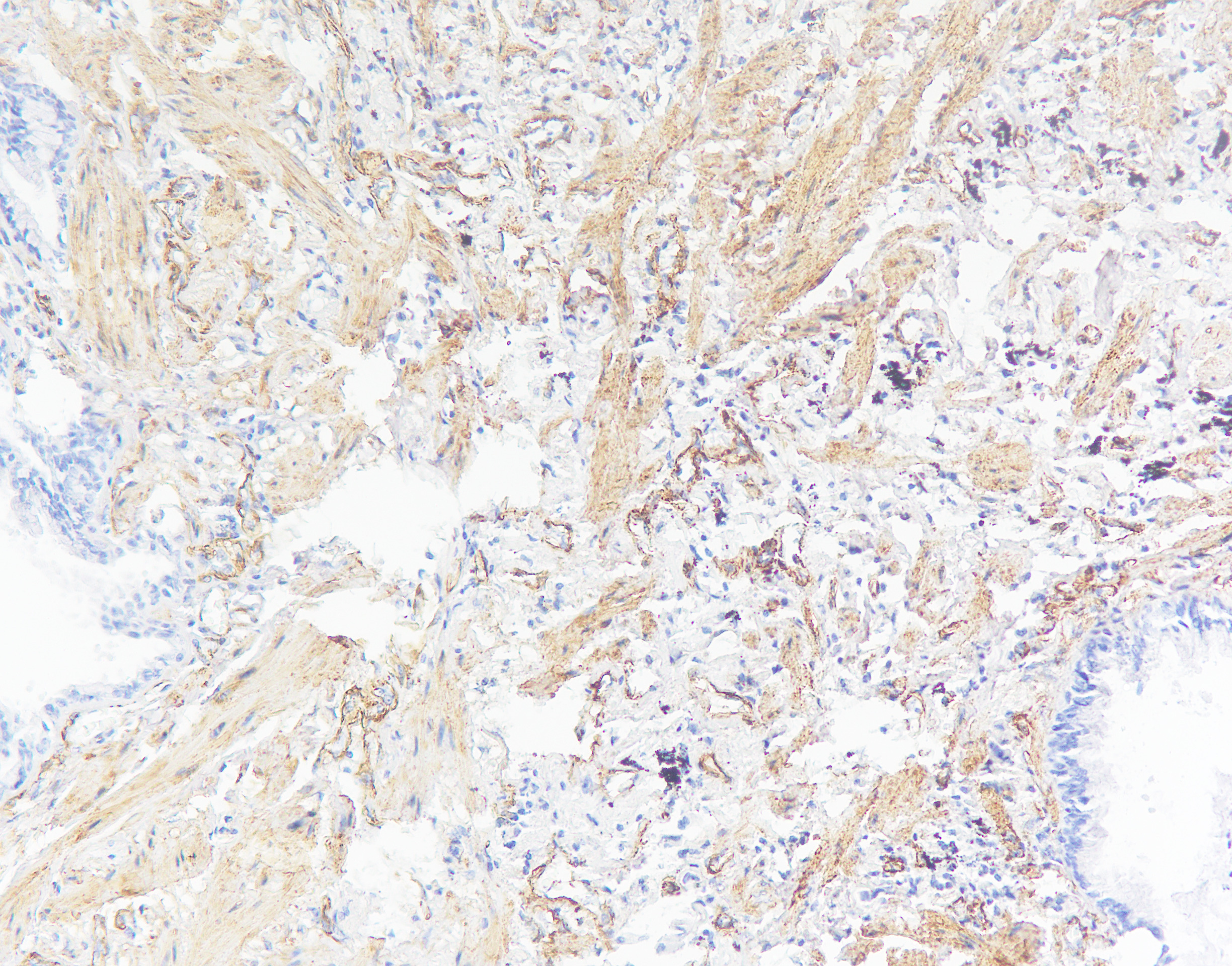

Supplement: Supplementary file 8 [file DataSheet_4.zip › 20X-CALD1-IPF616.jpeg]

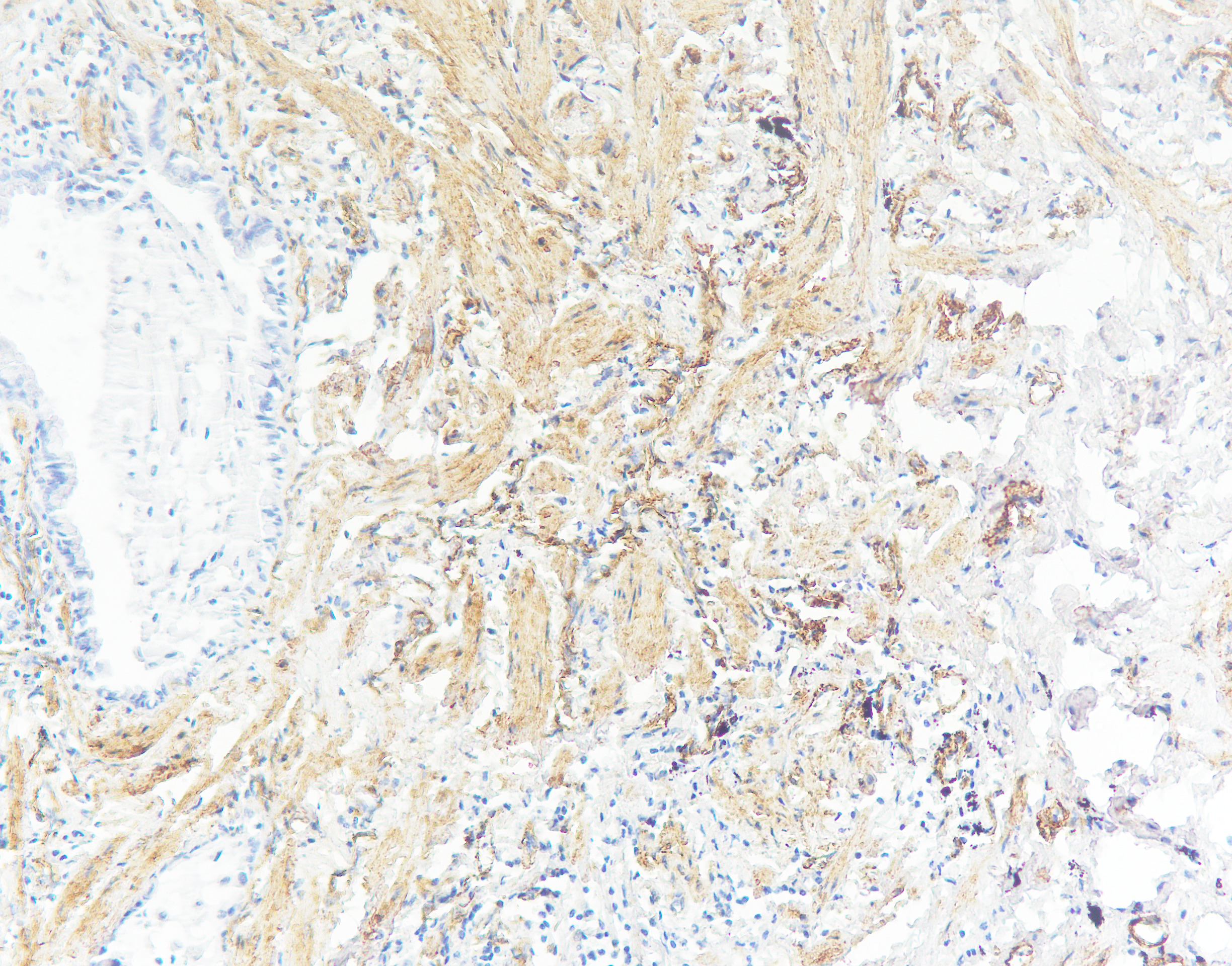

Supplement: Supplementary file 8 [file DataSheet_4.zip › 20X-CALD1-IPF717.jpeg]

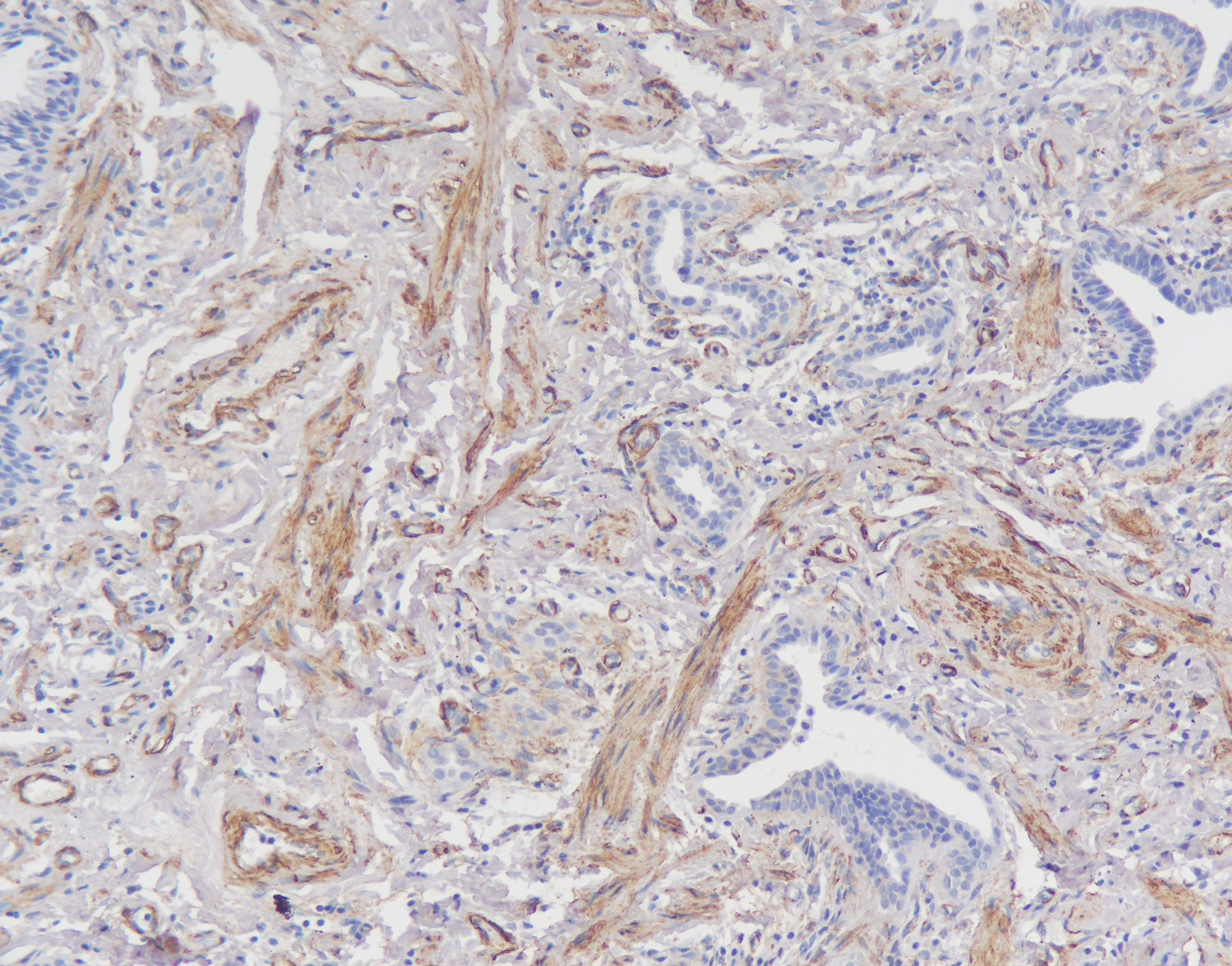

Supplement: Supplementary file 8 [file DataSheet_4.zip › 20X-CALD1-IPF848.jpeg]

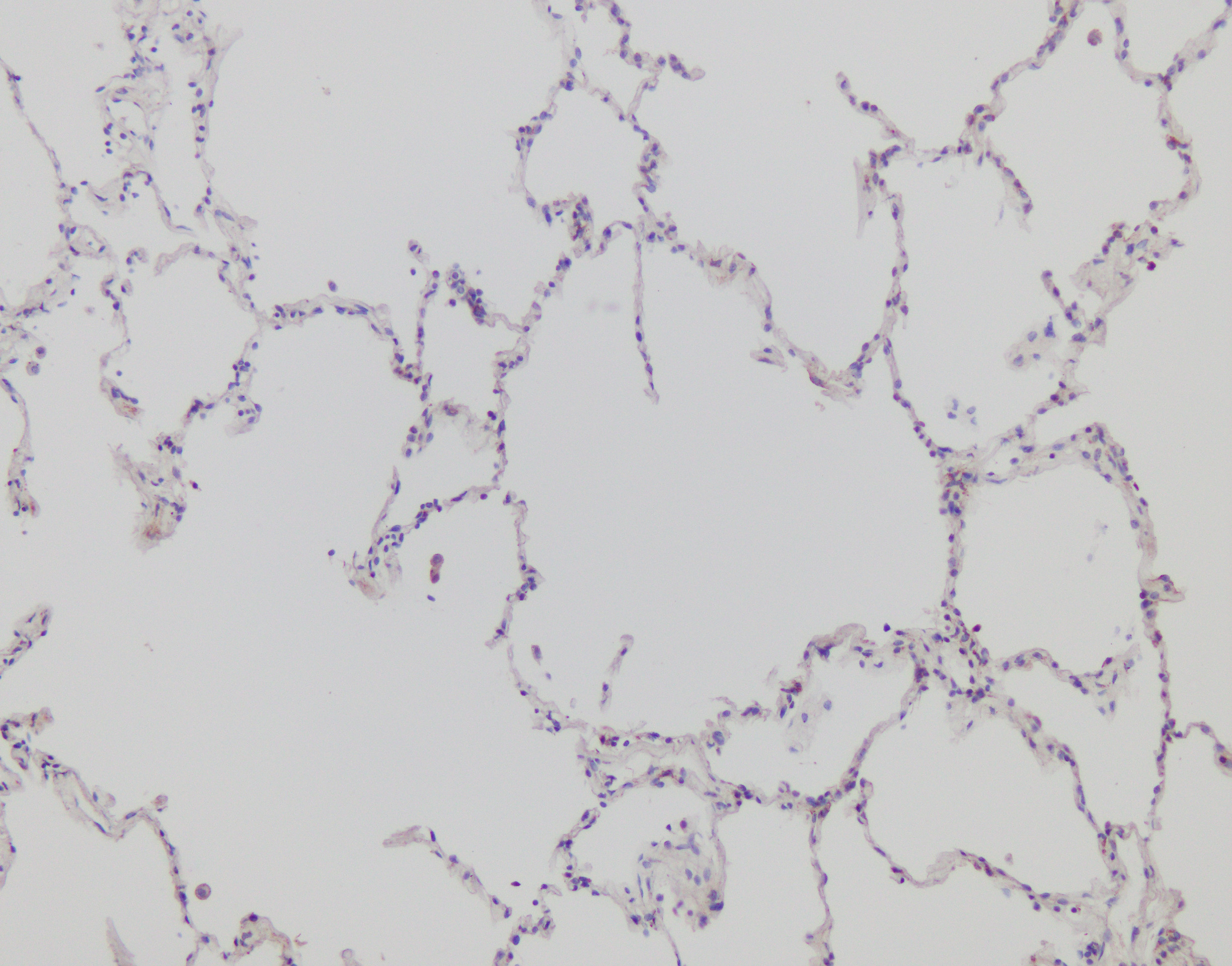

Supplement: Supplementary file 9 [file DataSheet_5.zip › 20X-CDH2-HC19.jpeg]

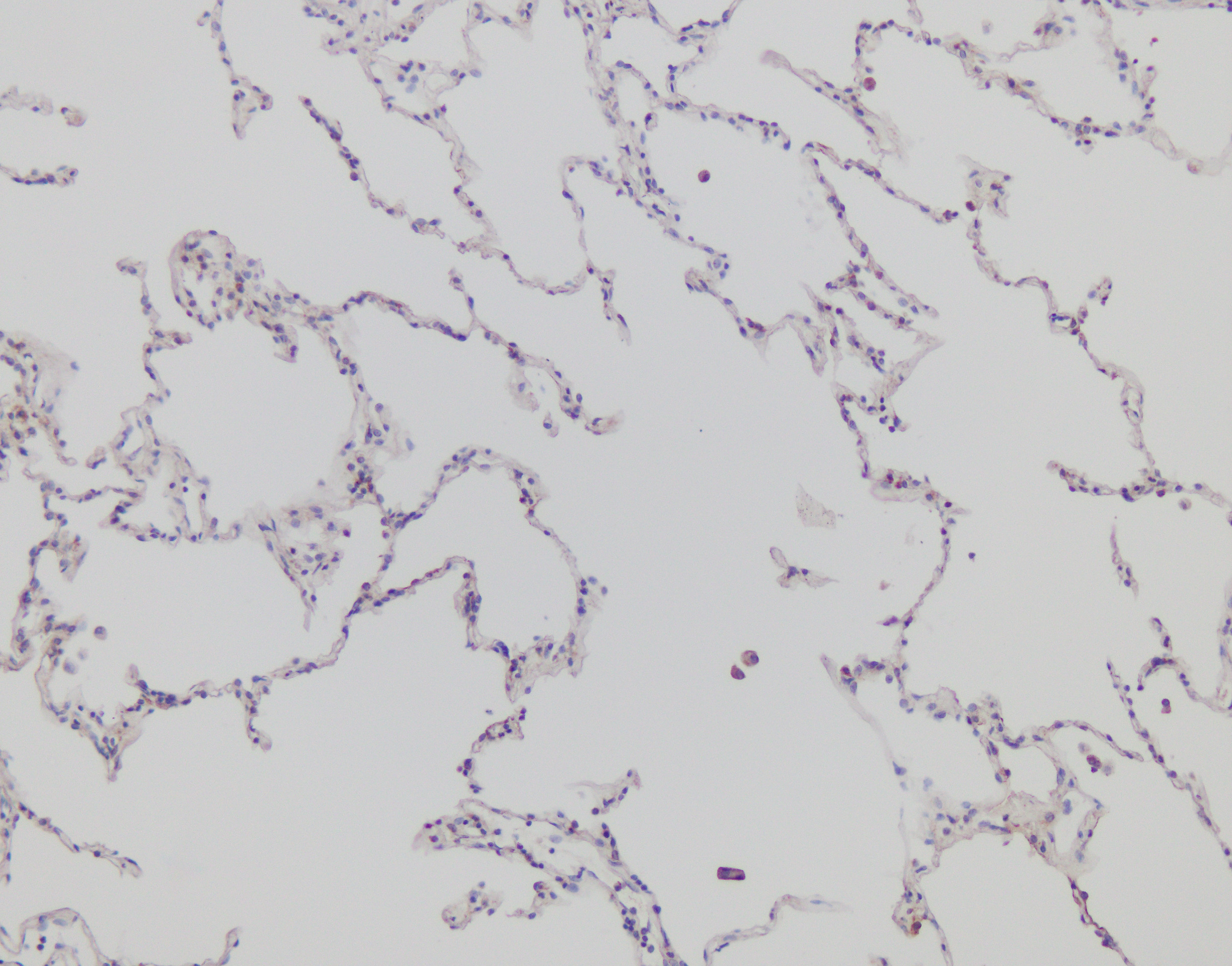

Supplement: Supplementary file 9 [file DataSheet_5.zip › 20X-CDH2-HC210.jpeg]

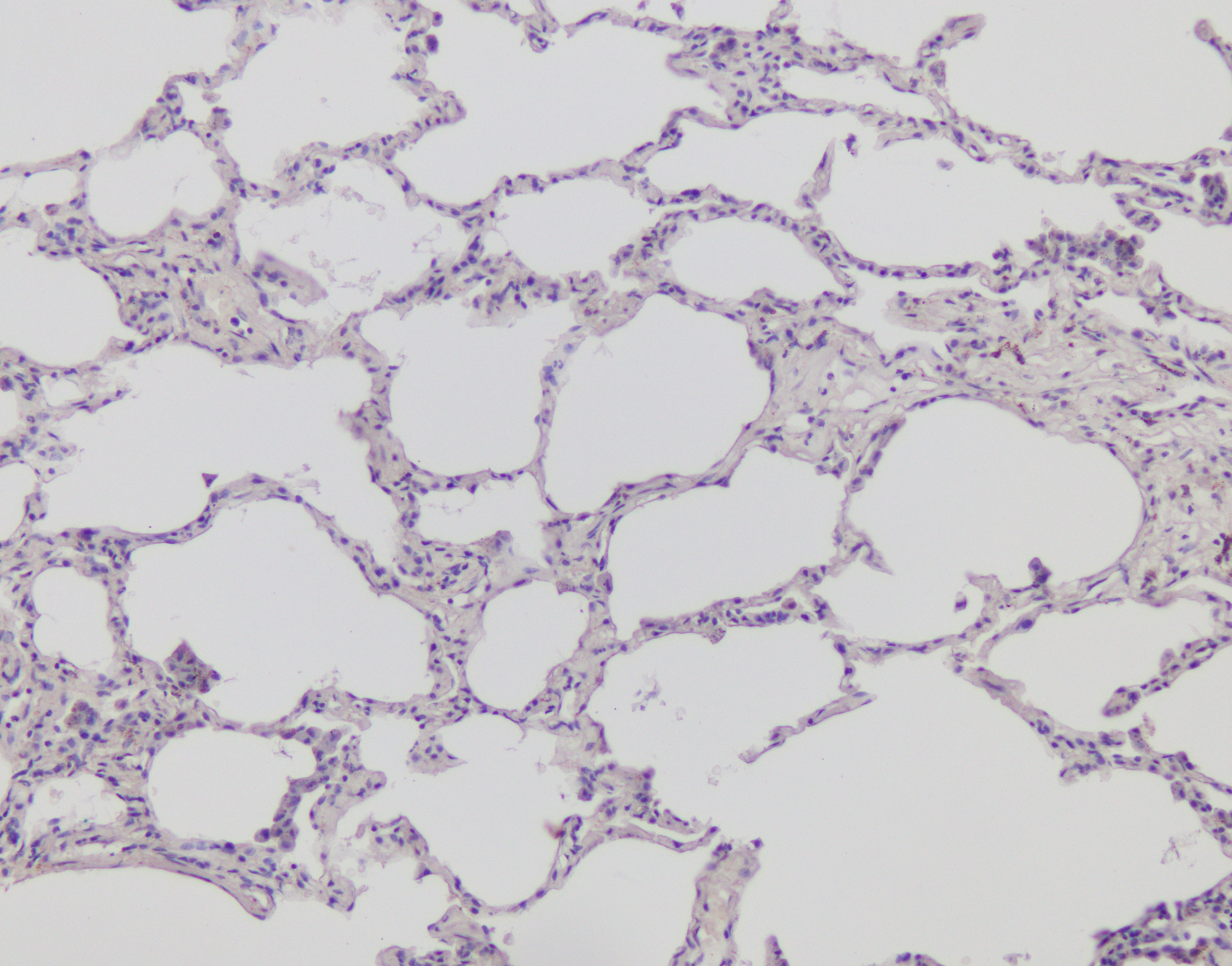

Supplement: Supplementary file 9 [file DataSheet_5.zip › 20X-CDH2-HC35.jpeg]

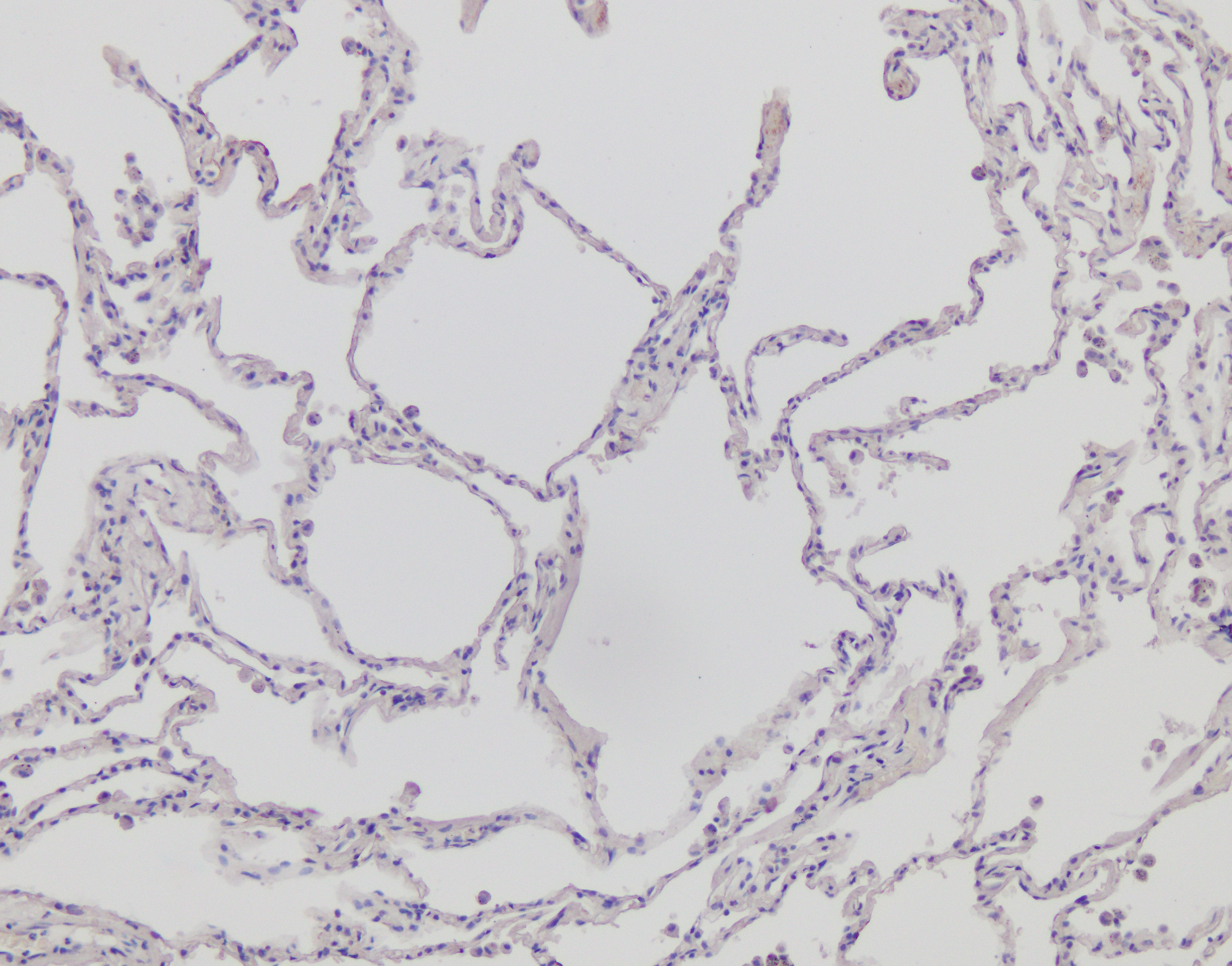

Supplement: Supplementary file 9 [file DataSheet_5.zip › 20X-CDH2-HC46.jpeg]

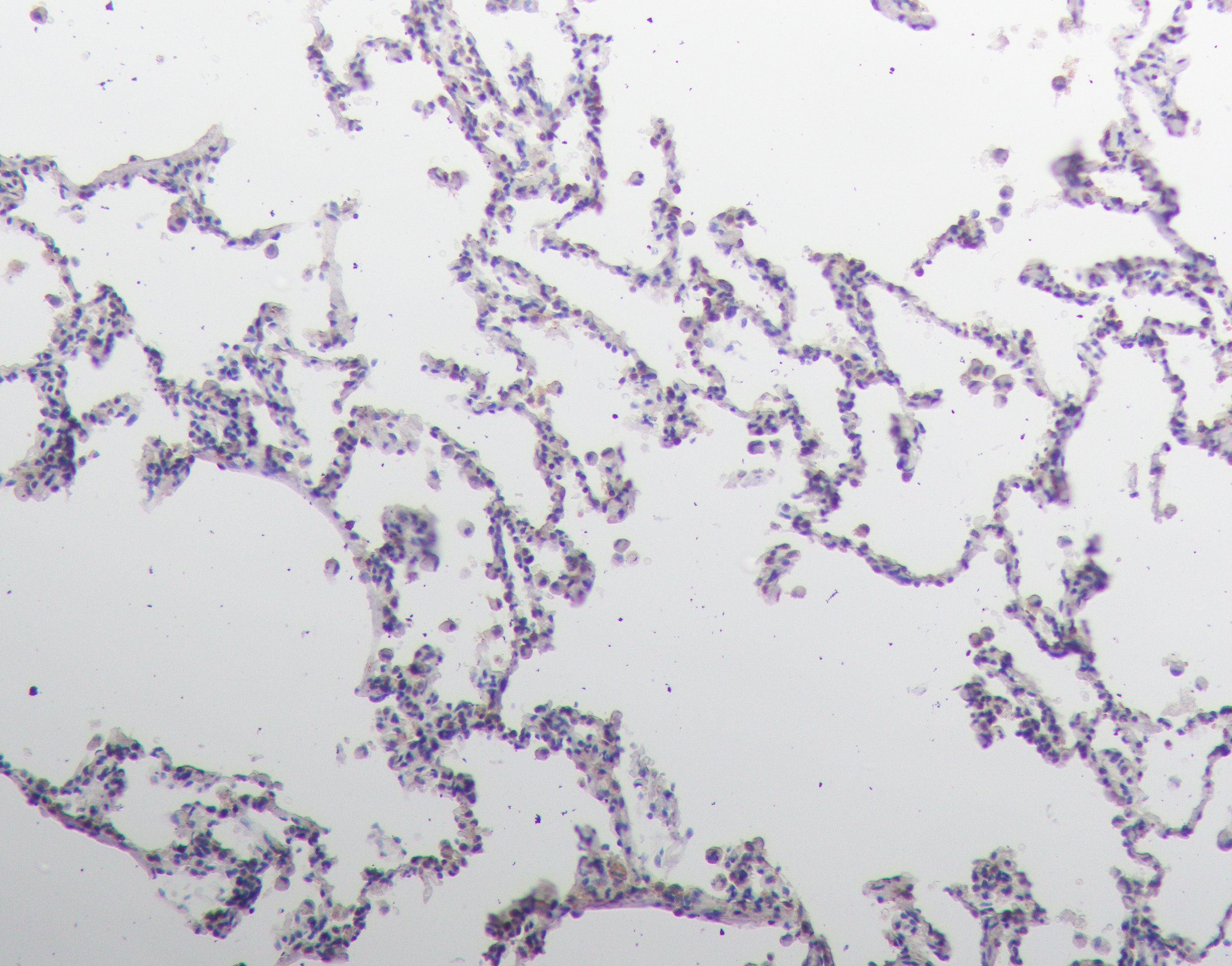

Supplement: Supplementary file 9 [file DataSheet_5.zip › 20X-CDH2-HC525.jpeg]

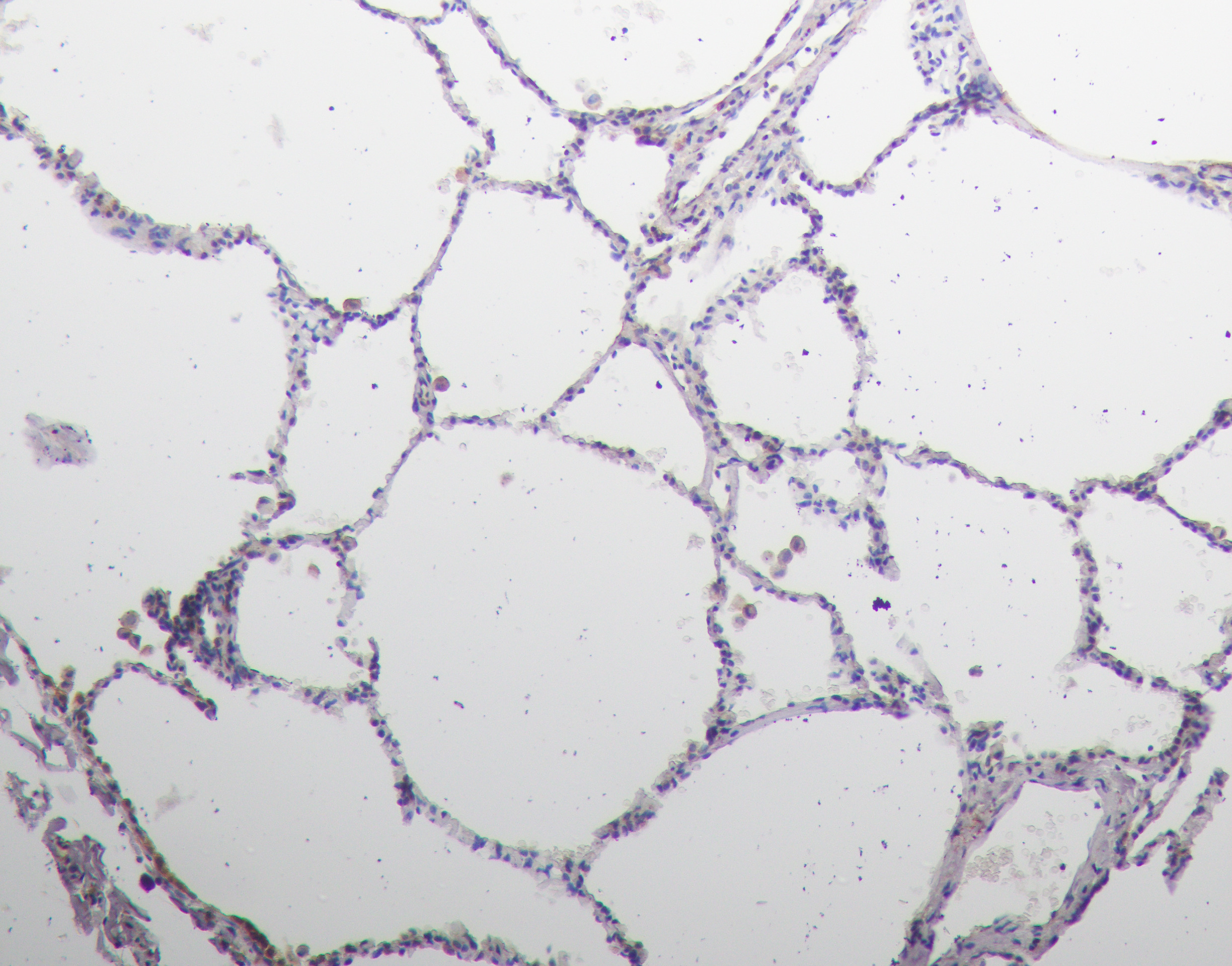

Supplement: Supplementary file 9 [file DataSheet_5.zip › 20X-CDH2-HC626.jpeg]

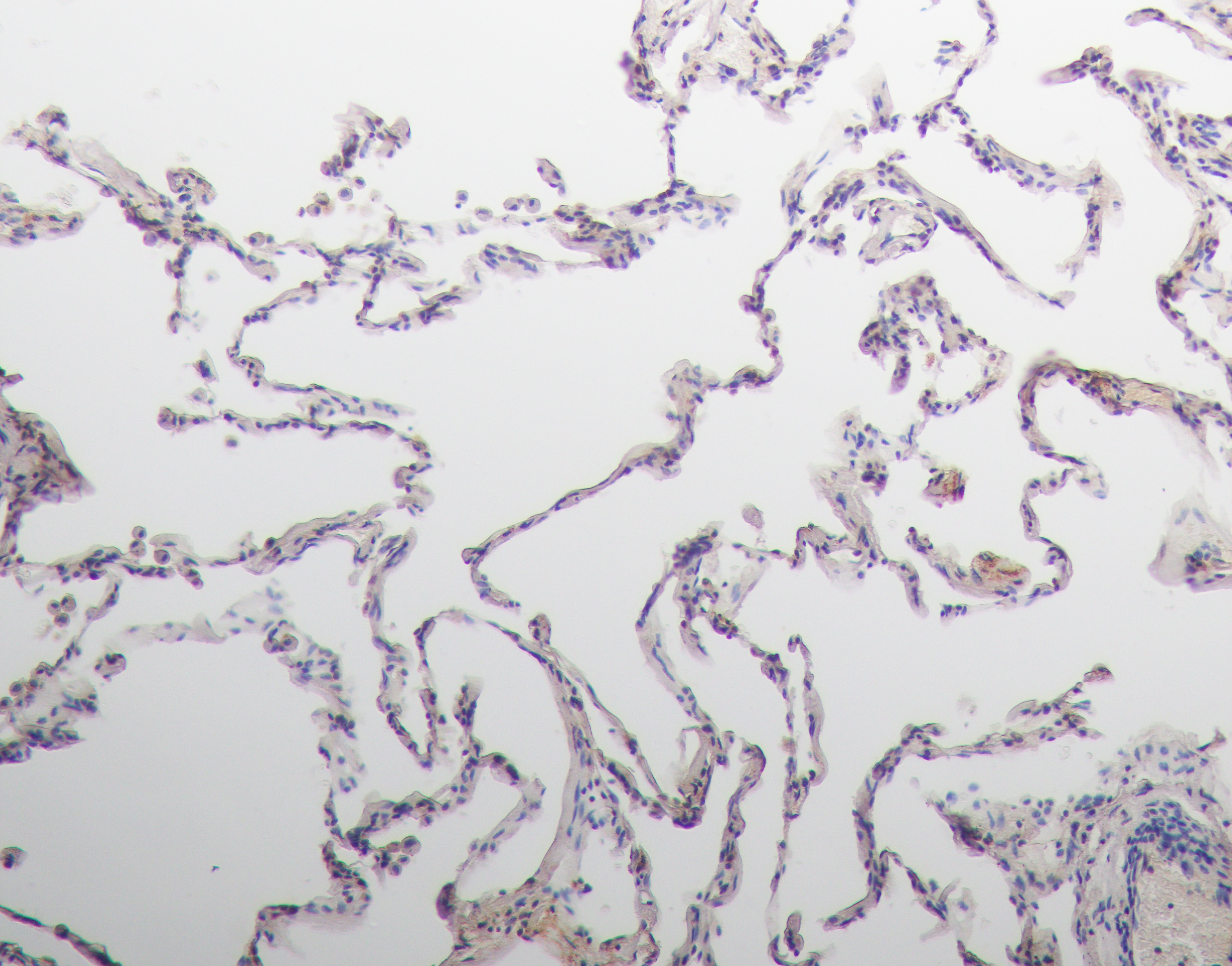

Supplement: Supplementary file 9 [file DataSheet_5.zip › 20X-CDH2-HC727.jpeg]

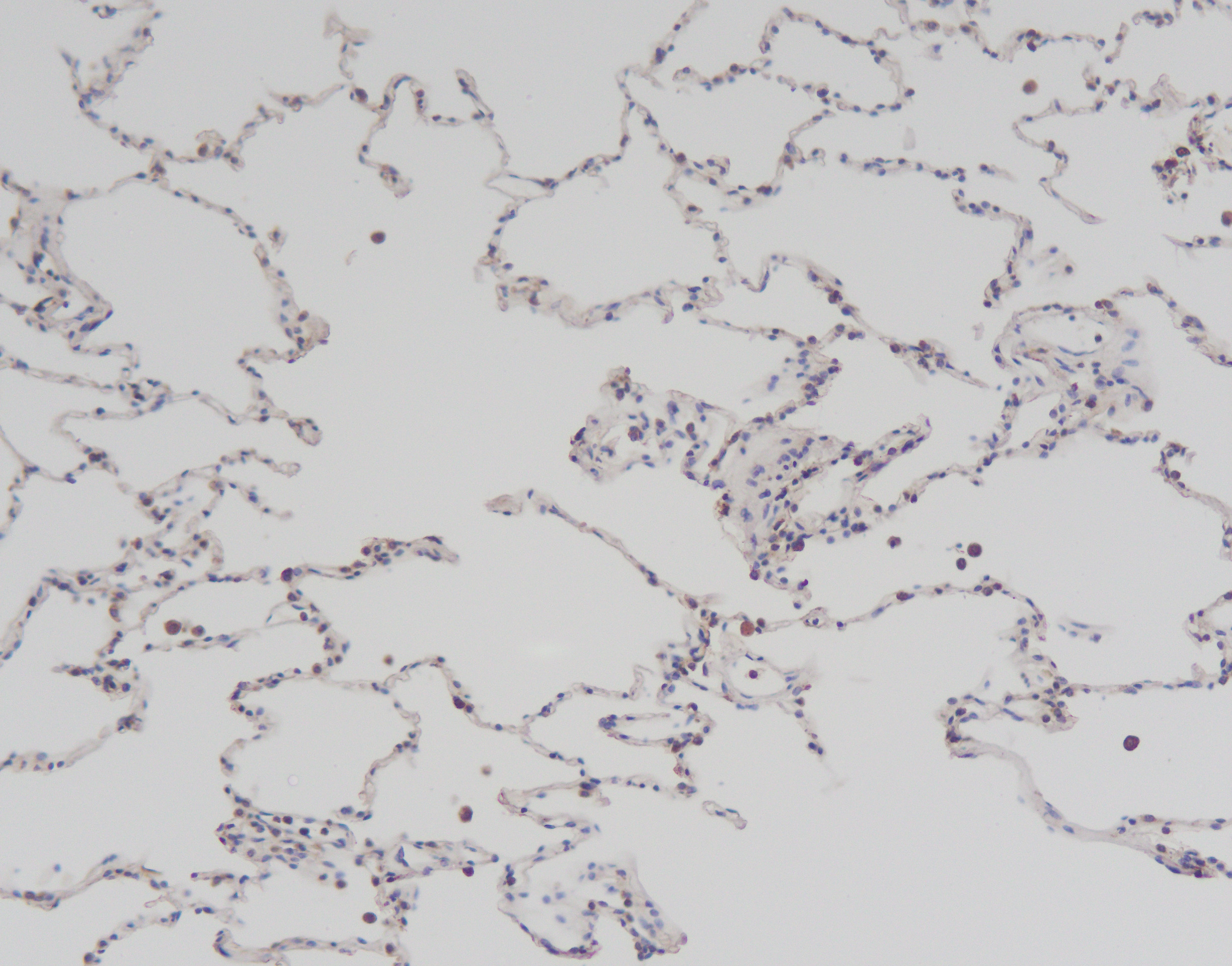

Supplement: Supplementary file 9 [file DataSheet_5.zip › 20X-CDH2-HC823.jpeg]

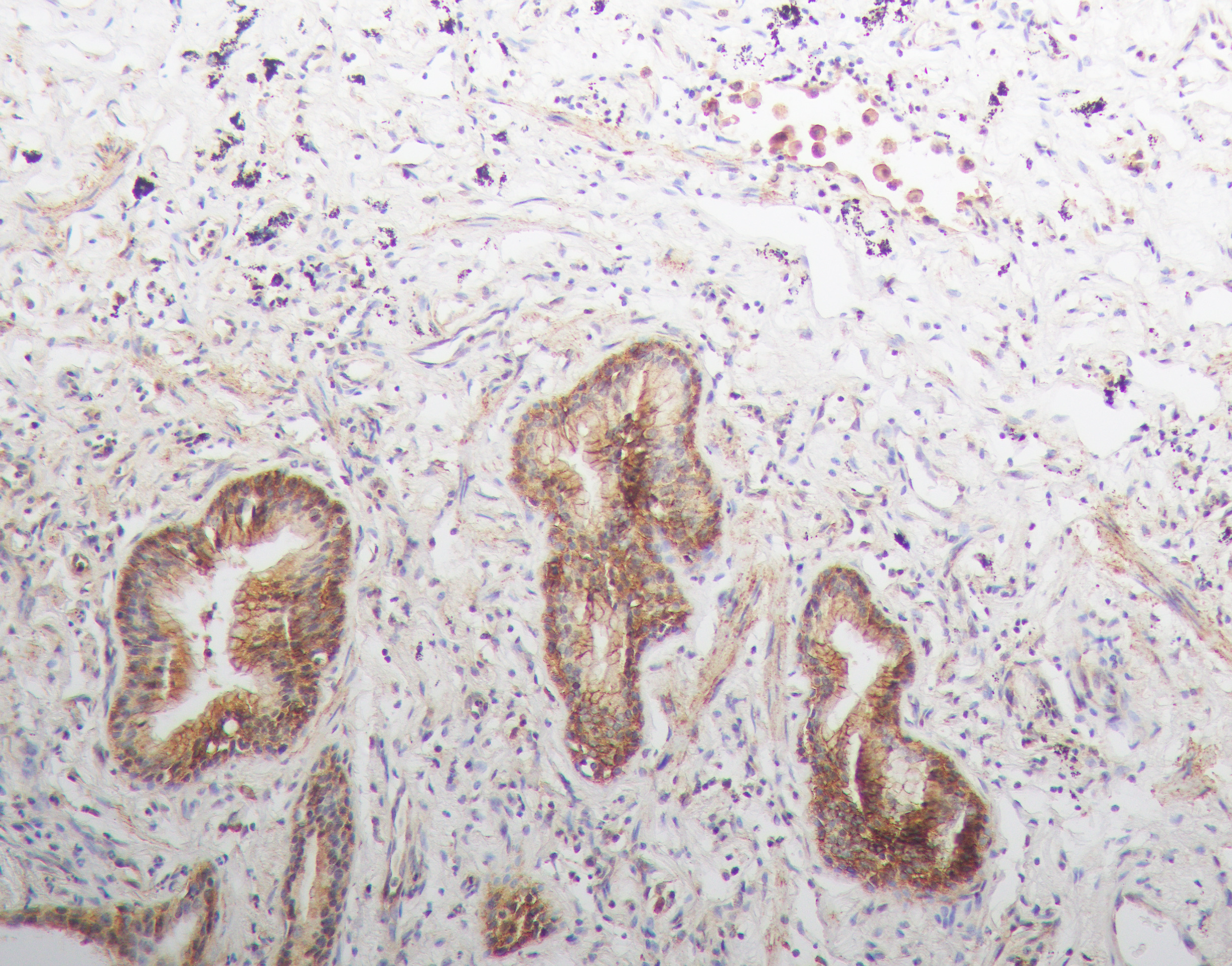

Supplement: Supplementary file 9 [file DataSheet_5.zip › 20X-CDH2-IPF14.jpeg]

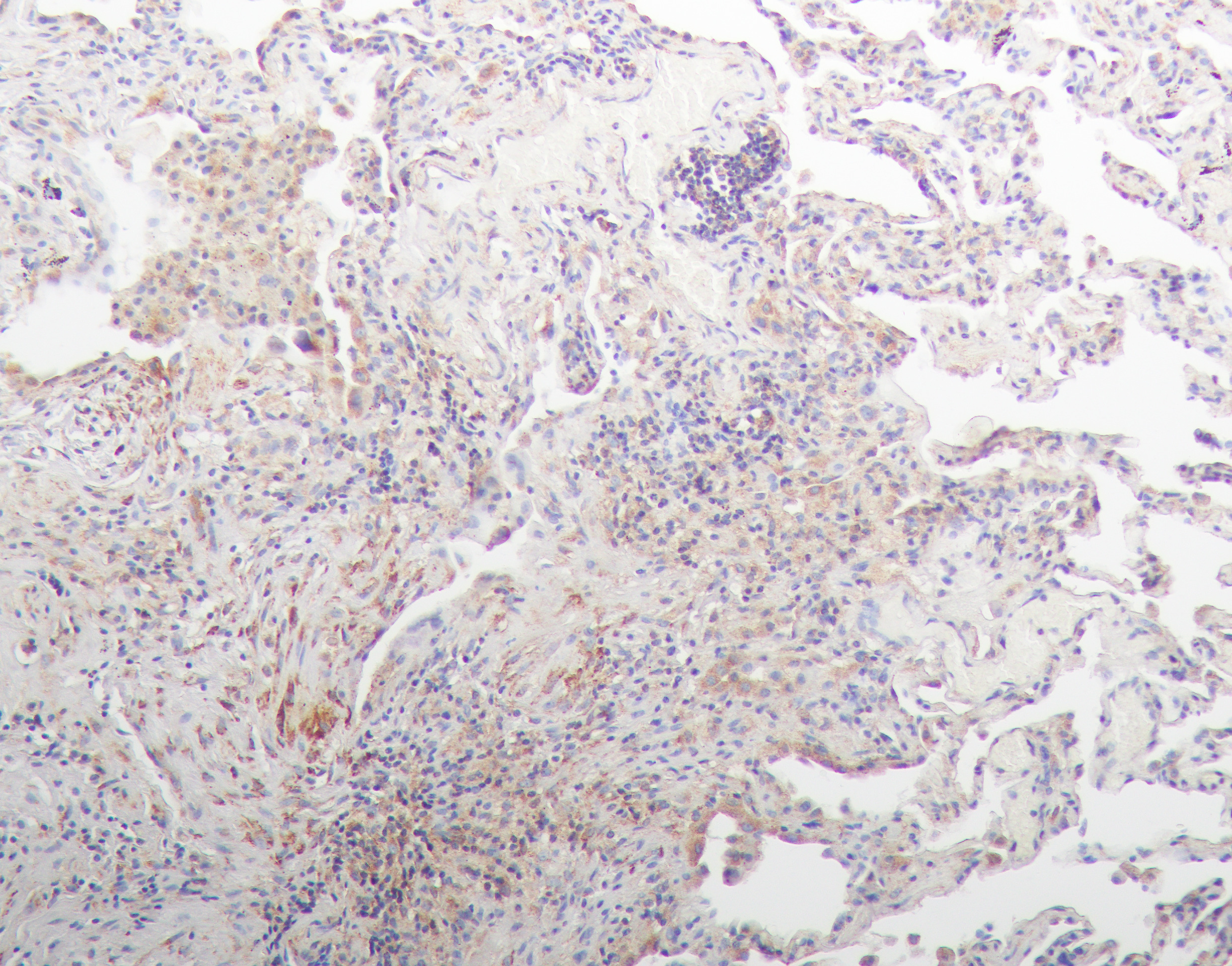

Supplement: Supplementary file 9 [file DataSheet_5.zip › 20X-CDH2-IPF23.jpeg]

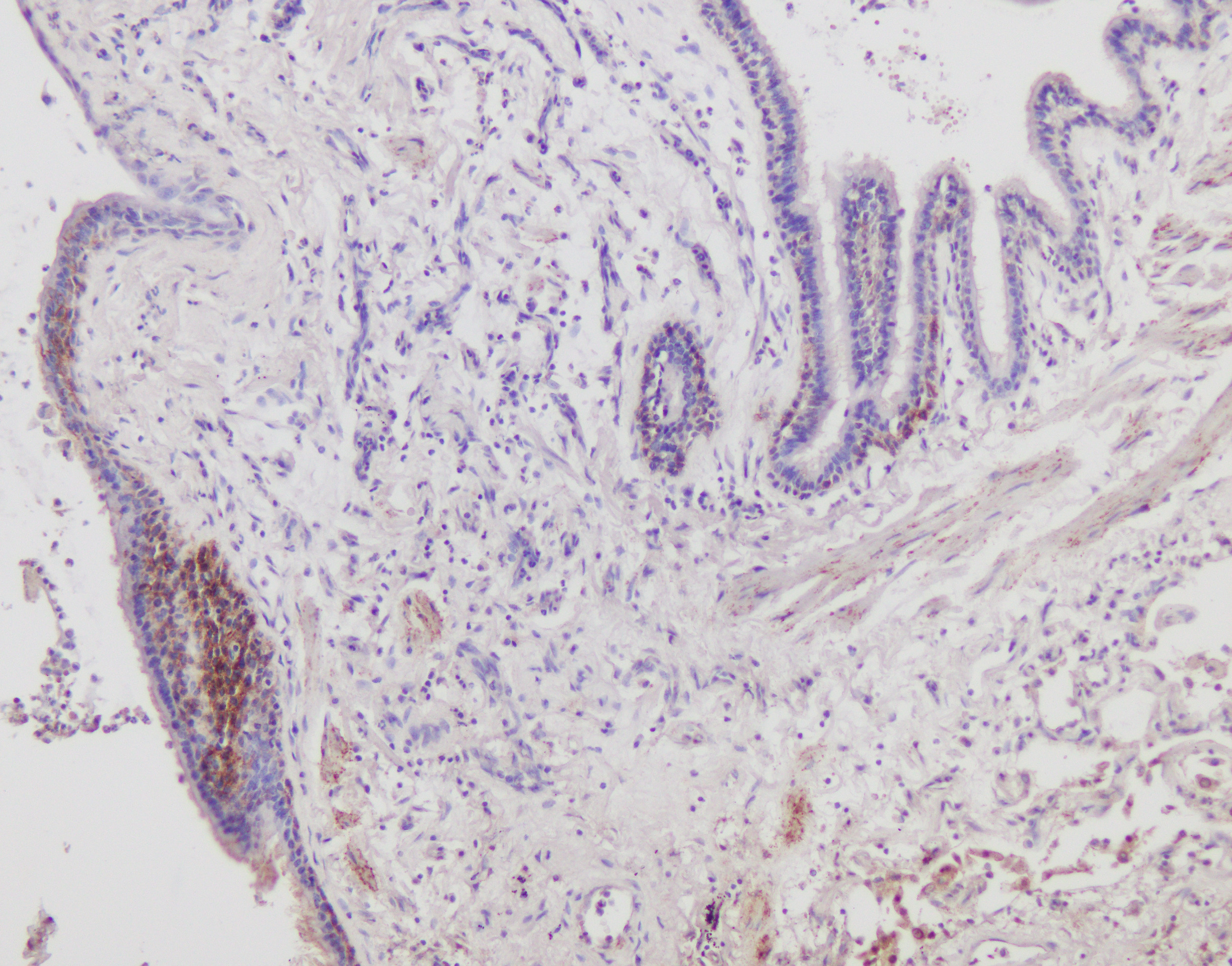

Supplement: Supplementary file 9 [file DataSheet_5.zip › 20X-CDH2-IPF37.jpeg]

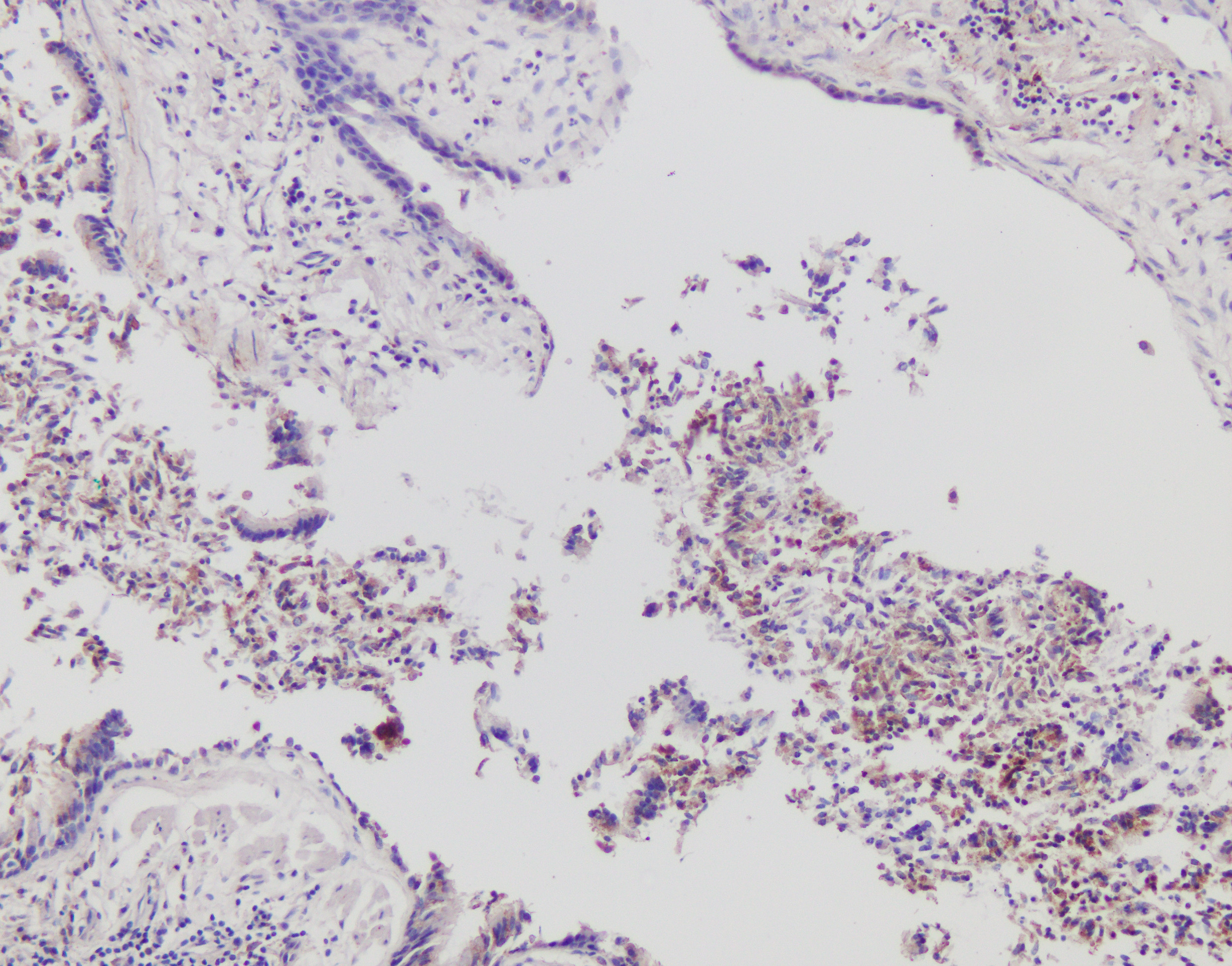

Supplement: Supplementary file 9 [file DataSheet_5.zip › 20X-CDH2-IPF48.jpeg]

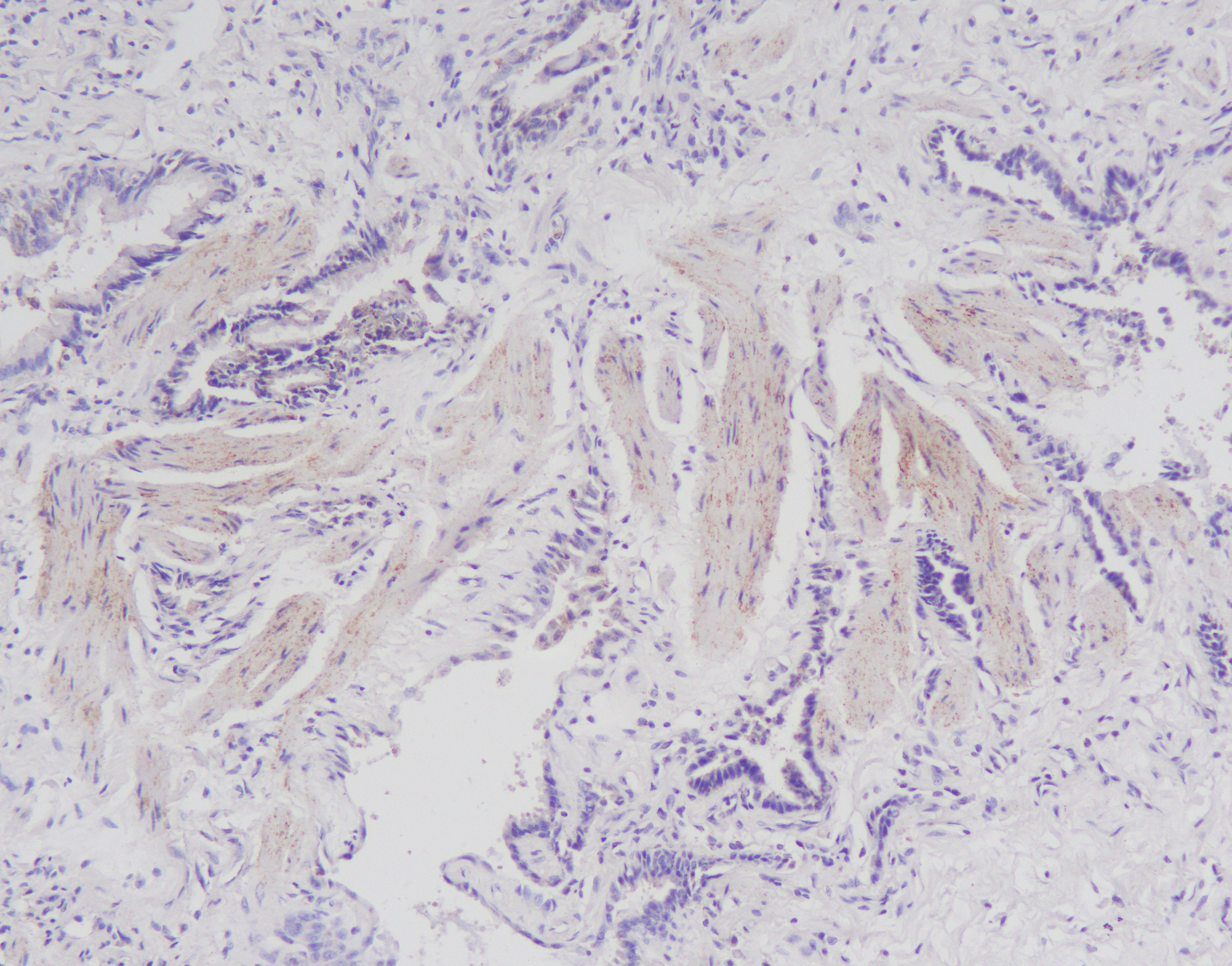

Supplement: Supplementary file 9 [file DataSheet_5.zip › 20X-CDH2-IPF524.jpeg]

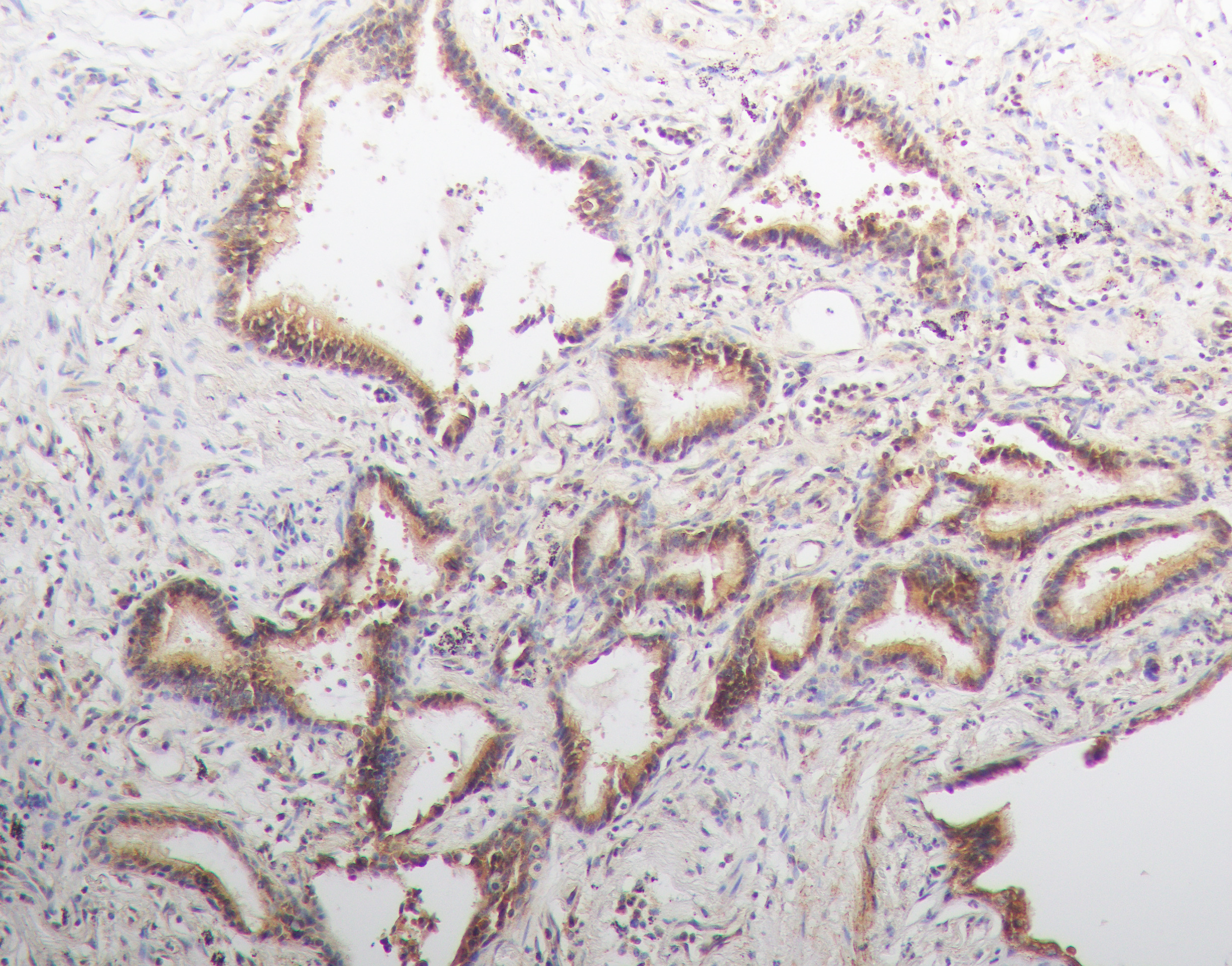

Supplement: Supplementary file 9 [file DataSheet_5.zip › 20X-CDH2-IPF628.jpeg]

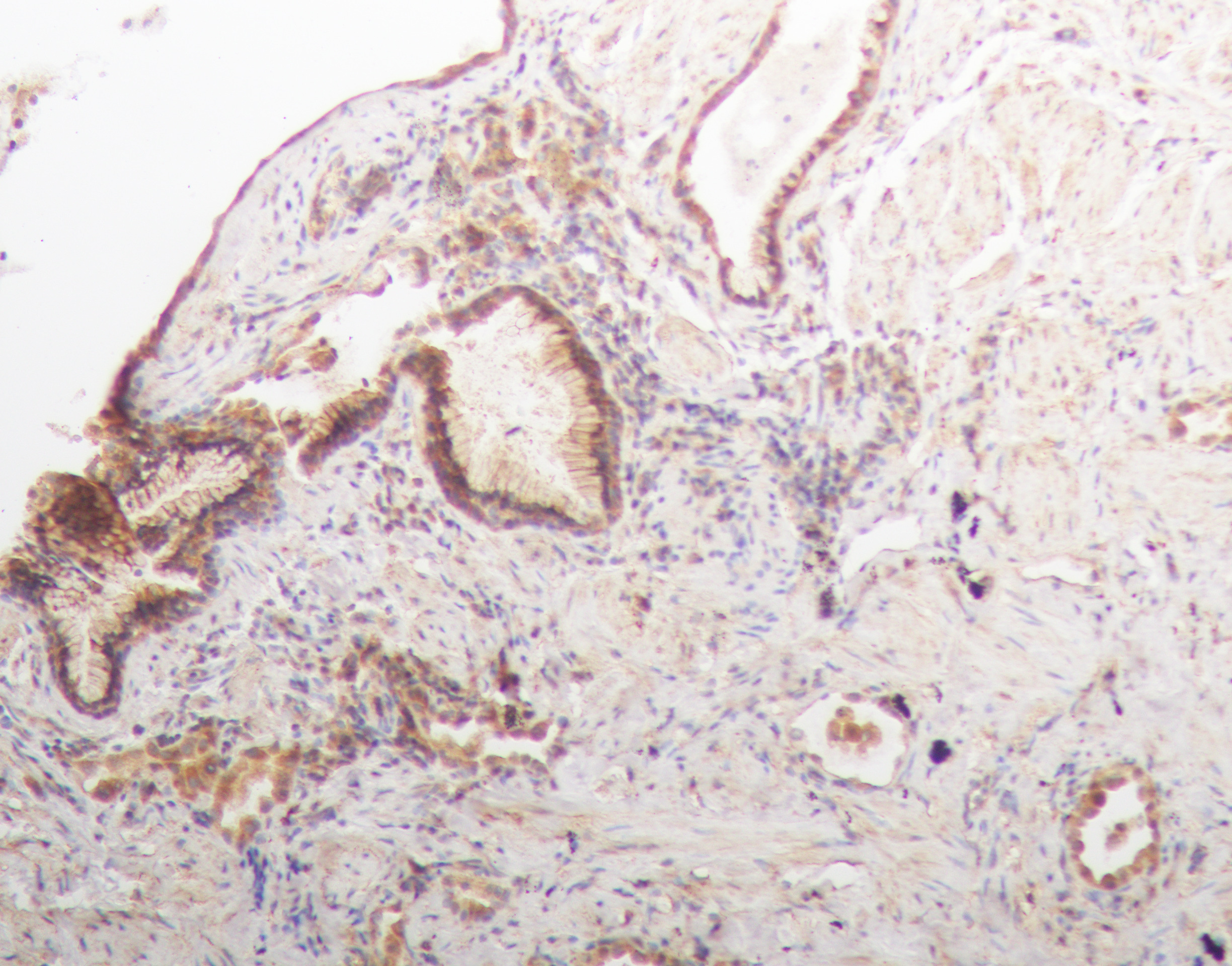

Supplement: Supplementary file 9 [file DataSheet_5.zip › 20X-CDH2-IPF729.jpeg]

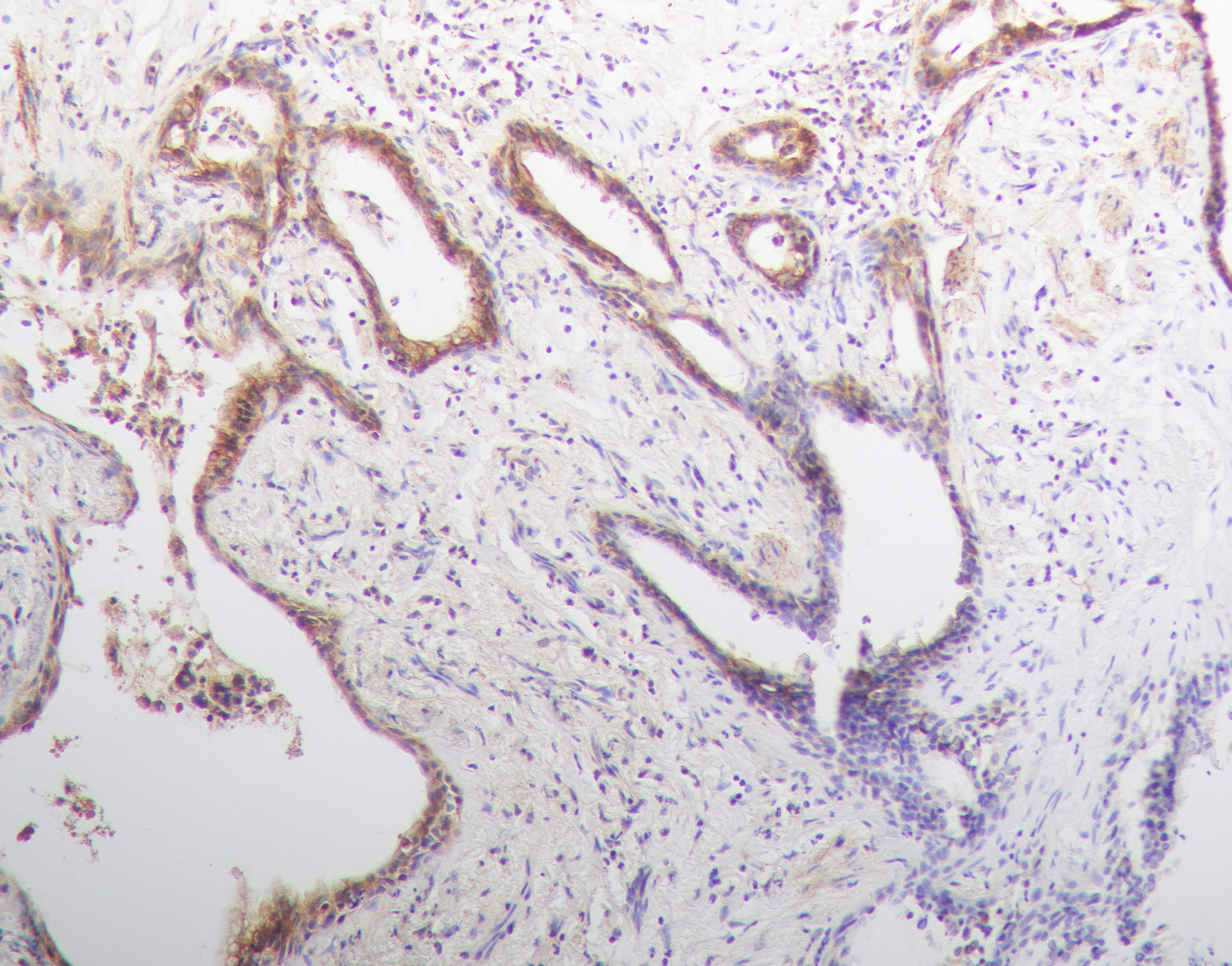

Supplement: Supplementary file 9 [file DataSheet_5.zip › 20X-CDH2-IPF82.jpeg]

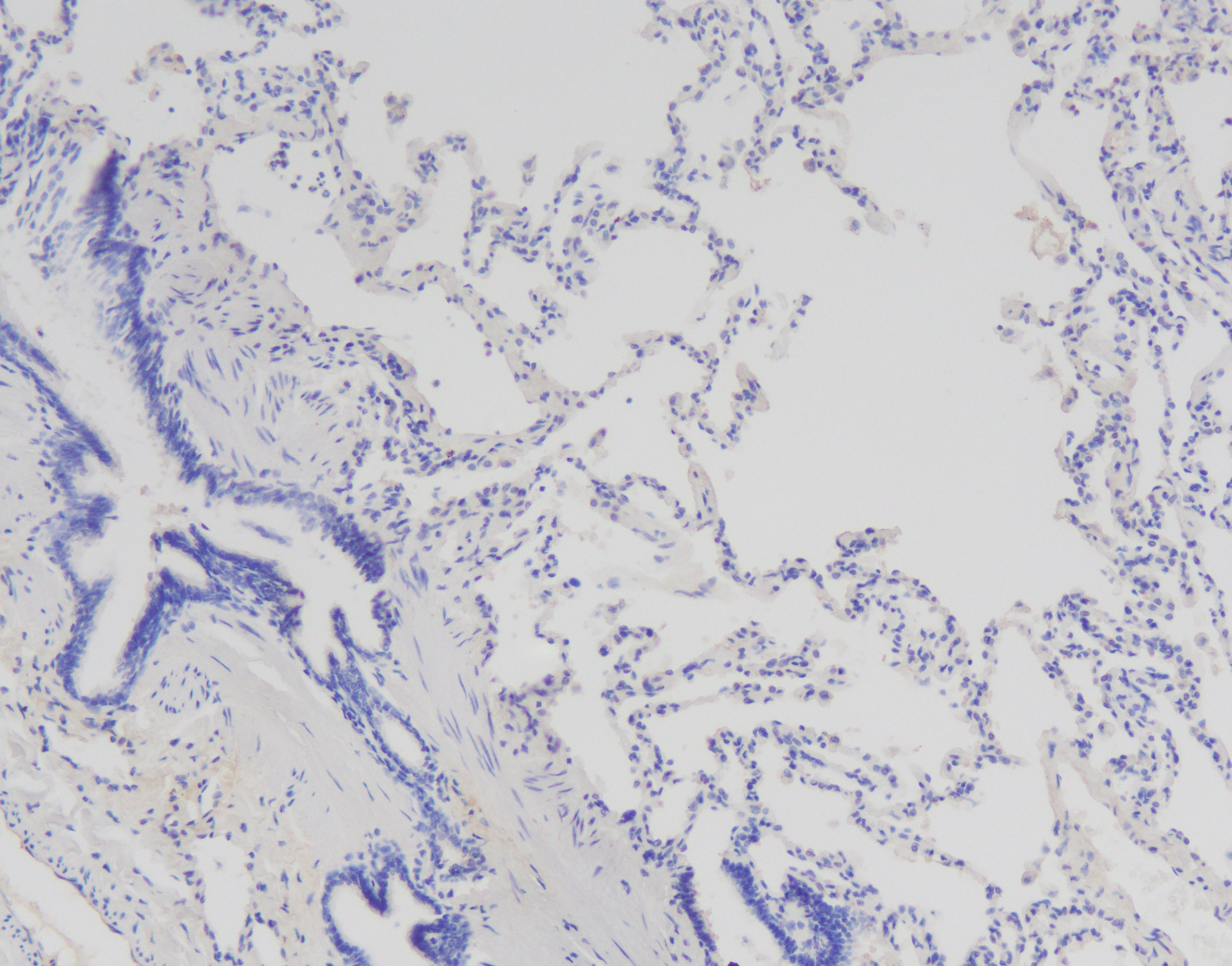

Supplement: Supplementary file 10 [file DataSheet_6.zip › 20X-POSTN-HC130.jpeg]

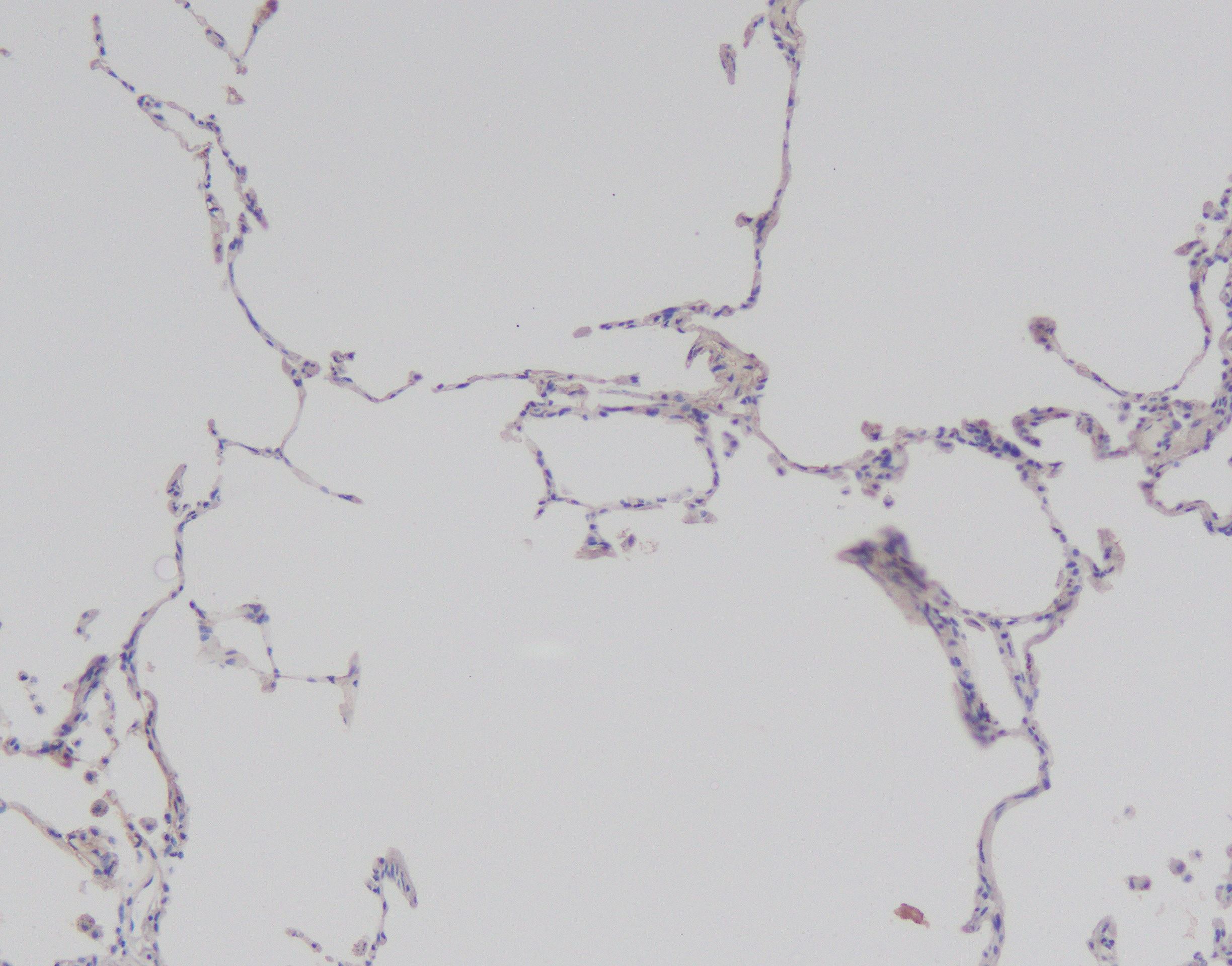

Supplement: Supplementary file 10 [file DataSheet_6.zip › 20X-POSTN-HC231.jpeg]

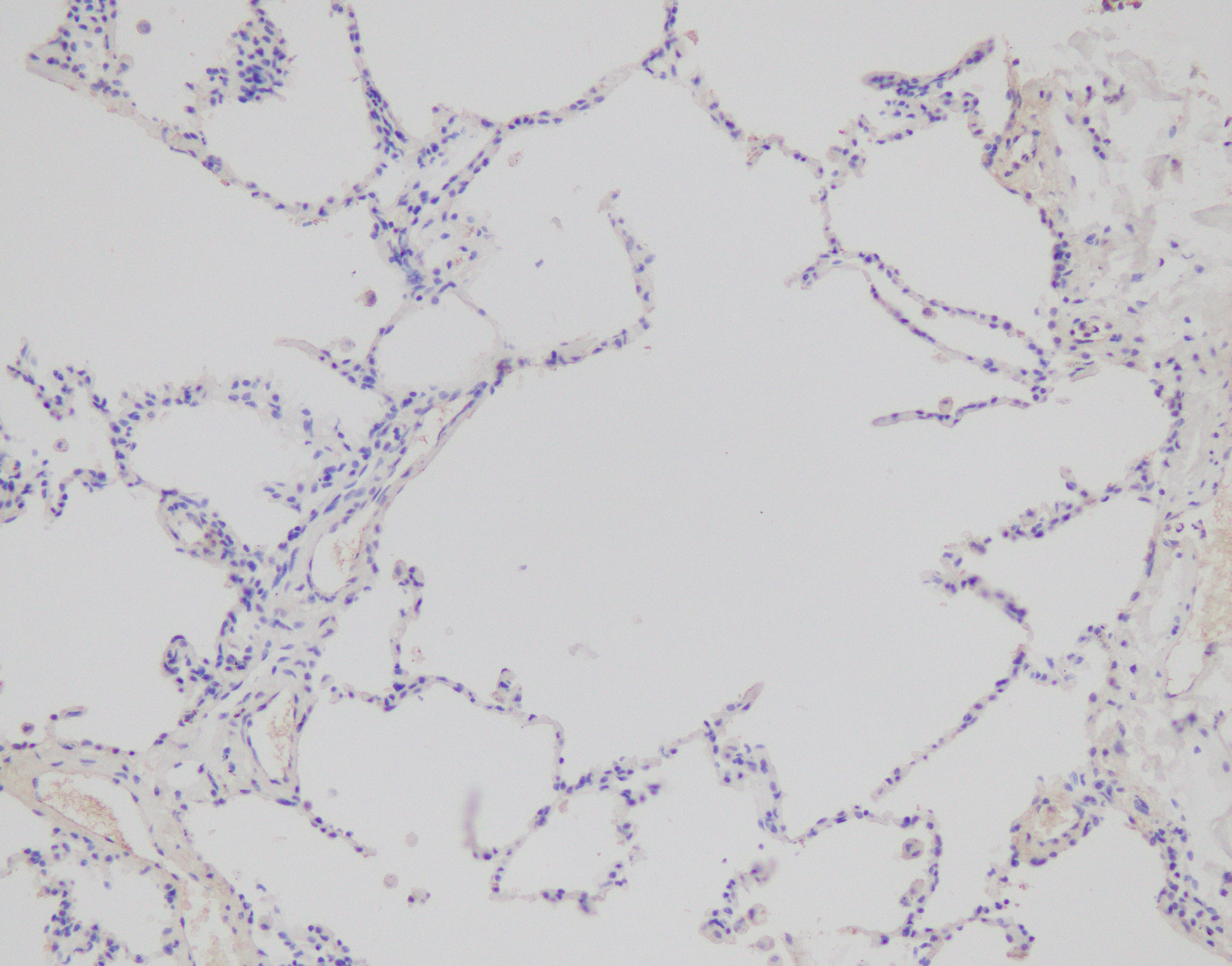

Supplement: Supplementary file 10 [file DataSheet_6.zip › 20X-POSTN-HC318.jpeg]

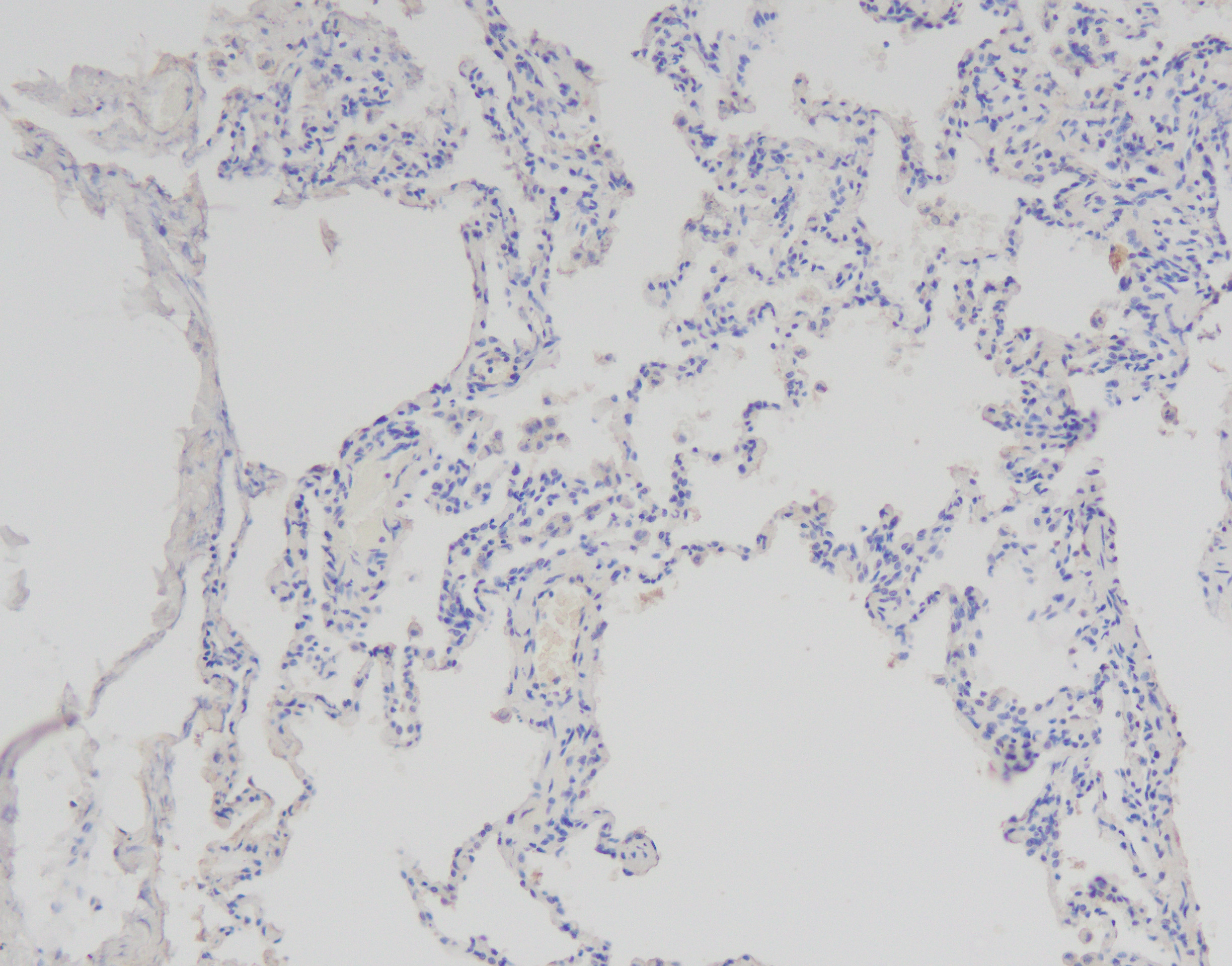

Supplement: Supplementary file 10 [file DataSheet_6.zip › 20X-POSTN-HC432.jpeg]

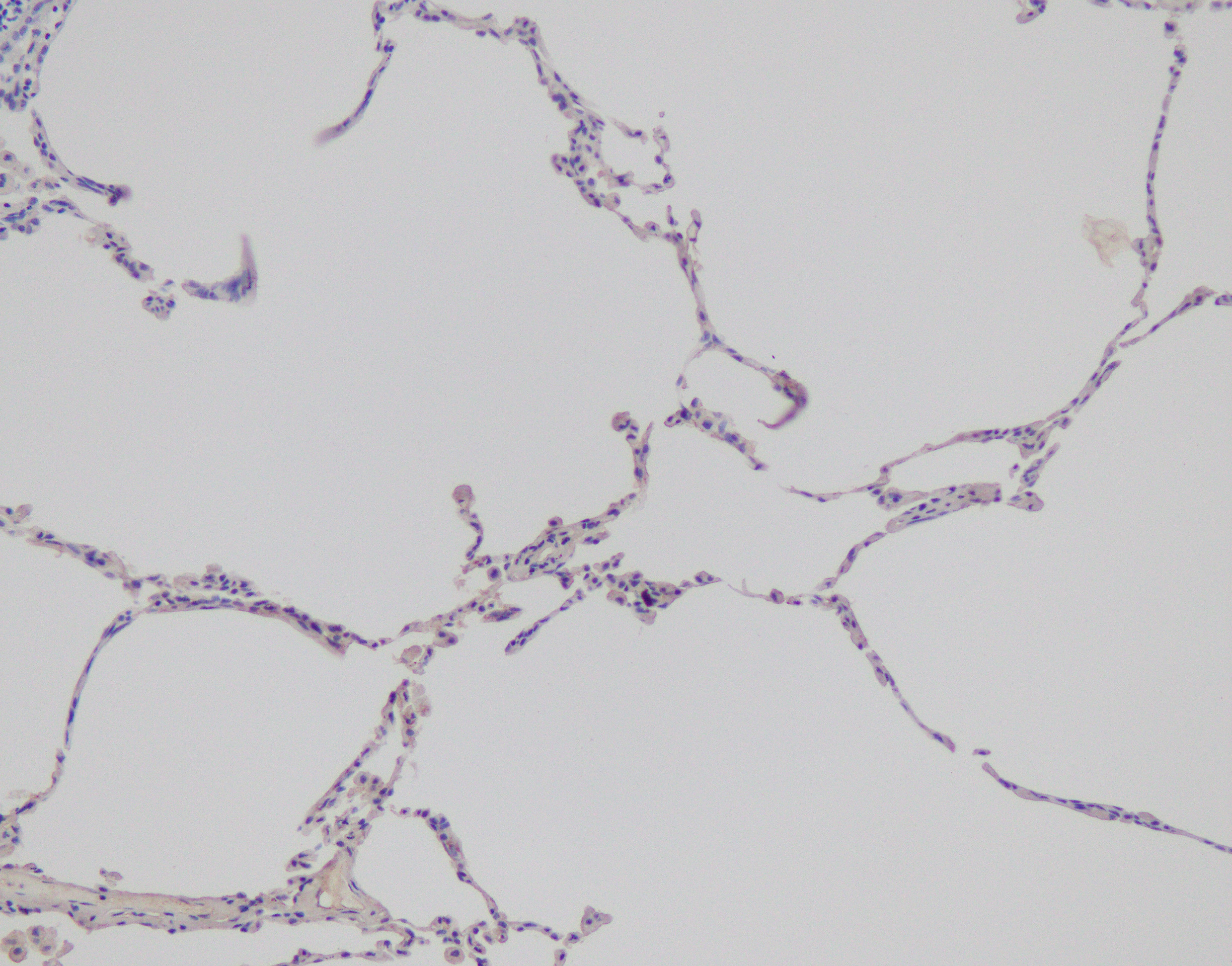

Supplement: Supplementary file 10 [file DataSheet_6.zip › 20X-POSTN-HC519.jpeg]

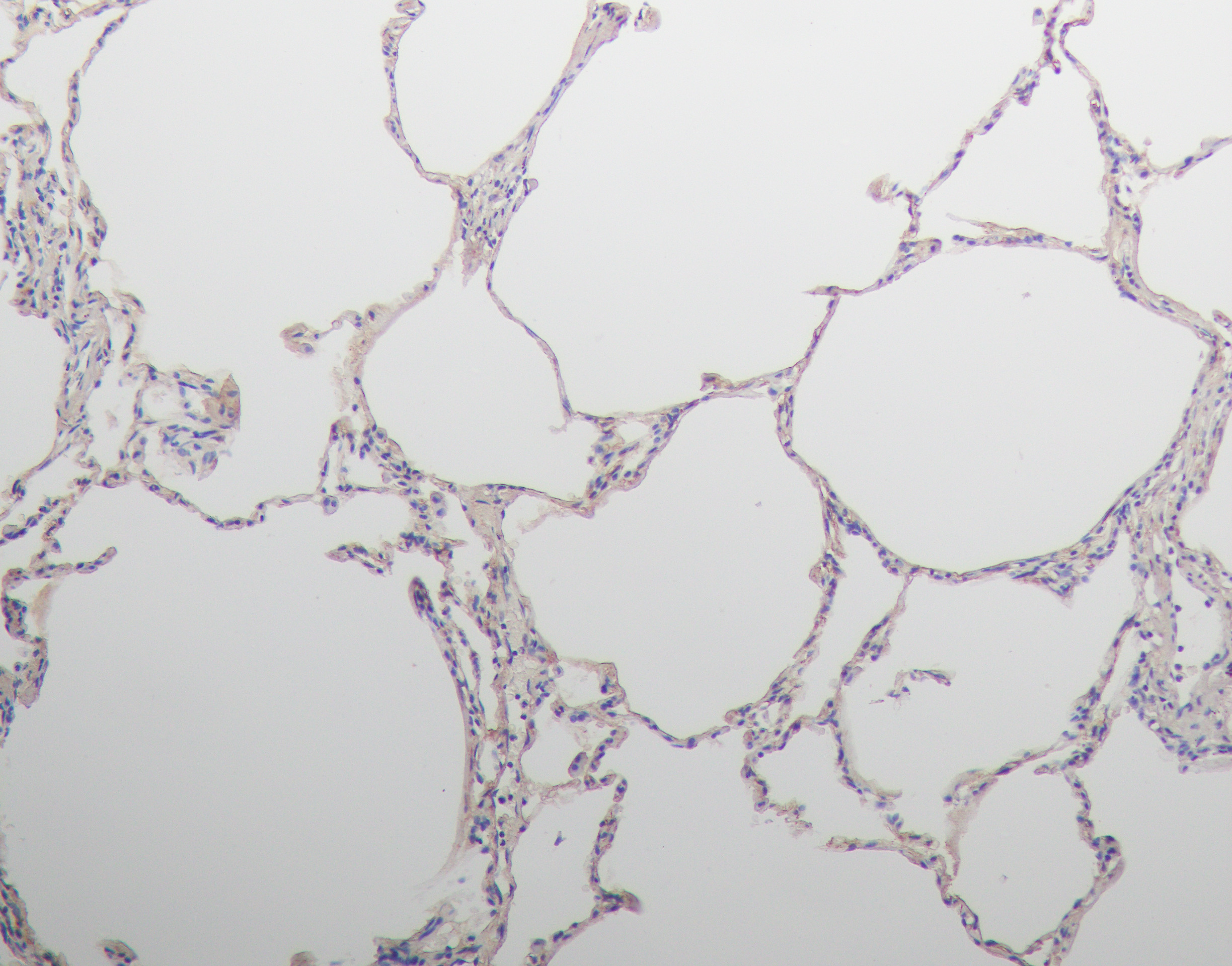

Supplement: Supplementary file 10 [file DataSheet_6.zip › 20X-POSTN-HC633.jpeg]

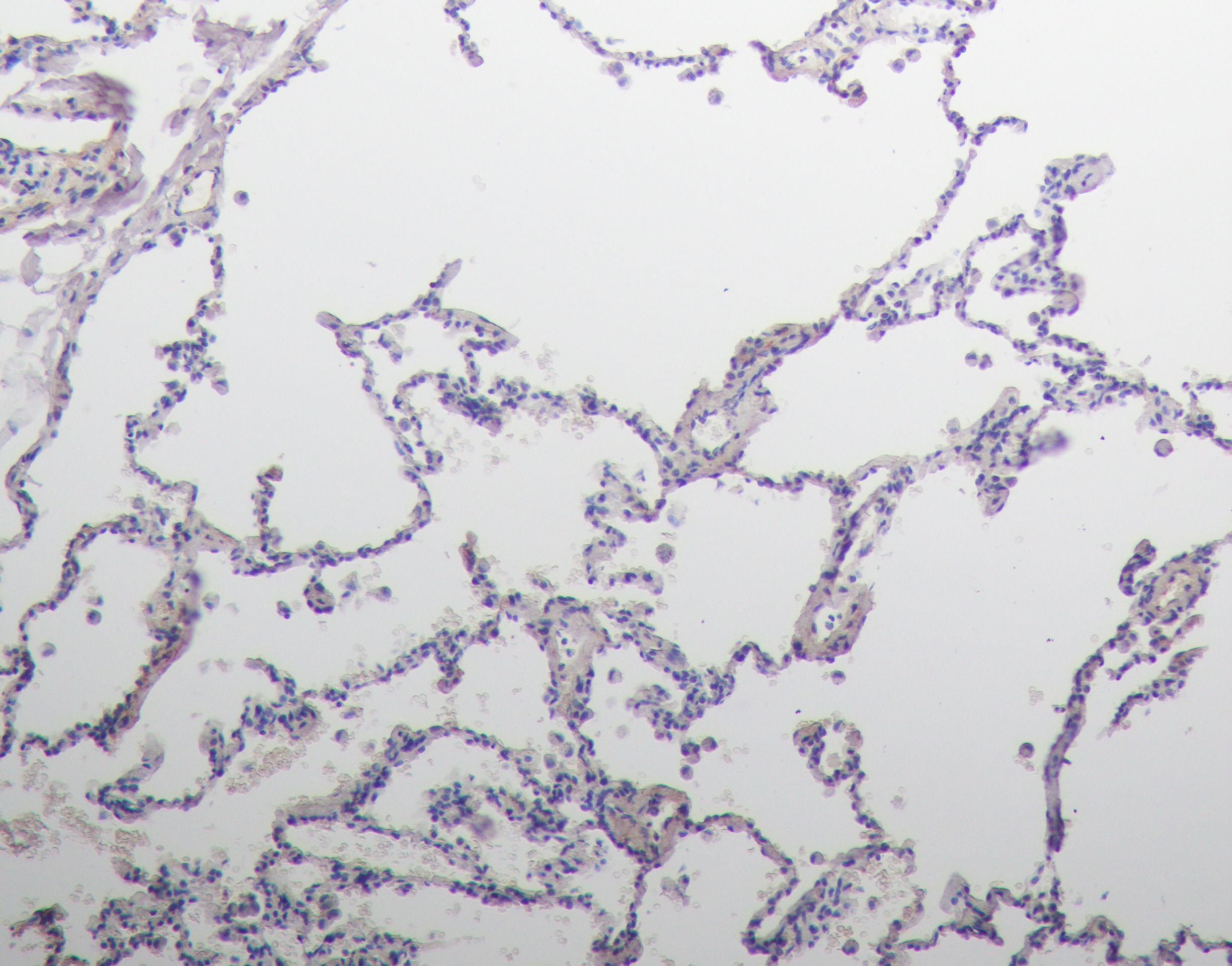

Supplement: Supplementary file 10 [file DataSheet_6.zip › 20X-POSTN-HC734.jpeg]

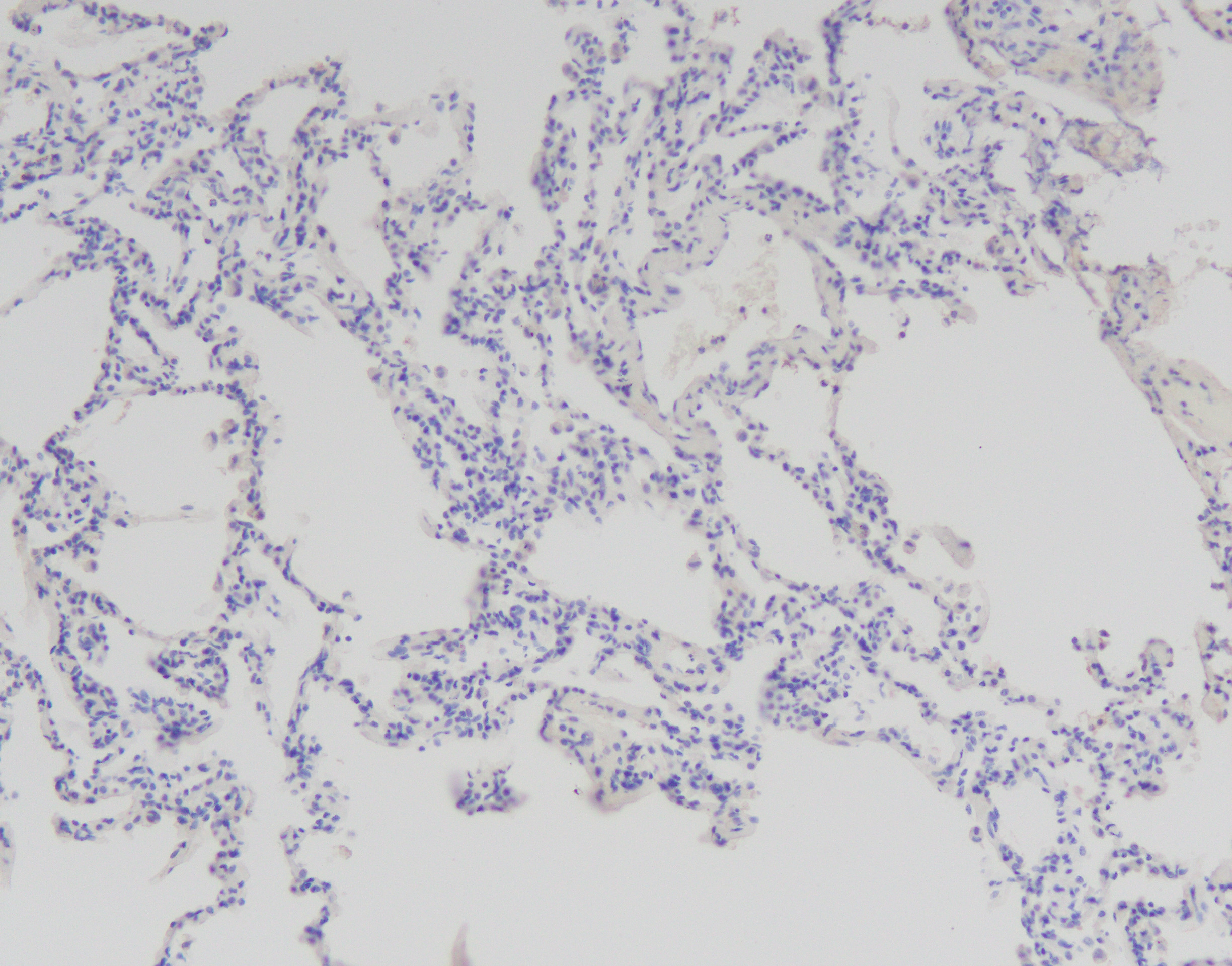

Supplement: Supplementary file 10 [file DataSheet_6.zip › 20X-POSTN-HC835.jpeg]

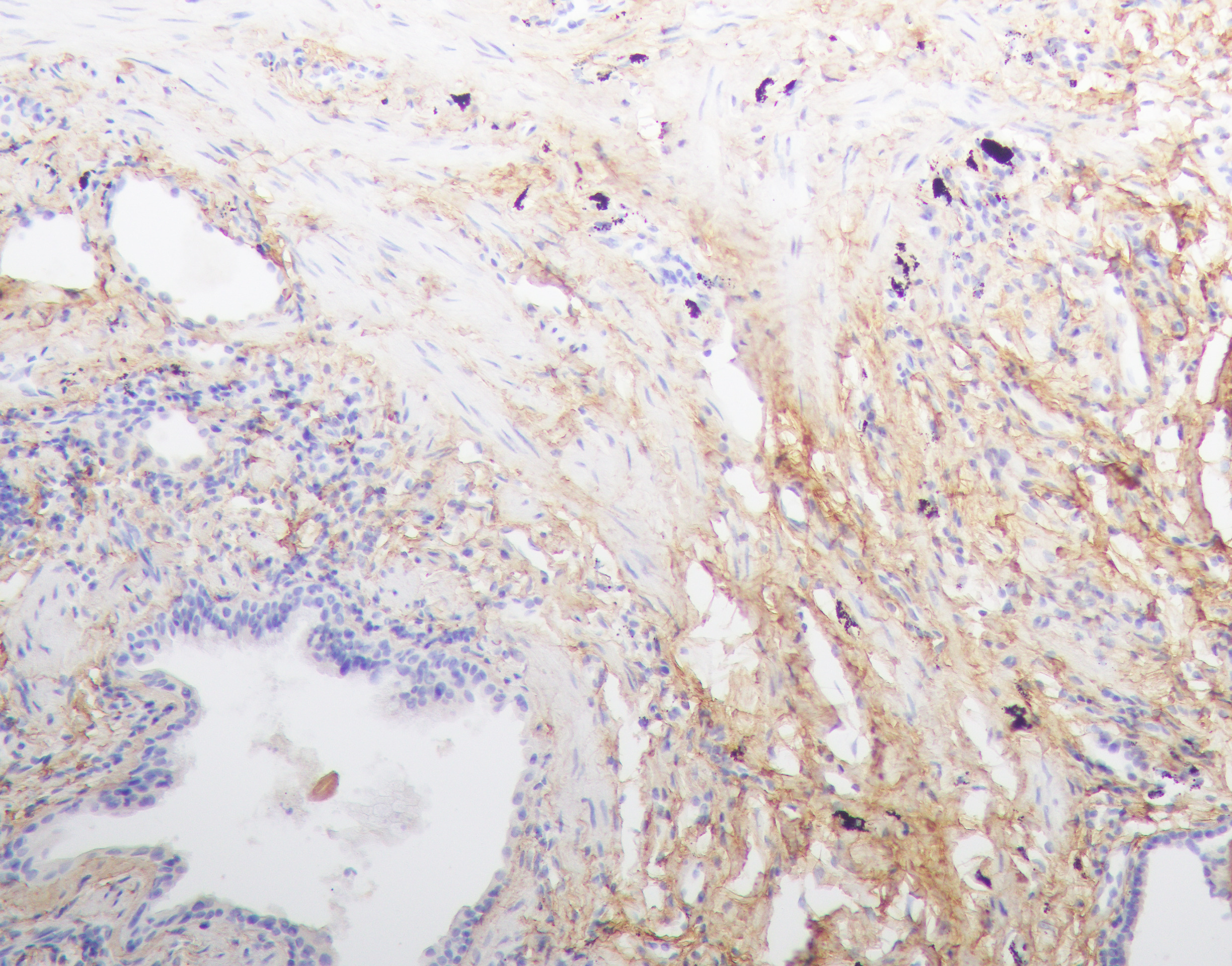

Supplement: Supplementary file 10 [file DataSheet_6.zip › 20X-POSTN-IPF11.jpeg]

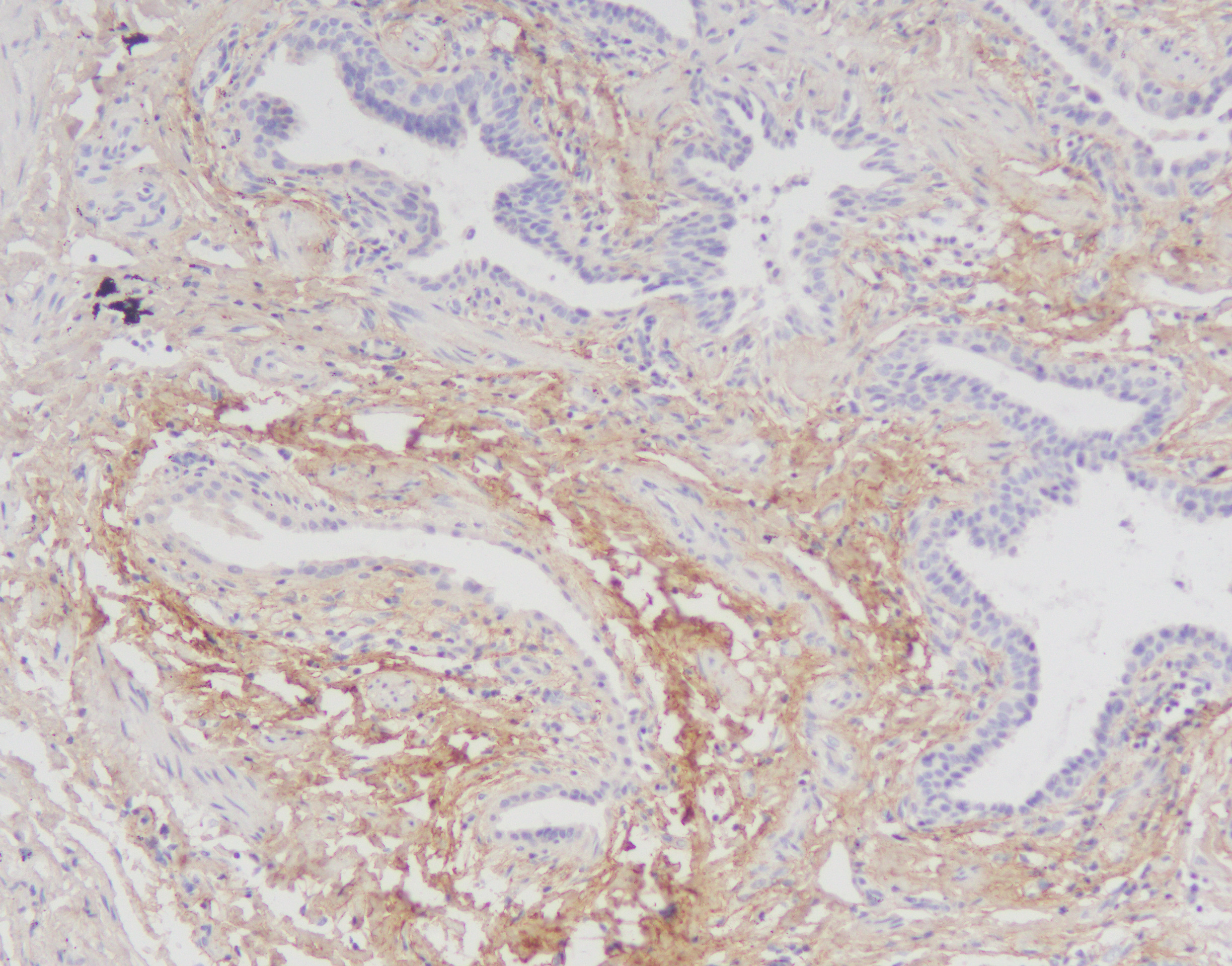

Supplement: Supplementary file 10 [file DataSheet_6.zip › 20X-POSTN-IPF236.jpeg]

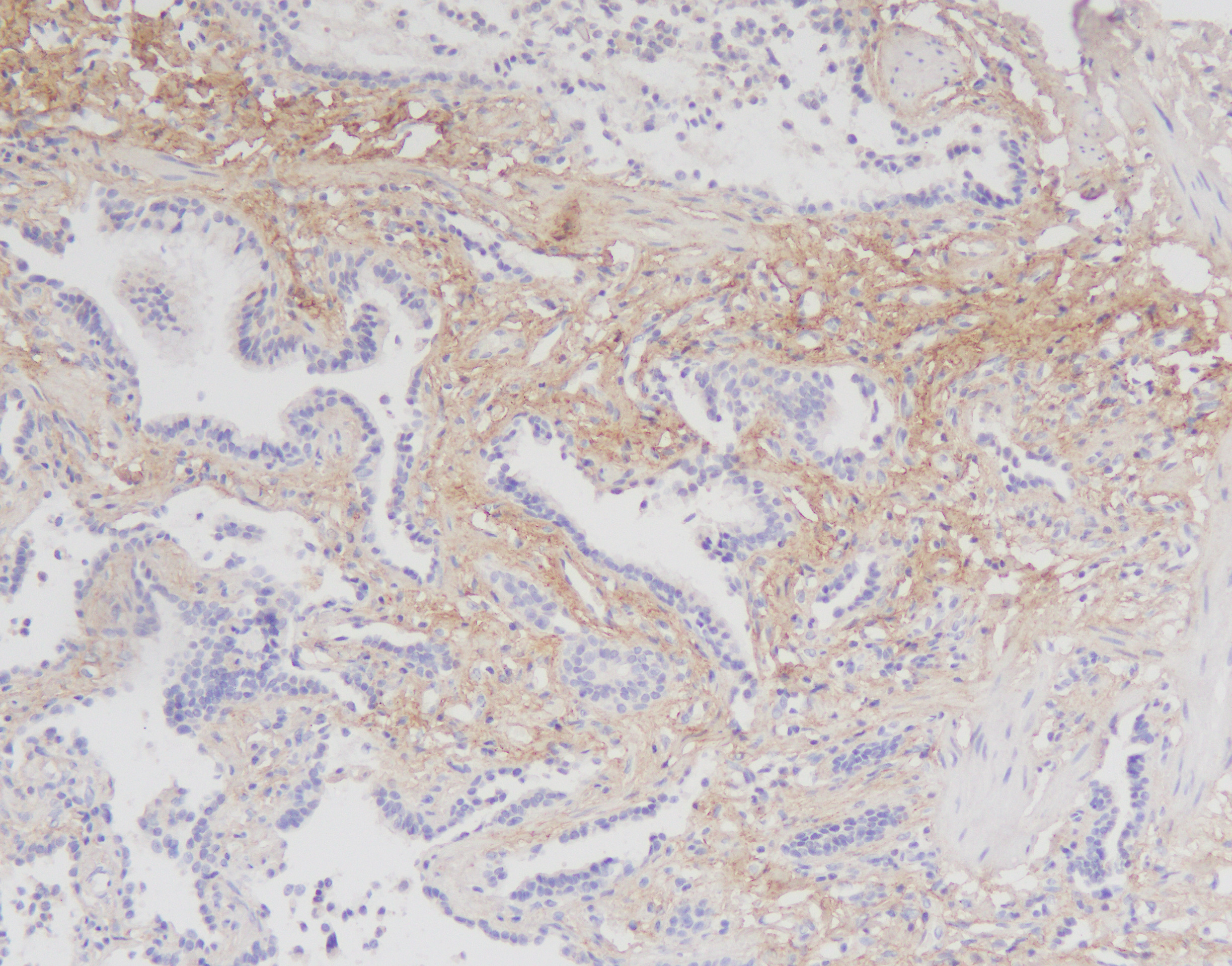

Supplement: Supplementary file 10 [file DataSheet_6.zip › 20X-POSTN-IPF337.jpeg]

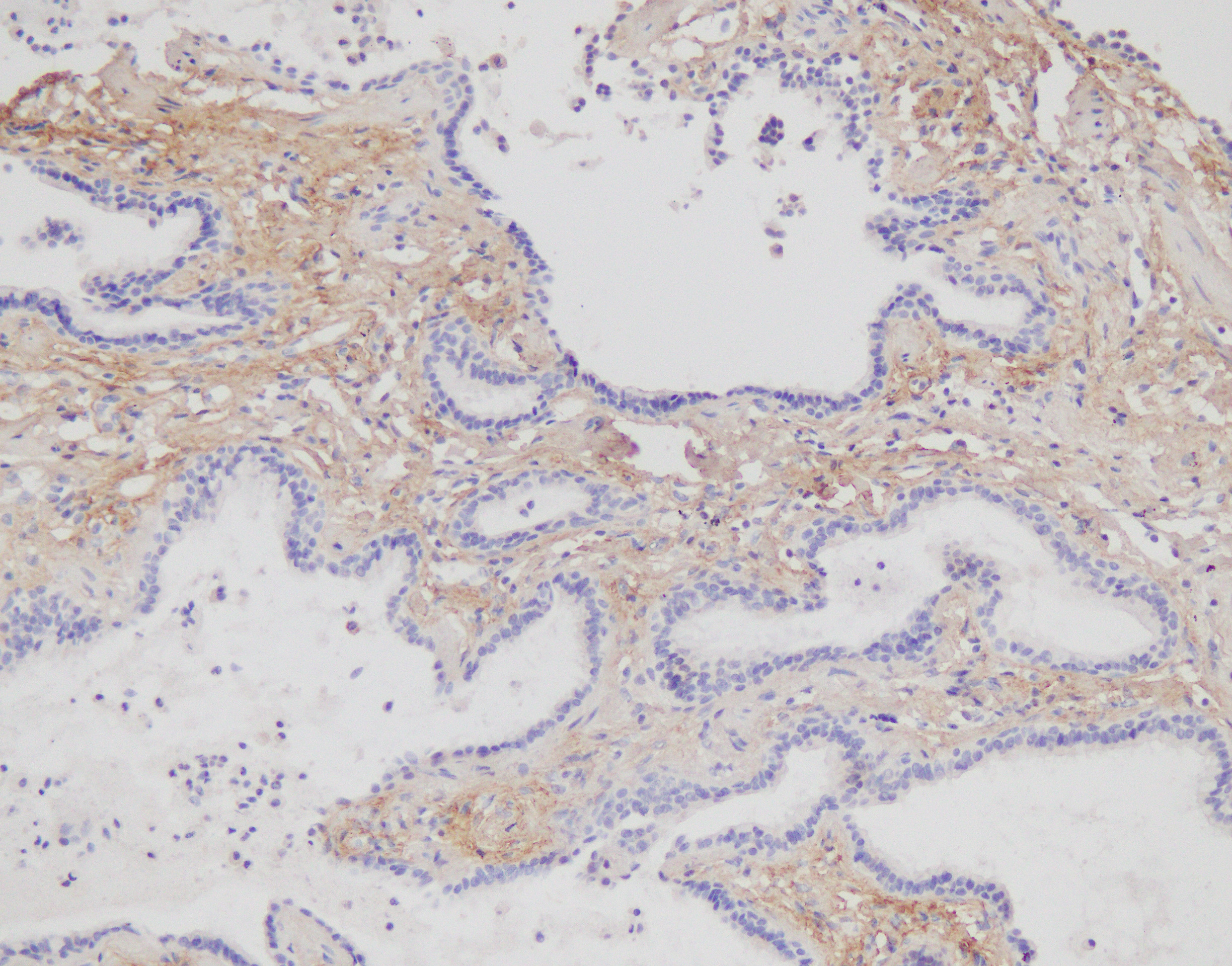

Supplement: Supplementary file 10 [file DataSheet_6.zip › 20X-POSTN-IPF420.jpeg]

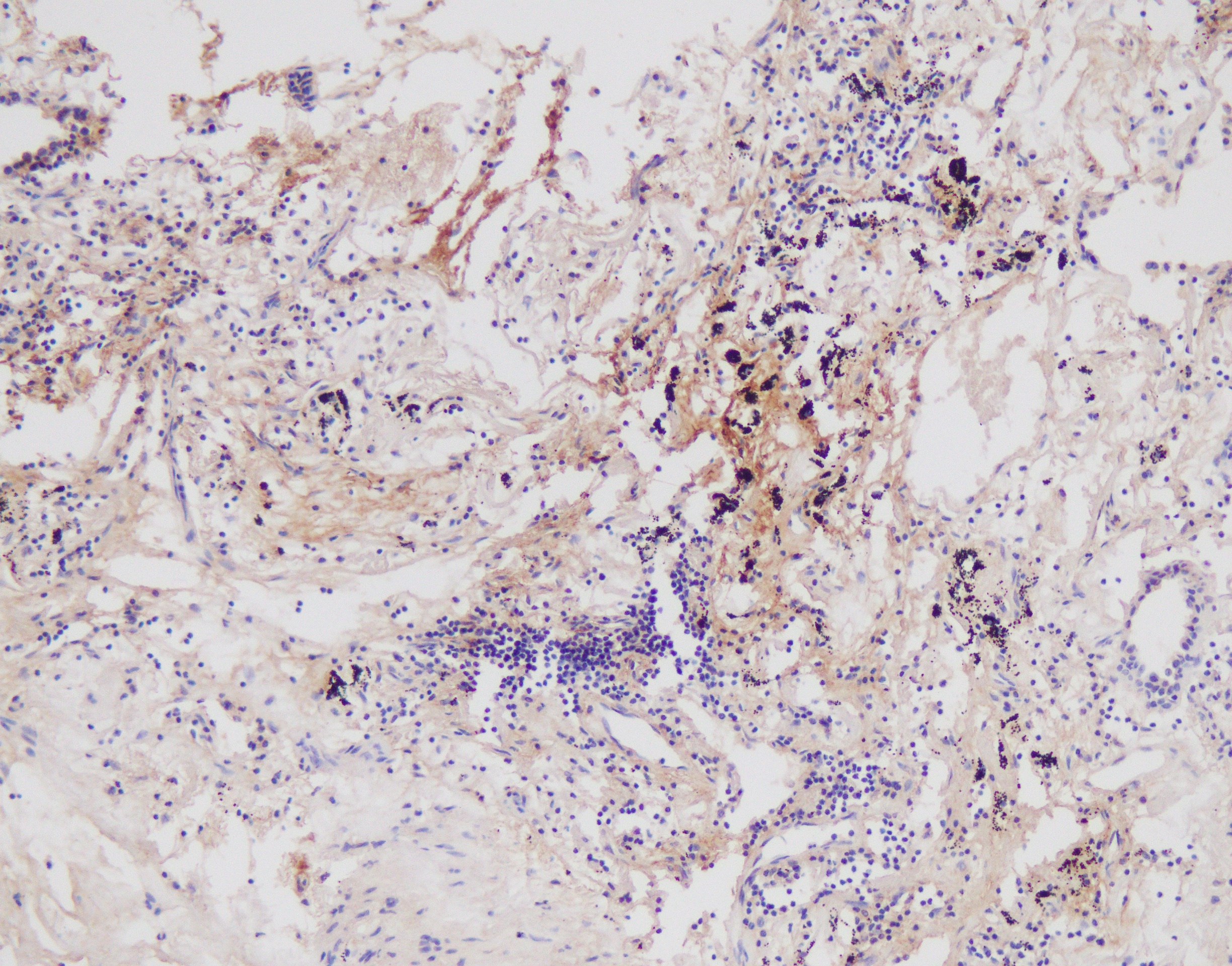

Supplement: Supplementary file 10 [file DataSheet_6.zip › 20X-POSTN-IPF521.jpeg]

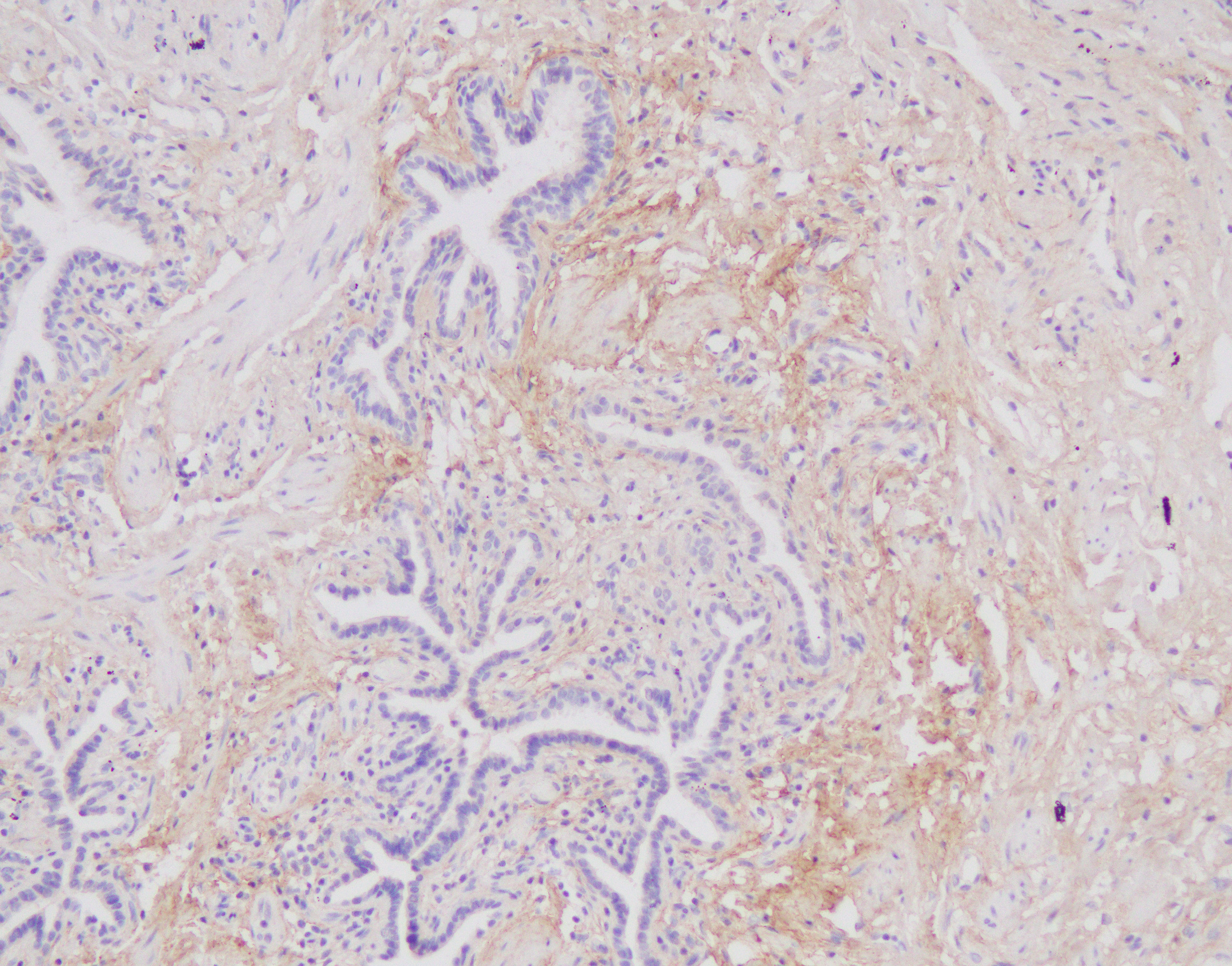

Supplement: Supplementary file 10 [file DataSheet_6.zip › 20X-POSTN-IPF622.jpeg]

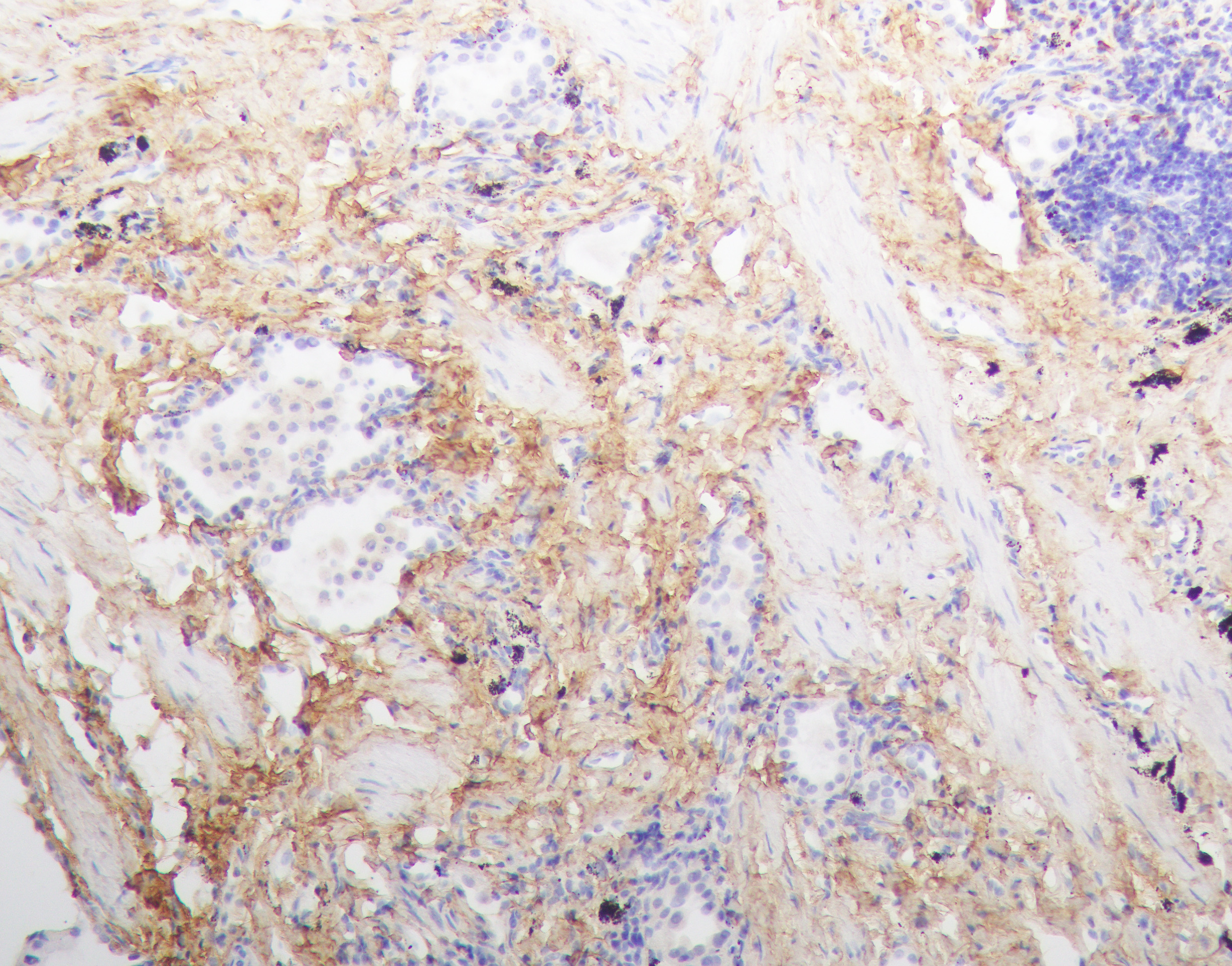

Supplement: Supplementary file 10 [file DataSheet_6.zip › 20X-POSTN-IPF738.jpeg]

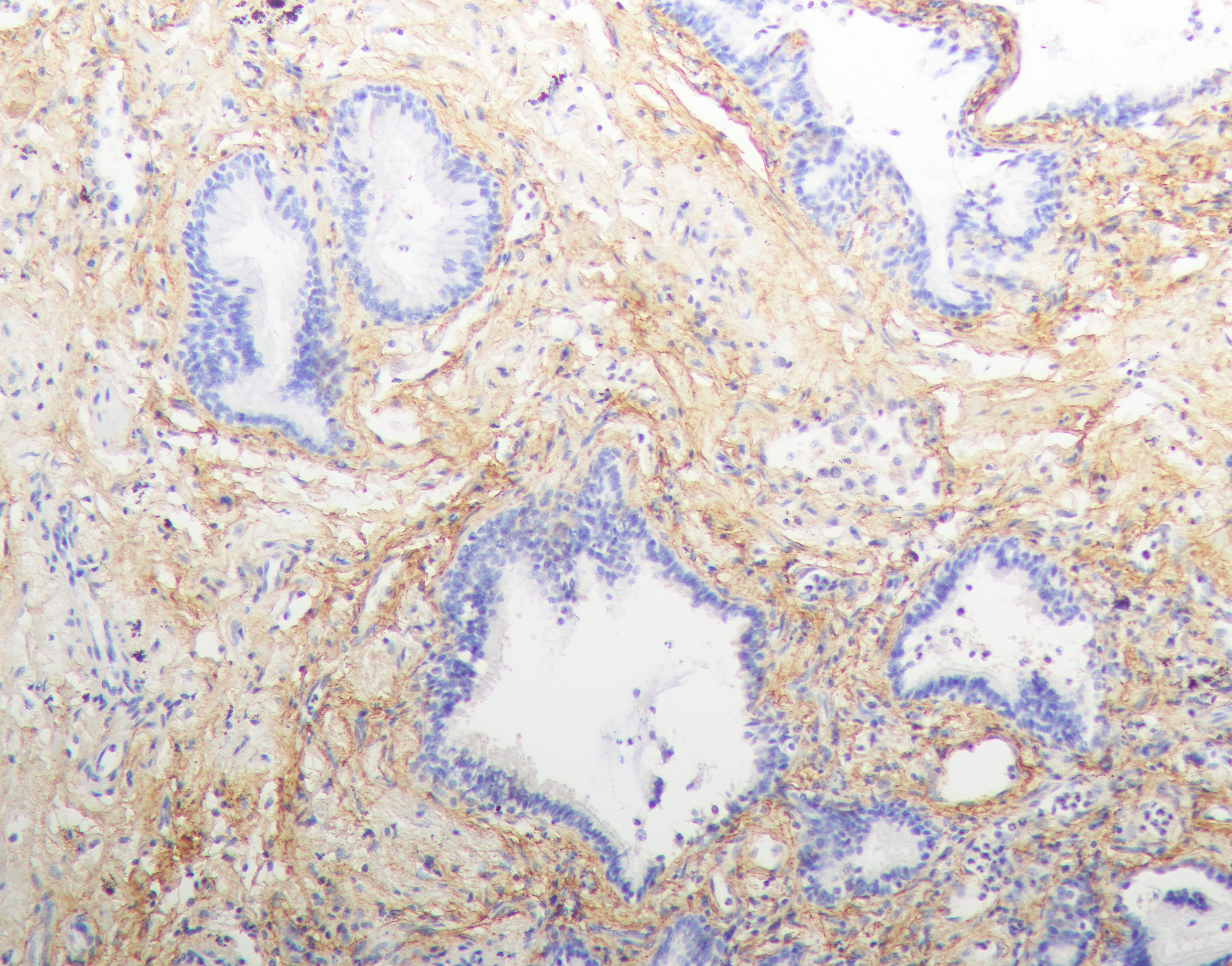

Supplement: Supplementary file 10 [file DataSheet_6.zip › 20X-POSTN-IPF839.jpeg]

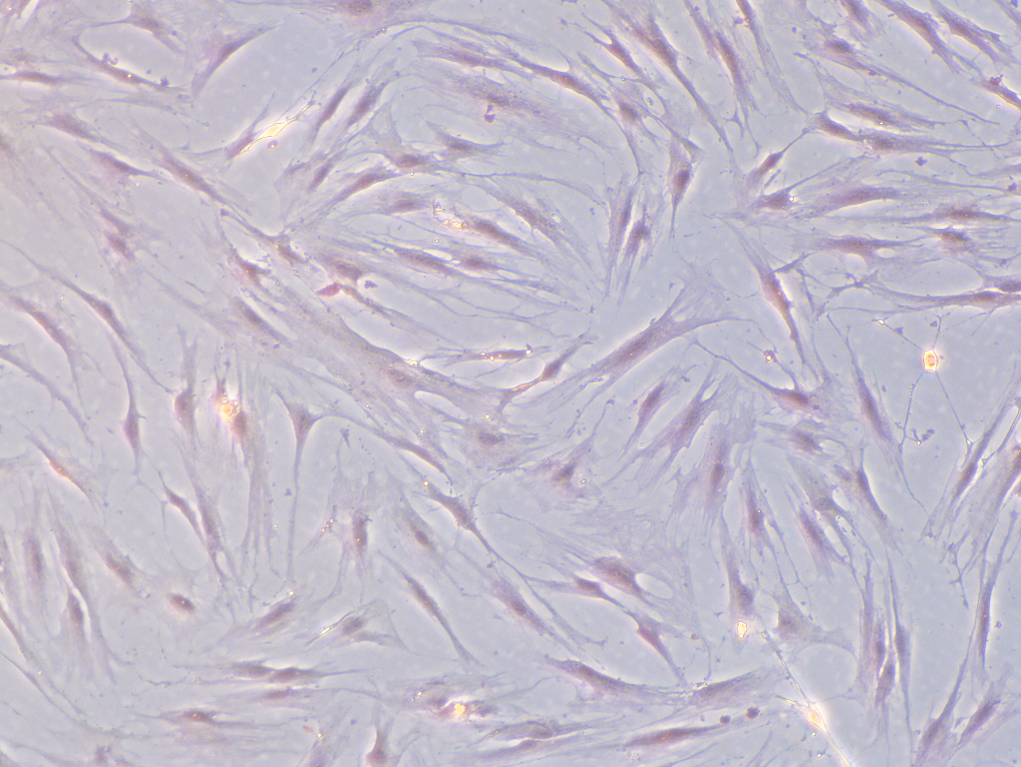

Supplement: Supplementary file 12 [file DataSheet_8.zip › FB+TGF.tif]

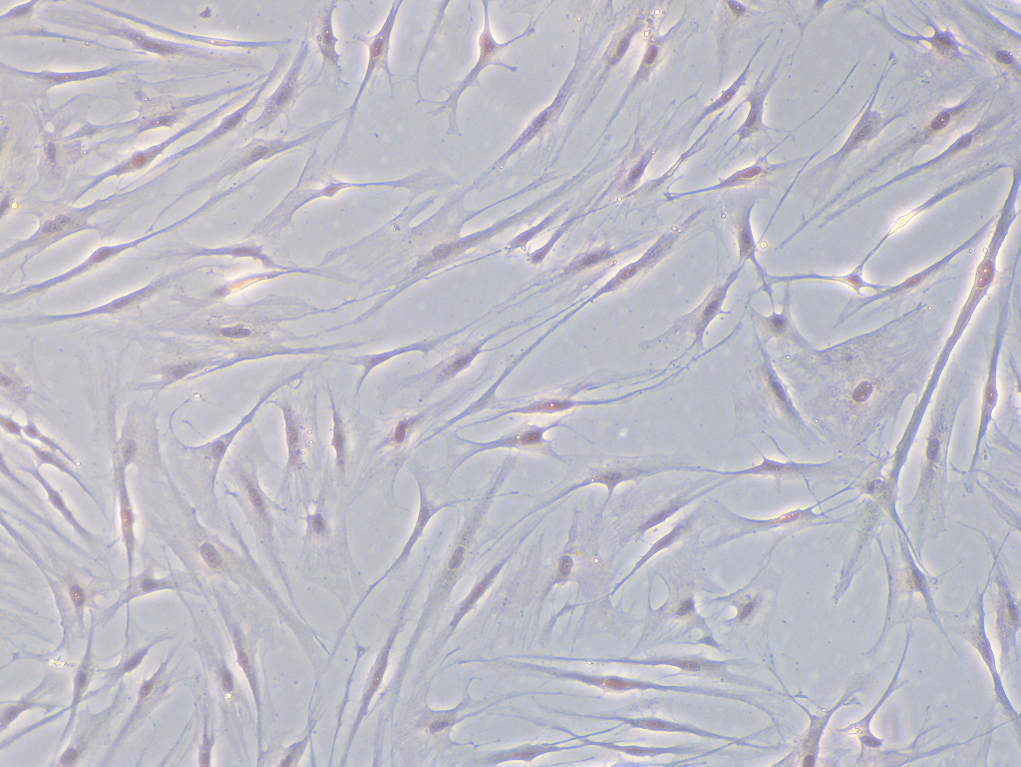

Supplement: Supplementary file 12 [file DataSheet_8.zip › FB.tif]
