# Supplementary material for: The gene expression of CALD1, CDH2, and POSTN in fibroblast are related to idiopathic pulmonary fibrosis
Source: Front Immunol. 2024 Feb 2;15:1275064. doi: 10.3389/fimmu.2024.1275064 (PMC10869495; doi:10.3389/fimmu.2024.1275064)
Supplement: Supplementary file 4 [file DataSheet_1.docx]

Supplementary Methods

**Cell viability assays**

Cell viability was assessed with the Cell Counting Kit-8 (CCK-8; Biosharp). Controlled or transfected cells were plated into 96-well plates at a density of 1×10^3^ cells/well. After incubation for 0, 24, 48, or 72 h, 10 μL of the CCK8 reagent was introduced into each well, followed by an additional incubation at 37 °C for 2 hours. Subsequently, the absorbance rate was measured at 450 nm using a microplate spectrophotometer.

**Sirius red and H&E staining**

The lung fibroblast were embedded in paraffin and stained with Sirius-Red staining (PH1098, PHYGENE). Lung specimens were embedded in paraffin and cut into 4 μm-thick slices. The tissue sections were stained with hematoxylin and eosin (H&E) staining (C0105S, Beyotime).
