# Supplementary figures and images for: The gene expression of CALD1, CDH2, and POSTN in fibroblast are related to idiopathic pulmonary fibrosis (part 2 of 2)
Source: Front Immunol. 2024 Feb 2;15:1275064. doi: 10.3389/fimmu.2024.1275064 (PMC10869495; doi:10.3389/fimmu.2024.1275064)

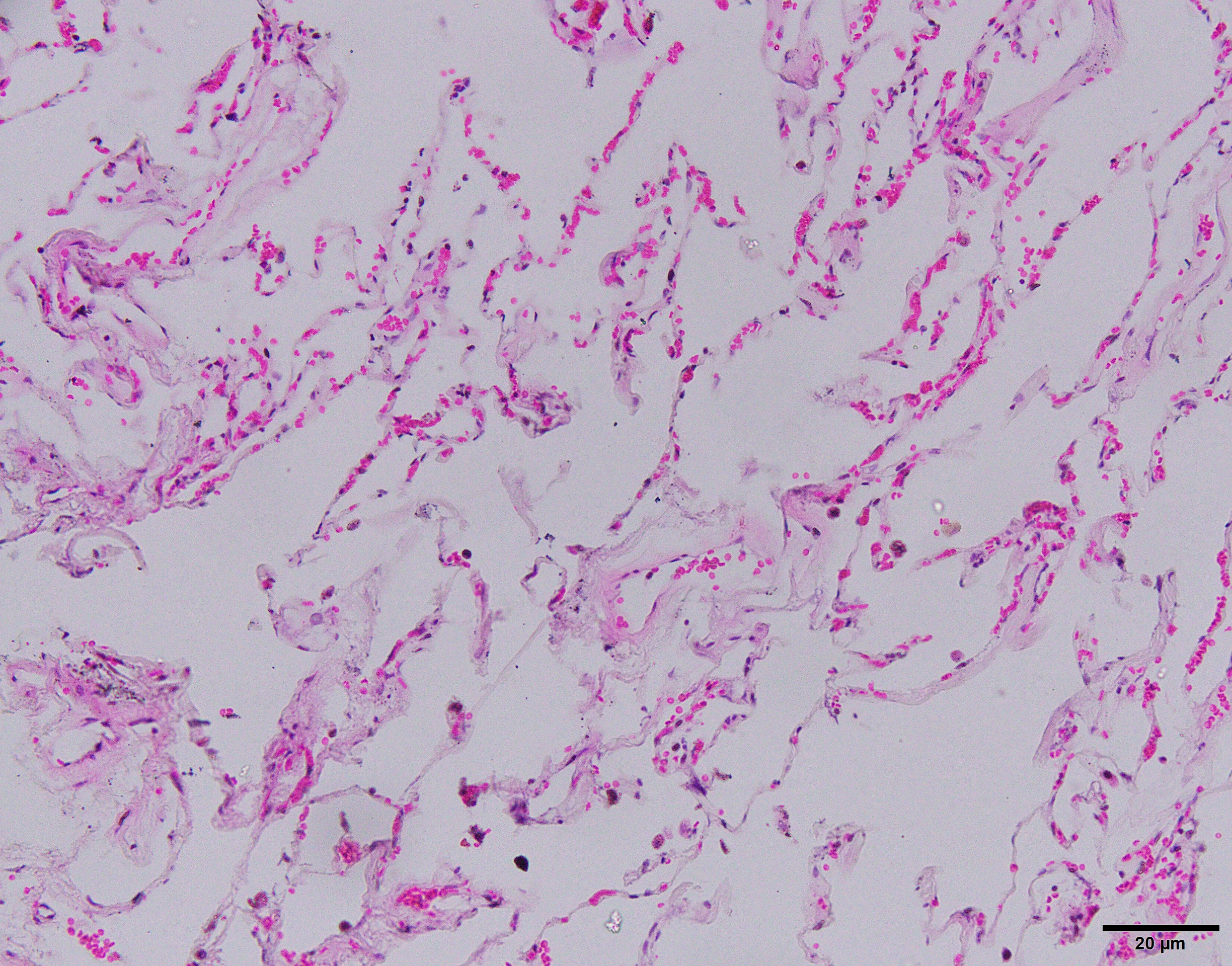

Supplement: Supplementary file 12 [file DataSheet_8.zip › HE-HC.jpg]

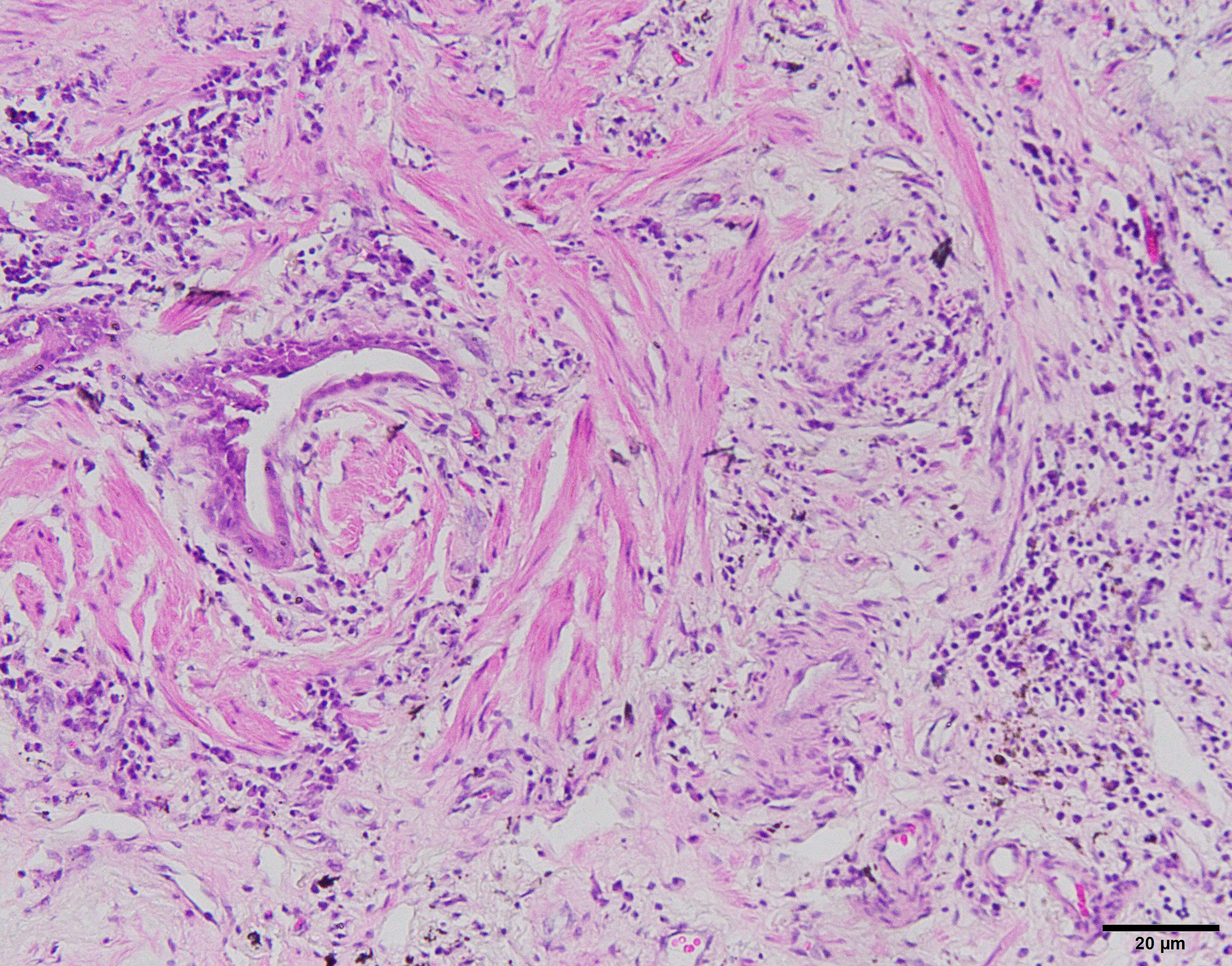

Supplement: Supplementary file 12 [file DataSheet_8.zip › HE-IPF.jpg]
